# A Comprehensive Account of the Breeding Systems of the Fire Ant *Solenopsis invicta*

**DOI:** 10.1002/ece3.71888

**Published:** 2025-08-25

**Authors:** Sierra Hale Walker, Kip D. Lacy, Kenneth G. Ross, Haolin Zeng

**Affiliations:** ^1^ Department of Entomology University of Georgia Athens Georgia USA; ^2^ Laboratory of Social Evolution and Behavior The Rockefeller University New York New York USA; ^3^ Odum School of Ecology University of Georgia Athens Georgia USA

**Keywords:** breeding/mating systems, genetic conflict, polyandry, reproductive skew, social organization, supergene

## Abstract

When animals reproduce in social groups, the potential for conflict and cooperation is shaped by the number of reproductive individuals (breeders), their relatedness to one another, and division of reproduction among them. These features comprise species' “breeding systems.” Despite their importance, breeding systems are poorly characterized in most social animals, and detailed accounts for single species are rare. Here, we comprehensively characterize the breeding systems of the fire ant *Solenopsis invicta* , an invasive species in which a large genetic element (supergene) determines whether a colony has a single queen (monogyne social form) or multiple queens (polygyne form). Colonies of the monogyne form are simple families, and the breeding system is correspondingly straightforward. The breeding system of the polygyne form is complex, with many features still not well characterized. We conducted a large longitudinal experiment tracking parentage, relatedness, and supergene genotype in laboratory‐reared polygyne colonies. Along with reanalyzed data from previous studies, we show that colony queen number is highly variable, queens generally mate once, nestmate breeders (queens and their mates) generally are unrelated, and reproductive skew is pervasive, especially for parentage of sexual daughters. Uncommon instances of polyandry occur when a queen remates after initially mating with a male bearing the *Sb* supergene haplotype (associated with small size and low sperm counts). Paternity skew is pronounced and stable, with *Sb* sperm contributing to a minority of offspring (particularly sexual daughters) of polyandrous queens. The supergene thus not only determines colony queen number, it broadly affects the breeding system, impacting colony kin structure and setting the stage for conflict and cooperation in the colony. This study can serve as a template for studies of the constellation of factors that affect group genetic structure in other social animals.

## Graphical abstract

We comprehensively characterized the breeding system of polygyne fire ant colonies by tracking parentage, relatedness, and supergene genotypes in a large‐scale, longitudinal experiment. Our results reveal high variation in queen number, generally unrelated nestmate breeders, rare polyandry, and pronounced reproductive and paternity skew shaped by the supergene.

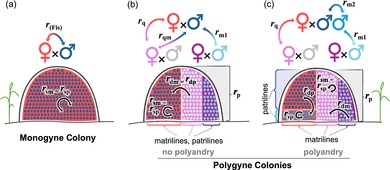

## 1 Introduction

Our understanding of animal life is incomplete without documentation of the rich diversity of societies that have evolved (Clutton‐Brock 2021; Linksvayer and Johnson 2019; Rubenstein and Abbot 2017). For animals that reproduce in durable social groups, understanding how these groups form and are structured informs fields as diverse as conservation biology, public health, pest management, and global climate change (Elsner‐Gearing et al. 2024; Eyer and Vargo 2021; Helanterä et al. 2009; Kronauer 2023; Modi et al. 2022; Rabitsch 2011; Suarez and Goodisman 2021). Moreover, continued progress in unveiling the evolutionary causes and consequences of animal social behavior relies on detailed studies of social group composition and the distribution of reproduction among group members (Boomsma 2009; Bourke 2015; Chapuisat 2023; Schneider et al. 2017).

The number of breeding individuals in a social group, how they are related, and how reproduction is shared among them collectively comprise what is termed the “breeding system” (Aron et al. 2016; Azevedo‐Silva et al. 2020; Chapuisat and Keller 1999; Eyer et al. 2020; Haag‐Liautard et al. 2008; Hannonen et al. 2004; Masoni et al. 2019; Purcell and Chapuisat 2013; Ross 1993, 2001; Thurin et al. 2011; Wade 1985). The specific form of the breeding system determines group composition and the distribution of genetic variation within and between families, subfamilies, and/or other subgroups not based on kinship (Ross 2001). Comprehension of social evolution hinges on recognizing how historical selection pressures, particularly those tied to group kin structure, shape interactions among group members. These pressures shape the dynamics of intra‐colony conflict and cooperation, altering the equilibrium between factors fostering group cohesion and those favoring selfish or nepotistic behaviors (Boomsma 2009; Oi et al. 2021; Pamilo et al. 1997). Moreover, breeding system components, such as the number of times breeders mate and with whom, modulate how genes flow through populations (Blanco et al. 2021; Nichols et al. 2022; Ross 2001). Knowledge of breeding systems thus can help in identifying the potential for genetic conflict while also shedding light on social and dispersal biology (Blanco et al. 2021; Chapuisat and Keller 1999; Foster and Ratnieks 2001; Nichols et al. 2022; Pamilo et al. 1997; Ratnieks et al. 2006; Ross 2001). The basic conceptual foundations linking components of the breeding system (Table 1) to group composition and genetic structure were outlined decades ago by Wade (1985), Queller (1993), Ross (1993, 2001), and Kümmerli and Keller (2007a, 2007b).

**TABLE 1:** Major components of the breeding systems of social animals^a^.

Number of breeders and reproductive skew in the group
*Q*: number of female breeders (reproductive queens)^b^ in social group (colony)
$Q_{\bar{x}}$: arithmetic mean number of queens; *Q*_h_: harmonic mean number; *Q*_e_: genetically effective number
*M*: number of male breeders (mates per female breeder or queen)
$M_{\bar{x}}$: arithmetic mean number of mates; *M*_h_: harmonic mean number; *M*_e_: genetically effective number
*var*_q_: variance in queen reproductive shares (maternity skew within a colony)^c^
*var*_m_: variance in male reproductive shares (paternity skew in progeny of single queen) ^c^
Breeder relatedness^d^
*F*_IS_: inbreeding coefficient (*r*_(Fis)_: relatedness of mates)
*r*_q_: relatedness of colony‐mate female breeders (queens)
*r*_m1_: relatedness of male breeders that are mates of different queens in the same colony
*r*_m2_: relatedness of male breeders that are mates of the same queen
*r*_qm_: relatedness of different‐sex breeders in the same colony that are not mates
Progeny relatedness
*r*_p_: relatedness of female progeny in a colony
*r*_p(w)_: relatedness of worker daughters; *r*_p(g)_: relatedness of gyne daughters; *r*_p(ww)_: relatedness of worker daughters to worker (nurse) daughters; *r*_p(gw)_: relatedness of gyne daughters to worker (nurse) daughters

*Note:* *r*_sm_, *r*_dm_, *r*_sp_, *r*_dp_: relatedness of female progeny belonging to the same matriline, different matrilines, the same patriline, and different patrilines, respectively.

^a^ Modified from Ross (1993, 2001); note that matrilines and patrilines are equivalent when female breeders (queens) are monandrous, in which case we refer to them by default as matrilines.

^b^ Terms applied largely to social insects are underlined at first mention. Reproductive queens‐sexually mature adult queens that, in ants, have shed their wings and activated their ovaries; typically, most or all of these queens are mated; colony‐a term most commonly used to refer collectively to the members of an insect society; worker‐a more or less obligately sterile member of a social insect colony; fire ant workers are permanently sterile females lacking ovaries and a spermatheca (Hoffmann et al. 2024); gyne‐a “sexual” female in a eusocial hymenopteran colony, usually understood to exclude reproductive queens.

^c^ Many different estimators of reproductive skew exist (see Ross et al. 2020).

^d^ Many different estimators of genetic relatedness exist (see Goudet et al. 2018; Huang et al. 2014, 2015).

Features of breeding system components vary widely across animal taxa. The number of breeders in a group ranges from all sexually mature individuals (e.g., domiciliary cockroaches [Laurent‐Salazar et al. 2019]), to subsets of individuals (e.g., African wild dogs [Ben Mocha et al. 2023]), to single individuals of both sexes (e.g., naked mole rats [Buffenstein et al. 2021]). Closely connected to the number of breeders is the magnitude of reproductive skew, how evenly reproduction is shared among potentially fertile colony inhabitants. Skew reaches an extreme in eusocial animals with effectively one breeder (e.g., naked mole rats), where other group members typically support the breeder as helpers or workers. Relatedness among breeders also varies greatly, from unrelated (e.g., African apes [Clutton‐Brock 2021]) to highly related (e.g., meerkats [MacLeod et al. 2013]) to clonal groupmate breeders (e.g., some tropical fire ants [Lacy et al. 2019]). Despite extensive research across the diversity of social taxa, few studies offer comprehensive accounts of the genetic causes and consequences of variation in breeding system components in focal species (but see Azevedo‐Silva et al. 2020; Clutton‐Brock 2021; De Pletincx and Aron 2022; Griesser et al. 2017; Haag‐Liautard et al. 2008; Komdeur et al. 2016; Thurin et al. 2011).

**FIGURE 1:**
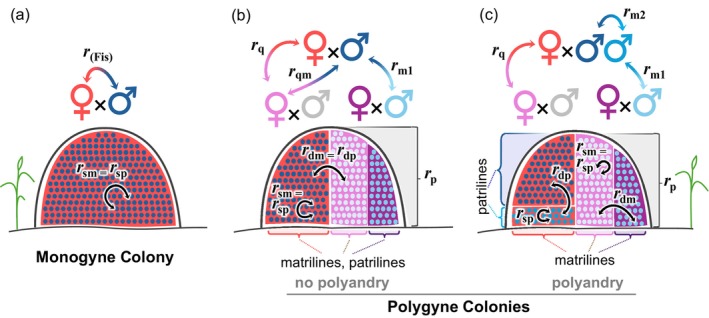
*Solenopsis invicta* breeding systems. Depictions of components of the breeding systems in (a) monogyne and (b, c) polygyne *S. invicta* . Monogyne queens are monandrous (a) and inbreeding evidently is absent (Ross et al. 1999; Ross and Fletcher 1985a; Ross and Shoemaker 1997; Shoemaker et al. 2006). Polygyne queens are usually monandrous (b) but occasionally polyandrous (c) (Fritz et al. 2006; Lawson et al. 2012; Ross and Shoemaker 2018); inbreeding is reportedly absent in this form as well (Ross and Fletcher 1985a; Shoemaker et al. 2006; Ross et al. 1999). Cartoon nests represent the genetic composition of the female progeny of reproductive queens inhabiting the nests; foreground dots represent paternal genetic contributions, and background coloring represents maternal genetic contributions. See Table 1 for meanings of abbreviations. Modified after Ross (2001).

The red imported fire ant, *Solenopsis invicta* , is native to South America (Ascunce et al. 2011) but over the past century has become established across an increasingly cosmopolitan invasive range (Menchetti et al. 2023; Sakamoto and Goka 2021). Its pest status in newly colonized areas is responsible for it becoming the second most researched social insect after the honey bee over the past half‐century.^1^ Considerable effort has focused on the two distinct social forms found in the species (Ross 1993, 2001; Ross and Fletcher 1985a; Tschinkel 2006). In the southern U.S., where they have been best characterized, the two forms differ not only in the most fundamental component of the breeding system—the number of reproductive individuals per colony—but also in other elements of the breeding system, as well as key life history traits such as queen longevity and mode of colony reproduction (Gotzek and Ross 2007; Ross 1988, 1993; Tschinkel 2006). In the “monogyne” form, colonies have a single queen and a simple family structure (Figure 1a; Ross and Fletcher 1985a). In contrast, the “polygyne” form features multiple cohabiting reproductive queens (hereafter, simply “queens”; we refer to all other sexualized females, including sexualized pupae, as “gynes”); the breeding system of this form thus is more complex, with variation in the number of breeders and reproductive skew important elements that are not relevant to the monogyne form (Figure 1b,c; Ross 1993).

Queen number polymorphism in fire ants is of special interest because it has been shown to be controlled genetically by a large inversion‐based, nonrecombining region on the q arm of chromosome 16 termed the *Sb* supergene. The *Sb* element is absent in the monogyne form, but is carried in the polygyne form by all queens (which are heterozygous for the *Sb* haplotype and its counterpart, the *SB* haplotype) and by most workers (which can be heterozygous *Sb*/*SB* or homozygous for either *Sb* or *SB*) (Helleu et al. 2022; Ross 1997; Yan et al. 2020). This supergene selfishly perpetuates itself in violation of Mendelian expectations, first observed as the destruction of adult gynes that do not carry *Sb* primarily by workers that do (Keller and Ross 1998; Trible and Ross 2016). Recent studies have revealed that *Sb* also is associated with non‐Mendelian segregation ratios in embryos through some form of transmission ratio distortion via unknown mechanisms (Ross and Shoemaker 2018). Further biasing its transmission, female larvae carrying *Sb* are more likely to develop as gynes (rather than sterile workers) than larvae lacking *Sb* (Buechel et al. 2014), monogyne workers (all homozygous for *SB*) are induced to tolerate heterozygous queens when exposed to relatively few workers bearing *Sb* (Zeng et al. 2025), and *Sb* is over‐represented among fertile haploid males produced in polygyne colonies (Fritz et al. 2006; Hettesheimer et al. 2024; ant and other Hymenopteran males are haploid and develop from unfertilized eggs). The selfish behavior of *Sb* in multiple contexts naturally perturbs components of the breeding systems of *S. invicta* , yielding alternate social organizations and breeding systems that occur on the same single‐species “genetic background.” Focused dissection of this system can help clarify how genetic variation can produce distinct breeding systems, yielding insights into how they function and evolve.

For this report, we conducted an expansive longitudinal study of laboratory polygyne colonies to complete the evaluation of each breeding system component, including those difficult or impossible to measure in the wild^2^ (Table 1; Figure 1). We integrate the results with reanalyzed data from previous datasets, with particular scrutiny given to the complex interrelationships between the breeding systems and the selfish *Sb* social supergene. One important set of results of the laboratory experiment concerns the temporal dynamics of breeding system elements, such as the stability of reproductive skew in both queens and males. We also present new, formal analyses of the effects of male supergene haplotype on the mating system. Most fundamentally, the study provides a template for research on the breeding systems of focal animal species that display some form of social reproduction.

## 2 Materials and Methods

### 2.1 Samples

**FIGURE 2:**
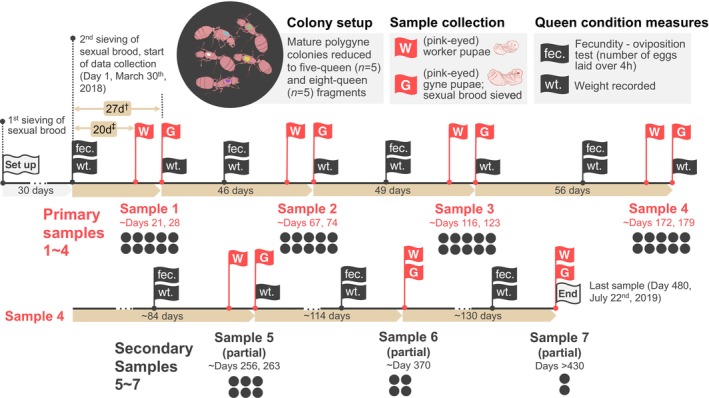
Design of the experiment that generated the main dataset (*GA‐2023*) used in this report. The experiment started immediately after the second round of removal of sexual brood by sieving (Day 1). The fecundity and weight of the marked reproductive queens were recorded at the points indicated by black flags. Red flags indicate when worker and gyne pupae were sampled. All sexual brood and adults were removed from colonies following completion of each gyne sample collection to prevent carry‐over to the subsequent sampling point. Black dots indicate the number of colonies at each sampling point. ‡ − time between oviposition tests (and weigh‐in) and sampling of worker pupae (20d, approximate age of young worker pupae); † − time between oviposition tests (and weigh‐in) and sampling of gyne pupae (27 days, approximate age of young gyne pupae).

Most of the data we analyze in this report comes from a long‐term laboratory study of the breeders and their female offspring in 10 polygyne *S. invicta* colonies collected in northern Georgia, U.S. (the *GA‐2023* dataset; Figure 2; Method S1; Video S1). Briefly, we removed all winged sexuals (gynes and males) from each colony and then reduced the number of wingless (reproductive) queens to either five or eight. We paint‐marked these queens to identify them over the length of the experiment and assessed the reproductive output of each by sampling offspring pupae repeatedly over periods ranging from 7 months to over a year. Four “primary” samples (Sampling Points 1–4) were collected for all 10 colonies, and six colonies were sampled an additional 1–3 times (Sampling Points 5–7).

We assessed the fertility of queens by weighing them and quantifying their oviposition rates 20 days before sampling worker pupae and 27 days before sampling gyne pupae. Based on reported developmental periods (O'Neal and Markin 1975), the sampled offspring were derived from eggs laid around the time of the fertility tests. To assess parentage shares, at every sampling point we collected for genotyping 95 (5‐queen colonies) or 190 (8‐queen colonies) young pupae (nonmelanized cuticle, pink‐eyes) of each caste (worker, gyne). In rare cases, when a sufficient number of young gyne pupae were not available, we opportunistically collected more mature pupae or teneral adult gynes. After each sampling point, we removed any remaining sexual brood and adults from each colony. Because of this and other unnatural manipulations, conclusions regarding queen reproduction should be tempered until complementary data are available from wild colonies. On the other hand, most breeding system features pertaining to males are expected to be generalizable, given that these males are represented by sperm in a queen's spermatheca, which should not be subject to effects of manipulation in the laboratory environment.

We extracted genomic DNA and performed microsatellite genotyping on all sampled female progeny, as well as most reproductive queens and their mates. Altogether, we scored microsatellite genotypes for 6195 worker pupae, 3498 gyne pupae or teneral adults, and 62 queens. We also determined the haplotypes of 64 male mates of these queens, using sperm from the queen spermatheca for direct haplotype determination and/or inferring the haplotype from the reconstructed matrilines.

The 10 colonies were sampled over periods ranging from 27 to 69 weeks. To evaluate approximate “lifetime reproductive success” of queens, we focused on five colonies for which all queens survived through at least the primary (first four) sampling periods. We view the results from this subset as proxies for queen lifetime reproductive success in the wild because our primary sampling periods covered the period from April to September, when polygyne colonies in northern Georgia produce most of their annual yield of new gynes (Vargo and Fletcher 1987). Also, polygyne queen longevity is likely less than 3 years based on earlier longitudinal studies (Tschinkel 2006; also Figure 4b), so polygyne queens likely participate in only 1–3 seasonal bouts of reproduction over their lifetime in the wild.

We supplemented results from the *GA‐2023* dataset by analyzing a second dataset derived from an earlier study of transmission of the *Sb* supergene in polygyne *S. invicta* (the *GA‐2018* dataset; see Ross and Shoemaker 2018, Method S2, Video S2). Briefly, mated queens from each of 12 colonies were isolated for 12 h for collection of eggs they laid, and DNA from ~35 of the resulting 48‐h old embryos from each queen was extracted for microsatellite genotyping, yielding genotypes of 3346 diploid offspring embryos, as well as genotypes of 101 mother queens and 105 of these mother queens' male mates obtained as in the *GA‐2023* study.

### 2.2 Genotype Data Generation

Individuals sampled for these two datasets were genotyped at 13 microsatellite loci (see Method S3; Ascunce et al. 2009; Ross and Shoemaker 2018). Eleven of these 13 microsatellite markers are distributed among six of the 15 non‐supergene chromosomes of *S. invicta* (Table S1), while the other two are located either within the Chr16 *Sb* haplotype associated with polygyny (*C294*) or on the other arm of Chr16 (*i‐126*). The 11 markers not on Chr16 are genetically unlinked, with high levels of recombination between almost every pair of loci (two pairs that display moderately reduced recombination [*C27* and *C536*, *i‐109* and *Sunrise*] occur on the same chromosomes). The complete panel of markers is sufficiently polymorphic (Table S1) that the polyandry nondetection error (Pedersen and Boomsma 1999) is estimated at 5.3 × 10^−8^.

### 2.3 Sources of Additional Genetic Data

We reanalyzed genetic data from earlier studies, including (i) allozyme genotypes for females from both social forms in northern Georgia (Ross 1993; Ross and Shoemaker 1997) and a polygyne population in central Texas (Ross et al. 1996), (ii) both allozyme and microsatellite genotypes from six monogyne populations located across the southern U.S. (Shoemaker et al. 2006), (iii) microsatellite genotypes from northern Georgia monogyne populations (Ascunce et al. 2011; Fisher 2013), and (iv) supergene genotypes and haplotypes for queens and their mates from Florida polygyne and monogyne populations and a Mississippi polygyne population (Lawson et al. 2012) (Method S4). Our intent was to integrate available information from diverse sources to accurately characterize the complex breeding systems of a species over a large part of its range.

### 2.4 Estimation of Genetic Relatedness

We estimated the average genetic relatedness (*r*) for pairs of individuals from single colonies. For microsatellite‐based *r* estimates, the reference population consisted of 172 queens and 166 of their haploid mates from the 22 colonies in the *GA‐2018* and *GA‐2023* datasets (see Method S5). Allozyme‐based estimates included all genotyped individuals from the focal population as the reference.

Many estimators are available to calculate relatedness between pairs of individuals. Because the performance of each varies depending on features of the data and actual kinship levels (Huang et al. 2014, 2015; Städele and Vigilant 2016; Taylor 2015; Wang 2017), we first evaluated the performance of 19 popular estimators using simulations on our two marker types (Method S5). We chose the Huang et al. (2014) method‐of‐moments estimator for all relatedness estimates because of its consistently low bias and sampling variance in the simulations and its accuracy in test groups of known pedigree relatedness (Figures S1 and S2).

### 2.5 Breeder Numbers and Reproductive Skew

We summarized the number of female breeders (queens) per nest in the wild by searching the literature for queen counts based on thorough nest excavations (Method S6). Simple averages of direct counts of breeders (or of matrilines/patrilines) do not consider the effects on colony genetic composition of variation in breeder numbers, turnover of breeders, or reproductive skew (unequal partitioning of maternity between nestmate queens or of paternity between the male mates of single queens). Thus, two metrics of breeder numbers, in addition to simple arithmetic means of counts ($Q_{\bar{x}}$, $M_{\bar{x}}$), were estimated (Table 1): the harmonic mean (*Q*_h_, *M*_h_), which accounts for variation in breeder number per group, and the genetically effective number (*Q*_e_, *M*_e_), which further takes into account reproductive skew (Method S6, Video S3).

We directly estimated *Q*_e_ and *M*_e_ from the variance in parentage following offspring parentage assignments (see Method S3) using equations (4.15) and (4.20) in Crozier and Pamilo (1996), or equation (16) in Nielsen et al. (2003); results from the two estimators were similar (Figure S18), so we used the former methods in most cases. For a few applications, we indirectly estimated the effective number of breeders from progeny nestmate relatedness using the relevant equations in Ross (2001). The congruence of the direct and indirect approaches to estimating effective breeder number is shown in Figure S3 (also Method S6).

To calculate maternity and paternity skew, we used the *M*‐index of Ross et al. (2020). For the *GA‐2023* dataset, the *M*‐index was estimated for each sampling point as well as over the course of the experiment, accounting for the different residency times of queens (Method S7). Because the temporal samples for each mother queen are not independent, the magnitude of skew for the different castes was compared using a resampling bootstrap difference routine. This approach involved repeated random sampling of a single pupa per colony or matriline for each sampling point.

### 2.6 Characteristics of Polyandrous Queens

Polyandry is uncommon in *S. invicta* but sufficiently prevalent in some polygyne populations to analyze paternity skew and other features of the breeding and reproductive systems. To test a previous hypothesis that only queens that mate with males bearing the *Sb* haplotype are likely to remate (Lawson et al. 2012), we used our data on queen mating frequencies and mate haplotypes to estimate remating probabilities conditioned on the first and second mates' supergene haplotypes (Method S8). We developed a simulation model to estimate these probabilities separately in two populations (Florida, Georgia). The most likely model estimates were those obtained when the estimated data best fit the empirical data.

To identify features that distinguish polyandrous from monandrous queens, we analyzed data collected on queen condition and progeny matriline membership over time (*GA‐2023* dataset). We examined the association of multiple mating with a queen's production of worker and gyne offspring, as well as with her weight and fecundity (Figure S5; Method S9).

## 3 Results

Monogyne colonies of *S. invicta* by definition have a single female breeder (queen) reported to be mated almost inevitably to a single unrelated male (Figure 1a; Table S2), meaning each colony is a simple (albeit enormous) family. Because males are haploid and inbreeding appears unlikely, female progeny are full sisters with expected relatedness *r*_p_ = 0.750. We estimate *r*_p_ = 0.730 (bootstrap 95% CI = 0.687–0.770), using the Huang et al. (2014) estimator on newly aggregated genotypic data from colonies in northern Georgia (Method S10; also Ross and Fletcher 1985a).

**FIGURE 3:**
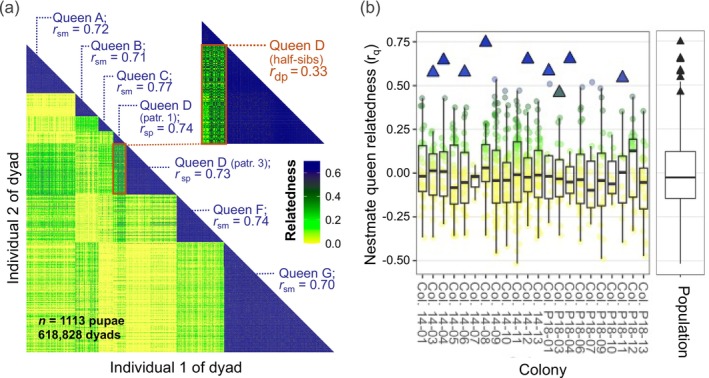
Relatedness in polygyne *S. invicta* nests estimated from genotypes at 11 microsatellite markers. (a) Heatmap of pairwise relatedness (*r*_p_) for all dyads of genotyped progeny females (castes pooled) from a single colony with six mated queens (*GA‐2023* dataset, Colony P18‐03, four primary samples pooled). Five of the dark blue triangles in the complete heatmap correspond to matrilines containing full‐sister dyads for the five monandrous queens; the remaining two blue triangles correspond to the two patrilines containing full‐sister dyads for the twice‐mated Queen D. Average values of *r*_sm_ for the first five queens and *r*_sp_ for the sixth are close to the expected pedigree value of 0.75. The estimated value of *r*_dp_ between the half‐sister offspring of Queen D (0.33; red rectangle) is close to the expected pedigree value of 0.25. (b) Distributions of pairwise relatedness values for 174 nestmate reproductive queens (*r*_q_) sampled from 22 colonies (*GA‐2018* and *GA‐2023* datasets; mean of 7.9 queens per colony; *n* = 678 dyads; mean *r*_q_ = −0.007). Median relatedness values for each colony are shown with a horizontal line in the box. The few outliers identified from the population boxplot (using Tukey's Rule) are nine dyads (blue triangles) that may represent exceptional pairs of closely related nestmate queens.

Polygyne colonies have a far more complex kin structure due to the presence of multiple queens, some of which mate multiply (Figure 1b,c). Thus, colonies feature a mix of full‐sister families and half‐sister subfamilies, presumably with low relatedness between families but unknown relatedness between subfamilies. The relatedness patterns for female offspring in such a colony are visible at a glance in an *r*_p_ heat map derived from data generated here (Figure 3a). Graphs showing the dynamic matriline composition of all 10 *GA‐2023* colonies are shown in Figure S5.

### 3.1 Number of Breeders

#### 3.1.1 Number of Female Breeders

**FIGURE 4:**
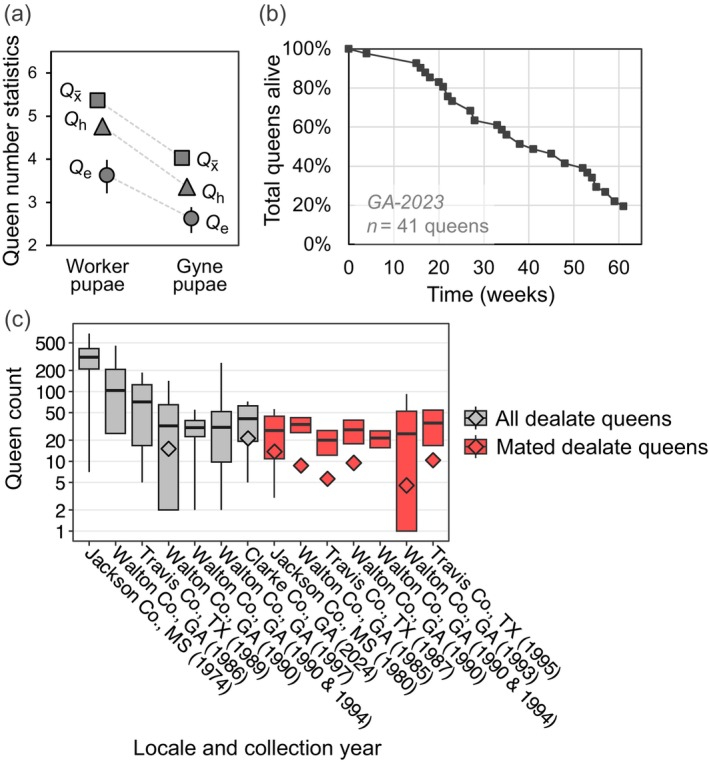
Overview of nestmate queen number and queen longevity in polygyne *S. invicta* . (a) Three summary population‐level metrics of colony queen number in 10 laboratory colonies for which queen number was known and maternity apportionment of daughter pupae was determined separately for each caste (*GA‐2023* dataset). Values of the three metrics (*Q*_e_ estimated directly) are based on 5437 worker pupae and 3034 gyne pupae; error bars represent the 95% confidence intervals based on 5000 bootstrap replicates. (b) Survivorship curve for polygyne queens in the *GA‐2023* dataset. (c) Published counts of queens in polygyne nests in the wild in the southern U.S. The thick lines in the boxes represent arithmetic means, while the diamonds represent harmonic means when calculable (see Table S3 for more information and references). Each box represents the range from one standard deviation below to one standard deviation above the arithmetic mean. Whiskers, if present, extend to the minimum and maximum values for that dataset. Note the nonlinear scale for the *y*‐axis.

Early studies in the invasive range of *S. invicta* recovered wingless (presumably egg‐laying) queens by methodically excavating polygyne nests. Enormous variation in queen number is evident in these counts, which range from two to several hundred queens per nest, with averages at a site ranging from 30 to 300 (Figure 4c; Table S3). The number of nestmate queens is dynamic on a scale of months to years, as revealed by longitudinal studies in the field (Porter 1991; Vargo and Porter 1989) and laboratory (Ross 1988; Figure S5). One possible consequence of fluctuations in the number of breeders is that the parents of some progeny in a nest are no longer present. Conversely, some queens present in polygyne nests are not functional breeders—in the U.S. range, unmated queens make up 10%–50% of the wingless queens in a nest (Table S3) but produce only very few viable eggs (Vargo and Ross 1989), which necessarily develop as males via arrhenotokous parthenogenesis (as is typical for unfertilized eggs in Hymenoptera).

A different approach to estimating the number of female breeders entails genotyping progeny produced in colonies. With enough sufficiently polymorphic loci, counts of matrilines (queens) can be obtained by parentage assignment (see Method S3), and values of three metrics of queen number, the arithmetic mean ($Q_{\bar{x}}$), harmonic mean (*Q*_h_), and genetically effective mean (*Q*_e_) numbers of queens per nest can be estimated (see Method S6). Variation in actual queen (matriline) counts among colonies means *Q*_h_ < $Q_{\bar{x}}$ because the former gives greater weight to lower values in a series, that is, to nests with fewer queens (Method S6). Variation in shares of parentage (skew) means that *Q*_e_ < *Q*_h_ because *Q*_e_ also accounts for the increase in proportion of higher‐value (e.g., within‐family) relative to lower‐value (e.g., between‐family) dyad‐relatedness values accompanying an increase in skew (effect similar to lowering queen number; Video S3). Our analyses of progeny genotypes in the *GA‐2023* dataset reveal the expected ordering of values, *Q*_e_ < *Q*_h_ < $Q_{\bar{x}}$ (Figure 4a). The same pattern can be seen from our reanalysis of data from polygyne nests in the field (Table S5).

Estimating these metrics separately for the progeny of each caste in the *GA‐2023* colonies again revealed this ordering of values in both castes, but with consistently lower estimates across the three metrics for gyne than worker offspring (Figure 4a). This was true as well for *Q*_e_ newly estimated for field colonies (Ross 1993; Table S6). These results suggest that there often are fewer mothers of gynes than of workers within colonies (Bargum and Sundström 2007); that is, successful production of sexualized daughters is less common among queens than production of sterile worker daughters. Indeed, out of 30 samples from the *GA‐2023* dataset in which at least 50 pupae of each caste were collected, nine samples (18%) featured fewer detected gyne than worker matrilines, whereas only two (4%) showed the opposite pattern (the numbers of matrilines for each caste were equal for the remaining samples).

#### 3.1.2 Number of Male Breeders

**FIGURE 5:**
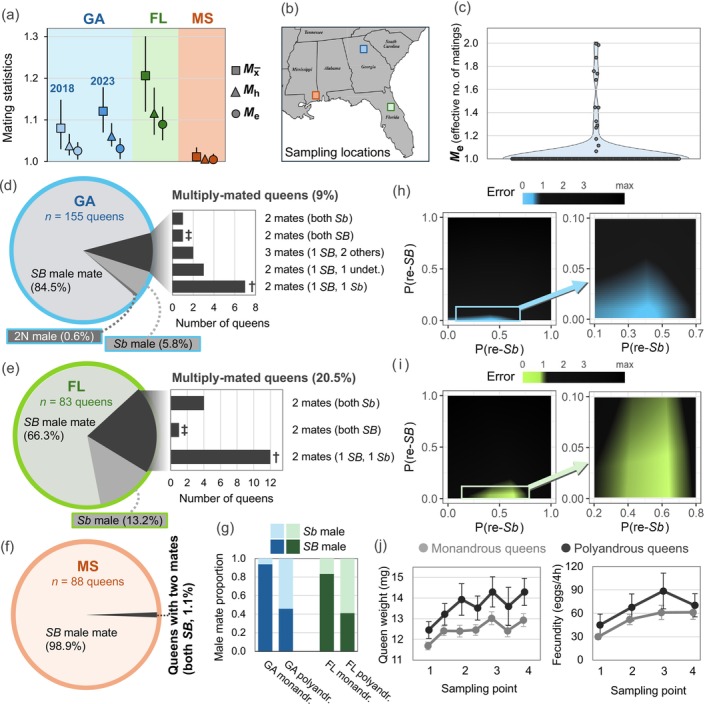
Male breeders and polyandry in polygyne *S. invicta* . (a) Three summary population‐level metrics for numbers of queen matings in three invasive (U.S.) polygyne populations, with sampling locales depicted in (b); vertical lines represent 95% CIs based on 5000 bootstrap resamplings. Data for Georgia (GA) are from the *GA‐2018* and *GA‐2023* datasets. Newly analyzed data for Florida (FL) and Mississippi (MS) are from Lawson et al. (2012). Effective numbers of mates per queen (*M*_e_) for all populations were estimated directly using equation (4.15) in Crozier and Pamilo (1996) and assuming values for paternity variance obtained from the present study. (c) Distribution of *M*_e_ values estimated directly for 156 Georgia polygyne reproductive queens from *N* = 22 colonies; *n* = 11,795 diploid offspring assigned to patrilines. (d–f) Proportions of queens in GA, FL, and MS populations that mated with a single male (monandry, gray slices) or multiple males (polyandry, black slice). The single mates of monandrous queens are further distinguished by Chr16 supergene haplotype (or, in one case, ploidy). Polyandrous queens in the GA and FL populations are subdivided by the supergene haplotypes of each queen's mates in the bar charts; daggers and double‐daggers indicate male mate combinations that are significantly overrepresented or underrepresented, respectively, based on the frequencies expected given estimated male haplotype frequencies in the local mating swarms. (g) Comparisons of male mate supergene haplotype proportions for monandrous and polyandrous queens from the GA and FL populations. (h, i) Error heatmaps showing deviation of the estimated number of queen mating types from the observed number for hypothetical remating probabilities after a queen mates with a *Sb* (*x*‐axis) or *SB* (*y*‐axis) male in the GA (h) and FL (i) populations. Bright colors highlight regions with low error and thus high probability of the parameter values explaining the data. *p*_(re‐*Sb*)_—probability that a queen will remate with a second male if she first mates with a male bearing the *Sb* haplotype; *p*_(re‐*SB*)_—probability that a queen will remate if she first mates with a male bearing the *SB* haplotype. (j) Comparisons of weight and fecundity between monandrous and polyandrous reproductive queens (*GA‐2023* dataset; see also Figure S8). Error bars represent ±1 SE.

Counting the numbers of mates per queen requires genotyping, either of dissected spermathecal contents or of progeny collected after queens lay eggs in isolation; alternatively, assigning genotyped progeny to matrilines and patrilines in multi‐queen colonies, as we did in the *GA‐2023* experiment, can be used to obtain such counts. Polygyne queens previously were reported to mate with a single male, with some exceptions (Fritz et al. 2006; Lawson et al. 2012; Ross and Fletcher 1985a; Ross and Shoemaker 2018). To explore this variation further, we compiled data on counts of mates per queen, adding data from an earlier study of polygyne populations in Florida and Mississippi (Lawson et al. 2012) to our data from Georgia (Figure 5a–f). The arithmetic mean number of queen matings ($M_{\bar{x}}$) ranged from 1.01 to 1.21 in the three populations, while the genetically effective number of mates (*M*_*e*_) never rose above 1.09. The ordering of values of $M_{\bar{x}}$, *M*_h_, and *M*_e_ invariably matches that for the analogous metrics for queens (Figure 5a). Unsurprisingly, most of the 155 mated queens from Georgia were singly mated—14 (9.0%) had mated multiply (Figure 5d), twelve with two males and two with three males. In the instances of three matings, one male fathered only a tiny fraction of the queen's offspring (0.54%, 3.03%).

Haploid *S. invicta* males can have either the *SB* or *Sb* supergene haplotype, and the frequency of adult males in polygyne nests is considerably biased toward *Sb* males (≈20:80 [*SB*:*Sb*]; Table 3). Perhaps surprisingly, given this imbalance, most monandrous queens from our Georgia polygyne colonies mated with a male bearing *SB* (see also Ross 1997), with only a small proportion (6%) having mated with an *Sb* male (Figure 5d). In contrast, most polyandrous queens (91%) had at least one *Sb* mate. An identical pattern was observed in Florida (Figure 5e).

One explanation for this remarkable association of queen mating frequency with the supergene haplotype of her mate(s) is Lawson et al.'s (2012) hypothesis that polygyne *S. invicta* queens generally mate a second time only if they first mated with an *Sb* male (see Table 3). If true, the number of twice‐mated queens that mated with one male bearing each supergene haplotype should be greater than expected based on presumed male haplotype frequencies in the local mating swarms (assumed to be identical to the proportion of all matings attributable to males of each haplotype). Indeed, matings of polyandrous queens with one male of each haplotype occurred significantly more frequently (*p* = 0.01; resampling randomization test), and matings of queens with two *SB* males less frequently (*p* = 0.04), than expected (Figure 5d,e). We thus conducted additional analyses using simulations (Method S8), which estimated the probability of a queen remating at 30%–60% if she mated with an *Sb* male and < 1% after mating with an *SB* male both in the Georgia and Florida populations (Figure 5h,i).

Polyandrous queens in our *GA‐2023* dataset did not differ from monandrous nestmate queens in their relative contribution to the colony's total output of worker or gyne offspring (1‐tail bootstrap difference tests, 5000 iterations, both *p* > 0.25; Method S9). Intriguingly, however, polyandrous queens weighed significantly more and had significantly higher fecundity than their monandrous counterparts (Figure 5j; resampling bootstrap difference tests; *p* < 0.001 and *p* = 0.008, respectively; weight and fecundity are associated in polygyne reproductive queens [Figure S4]).

This congruence of polyandry with queens exhibiting high fertility after transitioning to egg‐laying may be caused by intrinsic physiological differences between polyandrous and monandrous queens. Alternatively, because virtually all polyandrous queens mated with at least one *Sb* male while monandrous queens overwhelmingly mated with an *SB* male, it is conceivable that the ejaculates of *Sb* males contain substance(s) facilitating long‐term elevation of queen weight and fecundity (e.g., South and Lewis 2011). Based on our observations, the latter seems unlikely. The two monandrous queens in the *GA‐2023* dataset with a *Sb* mate resembled other monandrous queens (Figure S8c,d). Also, the sole polyandrous queen that mated with two *Sb* males was similar to all other polyandrous queens in her weight and fecundity, as was the sole thrice‐mated queen (one *SB* and two *Sb* mates). These data are consistent with the number of matings, rather than the supergene haplotype(s) of male mates, being the major factor influencing queen fertility later in life. Although it is possible that queens that intrinsically become more fecund simply are more inclined to mate multiply, most twice‐mated queens would then be expected to have two *SB* mates, in stark contrast to the pattern observed (Figure 5d,e).

### 3.2 Reproductive Skew

Reproductive skew is the unevenness of reproductive output among same‐sex breeders in a group. Although immaterial to the monogyne form of *S. invicta* , reproductive skew in both sexes likely is important in the polygyne breeding system.

#### 3.2.1 Maternity Skew

**FIGURE 6:**
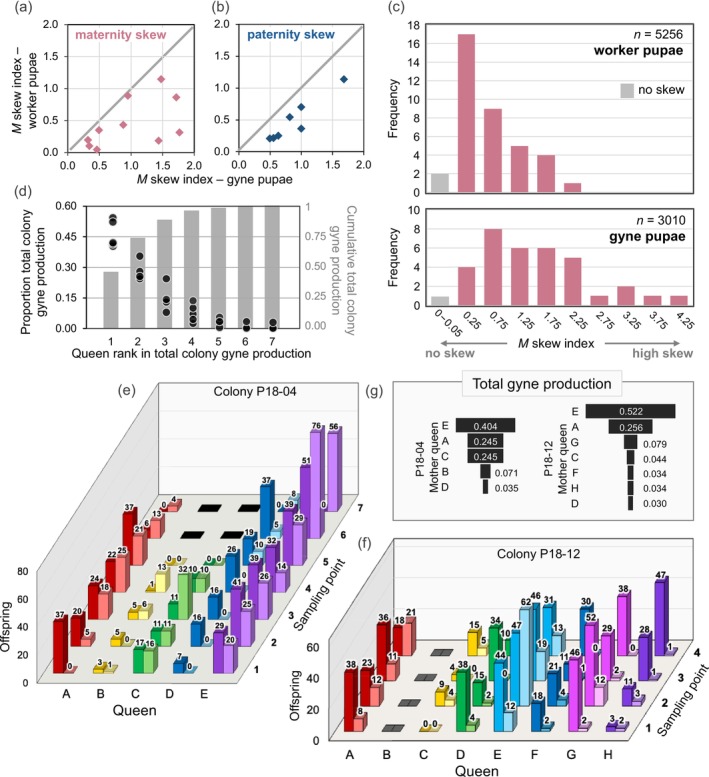
Reproductive skew in polygyne *S. invicta* . (a) Maternity skew for gyne progeny plotted against skew for worker progeny from the same colony over the four primary samples (*GA‐2023* dataset). Skew is measured with the *M*‐index, which accounts for differences in individual queen residency in a colony. If the magnitude of maternity skew is similar between the castes, the points will fall along the dashed line. (b) Same as (a) but for paternity skew. (c) Distributions of *M*‐index values for maternity skew in 10 colonies at the four primary sampling points; distributions for pupae of the two castes are shown separately. Numbers on the *x*‐axis are bin midpoints except for the first column. (d) Proportion of total colony gyne production by each queen in five colonies that experienced no queen mortality through the four primary sampling points (five points for P18‐04). The dots show each queen's share of her colony's gyne production over all sample points, while the columns show the average cumulative production of a colony moving from more to less dominant queens. Note the different scales on the two *y*‐axes. (e, f) Numbers of female pupal offspring of each caste produced by each queen at each sampling point in two representative colonies in the *GA‐2023* dataset; the left column of a pair (darker shading) indicates worker pupae while the right column (lighter shading) indicates gyne pupae. Black tiles denote dead queens, and gray tiles denote a live unmated queen. (g) Proportion of total colony gyne production by each queen over the four primary sampling points (five points for P18‐04) in the same two colonies as in (e, f).

We found significant maternity skew in our *GA‐2023* laboratory colonies (Figure 6a,c). When the *M*‐index of skew was calculated separately for each caste and colony at each of the four primary sampling points, the 95% CIs for each of the resulting eight sets of values never included zero, the value indicating parity in reproduction (1000 bootstrap samples, minimum CI for workers: 0.366–0.911; gynes: 0.963–1.737; Method S7). Moreover, for 72 of the 75 individual samples that included both gyne and worker progeny, skew was significantly greater than that expected due to sampling error alone (simulation analyses; Method S7); the three exceptions all involved worker progeny.

Previous work has directly detected substantial maternity skew in laboratory nests of polygyne *S. invicta* (Ross 1988). In the field, average counts of queens per nest typically exceeded the effective numbers estimated from nestmate progeny relatedness (Ross 1993) (Table S5), likely at least partly the result of unequal sharing of maternity, although the presence of recent immigrant queens cannot be ruled out as a factor (see also Fournier et al. 2002; Hannonen et al. 2004; Orr et al. 2024; Ross 2001; Trontti et al. 2005).

*M*‐index values were significantly higher for gyne than worker offspring in 80% of samples in the *GA‐2023* dataset (resampling bootstrap difference test across all samples, *p* = 0.008; Method S7; also Figure 6a,c). This finding aligns with the higher relatedness values obtained for nestmate gyne than worker pupae at 83% of samples (Figure S9) and is consistent with the lower estimate of *Q*_e_ for gynes than workers (Figure 4a).

Do these data reflect the presence of “winner” queens in long‐term competition for reproductive success? Five colonies in the *GA‐2023* dataset are especially relevant to this question (Figure 6d–g; Figure S10) because all queens lived through at least the four primary sampling periods, thus living one entire season of gyne production of the few seasons that polygyne *S. invicta* queens survive in nature. The first‐ and second‐ranked gyne producers together produced on average about three‐quarters of all gynes reared by the colony over that period (Figure 6d), with the top‐ranked queen averaging one‐half (mean = 46%; Figure S10). Clearly, there is a subset of queens that have inordinately high reproductive success over meaningful timespans (Figure 6g).

Such dominance by one or two prospective “winner” queens generally was not consistent across temporal samples, nor was it necessarily accompanied by correspondingly low production of worker daughters (Figure 6e,f; Figure S5). On the other hand, queens with consistently high production of workers but low production of gynes, prospective “losers,” were quite common (Figure 6e,f; Figure S5). Patterns in maternity skew and characteristics of queens with exceptionally high or low production of gyne daughters are important issues that will be evaluated in detail elsewhere.

A potentially important factor contributing to maternity skew in these ants is the *Sb* supergene, which is known to be a recessive deleterious variant in workers and a recessive lethal in gynes (Gotzek and Ross 2007; Hallar et al. 2007). Because all queens are heterozygotes, the Mendelian expectation is that *Sb* homozygotes represent 50% of daughters of queens that had only *Sb* mates. If potentially up to half of their female offspring are lost before becoming mature adults, such queens likely are at a significant reproductive disadvantage. Three queens in the *GA‐2023* dataset mated only with *Sb* males (P18‐01E, −13A, −13G), and these queens consistently produced relatively few daughters of either caste (Figure S5). Low reproductive success from this cause must be rare, however, because the low mating and fertilization success of *Sb* males (below) strongly limits the opportunity for such selection to alter genotype frequencies.

#### 3.2.2 Paternity Skew

Paternity skew was conspicuous for the seven polyandrous queens in the *GA‐2023* dataset (Figure S11). As with maternity skew, there were no instances of zero or negative *M*‐index estimates for paternity skew (Figure S19). Paternity skew values for gyne offspring consistently exceeded those for worker offspring (Figure 6b; Table 3), raising the possibility of caste‐specific differential paternity skew.

#### 3.2.3 Effects of Male Supergene Haplotype on Paternity Skew

**FIGURE 7:**
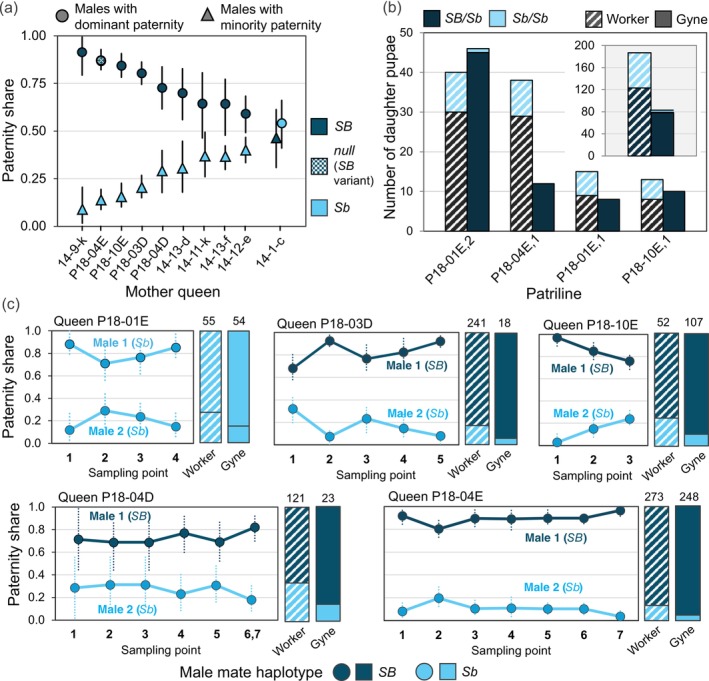
Paternity skew and detrimental effects of *Sb* on male reproductive success in polygyne *S. invicta* . (a) Paternity apportionment among female progeny for the two mates of each of 10 polyandrous queens confirmed to have mated with an *SB* male and an *Sb* male (lines represent 95% CIs from 1000 bootstraps of paternity shares). Pupal offspring (both castes) were studied for queens with the “P18” prefix, whereas diploid embryos were studied for queens with the “14” prefix. The binomial probability that an *SB* rather than *Sb* male would dominate paternity by chance in ≥ 9 of the 10 cases is 0.011. Note that the one case with the alternate pattern (Queen 14‐1‐c) also features the smallest (and only statistically nonsignificant) inequity in the two males' paternity shares. (b) Numbers of daughter pupae with each supergene genotype produced by four queens that mated with males with the *Sb* haplotype. The queens were polyandrous and only the *Sb*‐male patriline, identified by the *x*‐axis labels, is considered (both mates of Queen P18‐01E bore the *Sb* haplotype). Each patriline is expected to segregate the two genotypes in a 1:1 ratio in each caste. Only patrilines with eight or more gynes are included. Inset: Same data combined with genotype counts from four patrilines with < 8 gynes sampled. (c) Paternity apportionment over time for twice‐mated polyandrous queens (*GA‐2023* dataset; data for two polyandrous queens with only two progeny sampling points are not shown). The plots show patrilines of female pupae (castes combined), with the mate dominating paternity designated “Male 1” and the other mate “Male 2”; the supergene haplotypes of each are listed. For two of the queens (P18‐10E, P18‐03D), paternity shares varied significantly across samples (Fisher's exact test; both *p* < 0.05), though all temporal changes were modest. Points on the *x*‐axis are not scaled according to actual times between sample points. To the right of each of these plots are column graphs showing cumulative paternity apportionment across the entire experiment for each caste; sample size is above each column.

Because most polyandrous queens mate with one *Sb* and one *SB* male, and given the documented circumstances in which the *Sb* element selfishly manipulates reproduction in its favor (Keller and Ross 1998; Lawson et al. 2012; Buechel et al. 2014; Hettesheimer et al. 2024), we examined the possibility that the supergene haplotype of such queens' mates influences paternity skew. In contrast to the pattern expected if the *Sb* element behaves selfishly in this context, *Sb* fathers almost always produced fewer offspring in a matriline than their *SB* competitors, a fact evident in both embryos and pupae from the two current Georgia datasets (Figure 7a; Figures S6, S12 and S13). Among the 10 polyandrous queens confirmed to have mated with one male of each haplotype, there was only one instance in which the *Sb* male sired a higher proportion of offspring (55%) than the *SB* male, and this was the least extreme and only statistically nonsignificant instance of paternity skew (Figure 7a; 95% CIs from 1000 bootstraps of paternity shares).

This pattern of skew involving a dominant *SB* male and less successful *Sb* male was constant, with shares of paternity typically stable over long periods (Figure 7c; Figure S6). Remarkably, there was not a single instance of paternity dominance switching between the two mates of a queen for either offspring caste during the *GA‐2023* experiment (13–65 weeks).

The possibility that the magnitude of paternity skew is more extreme in the case of sexual than worker daughters (Section 3.2.2) is supported by examination of the matrilines descended from polyandrous queens (Figure S13). Among the seven such matrilines composed of two patrilines, each derived from a male of different haplotype, six exhibited higher paternity skew among gyne progeny than worker progeny, with this disparity always favoring the *SB* over the *Sb* father (1‐tail bootstrap difference tests, 1000 iterations; all *p* ≤ 0.05). The seventh matriline (P18‐01E), in which no significant difference in caste‐specific skew was evident, was also unique in that both fathers had haplotype *Sb* (Figure S13).

The difference in the extent of dominance of *SB* over *Sb* male paternity according to offspring caste may stem in part from the caste‐specific viability differences for *Sb* homozygotes described above. For eight patrilines featuring a heterozygous mother and *Sb* father, in which the progeny are expected to segregate *Sb/Sb* and *SB/Sb* genotypes in equal proportions, the overall proportion of homozygotes was only 0.35 for worker pupae and a mere 0.01 for gyne pupae (*n* = 190 and 77, respectively; both *p* < 0.0001;1‐tail randomization test for 1:1 ratio; Figure 7b), indicating strong selection against *Sb* homozygotes even before the pupal stage.

The almost complete loss of homozygous *Sb/Sb* gynes is necessarily restricted to patrilines headed by *Sb* males, which already are disadvantaged in their overall paternity success, presumably because they produce 32% fewer sperm than *SB* males (Table 3), which translates to corresponding deficiencies in fertilization success. *Sb* males thus appear to suffer compound penalties in their competition for paternity with *SB* mates of the same queen. Consistent with this hypothesis, among the total pupal progeny in the *GA‐2023* dataset, *Sb/Sb* homozygotes are significantly underrepresented among gynes but not workers relative to the frequency of the genotype in diploid embryos (data from the *GA‐2018* dataset; *p* < 0.001 and *p* = 0.121, respectively; resampling tests; Figure S14). A deficit of *Sb/Sb* homozygotes leads to the departure of gyne pupal genotypes from Hardy–Weinberg expectations, not seen for embryo or worker pupal genotypes. Notably, minority (*Sb*) males always sired fewer female offspring than predicted from the average proportion of *Sb* sperm stored in a polyandrous queen's spermatheca, but this pattern is accentuated in gyne pupae compared to worker pupae (Figure S15).

### 3.3 Breeder Relatedness

We confirmed that relatedness is negligible between breeders (single queen and her mate) in monogyne *S. invicta* colonies (*F*_IS_ < 0.05; Table S2). Relatedness between breeders in polygyne nests requires consideration of multiple classes of breeders in addition to mates (Table 1).

#### 3.3.1 Relatedness of Mates (Inbreeding)

For polygyne *S. invicta* , our analyses using the *GA‐2023* and *GA‐2018* datasets yielded an estimate of the inbreeding coefficient (*F*_IS_) statistically indistinguishable from zero (Table S4), with the average genetic relatedness between mates, *r*_(Fis)_, also estimated as zero (Table 2). Earlier studies reported *F*_IS_ values close to zero (e.g., Table S4) (Ross et al. 1987; Ross 1993; Ross and Fletcher 1985a; Shoemaker et al. 2006), but these often relied on few genetic markers and/or samples, and none estimated relatedness between mates.

**TABLE 2:** Relatedness values estimated for various classes of breeders and their female progeny in invasive polygyne *S. invicta* in the U.S.

	Mean *r* ^a^	95% confidence limits^b^	No. dyads
Breeders			
*r*_(Fis)_	−0.009	−0.029 to 0.011	169
*r*_q_	−0.007	−0.022 to 0.009	678
*r*_qm_	0.001	−0.009 to 0.011	1268
*r*_m1_	−0.003	−0.014 to 0.001	611
*r*_m2_	0.097	−0.084 to 0.298	12
Female progeny			
*r*_sp_	0.749	0.745 to 0.752	1,405,720
*r*_sm_	0.731	0.709 to 0.752	1,397,207
*r*_dm_	0.020	−0.022 to 0.067	3,566,966
*r*_dp_	0.243	0.238 to 0.246	20,545

*Note:* Relatedness between breeders of the same or different classes affects relatedness within and between families and subfamilies—for example, *r*_(Fis)_ of breeders affects *r*_sm_ (*r*_sp_) of progeny; *r*_q_ of queens affects *r*_dm_ of progeny; *r*_qm_ of breeders affects *r*_dm_ of progeny; *r*_m1_ of male breeders affects *r*_dm_ of progeny; and *r*_m2_ of male breeders affects *r*_dp_ of progeny (see Figure 1 and Table 1 for abbreviations).

^a^ Over dyads.

^b^ From 1000 or 5000 bootstrap replicates over dyads; for progeny estimates, from 5000 bootstraps over 10,000 randomly selected dyads.

#### 3.3.2 Relatedness of Nestmate Queens

Our newly produced genotypic data yielded relatedness estimates of unprecedented resolution. Although average pairwise relatedness between nestmate queens is zero (Figure 3b), nine individual nestmate queen dyads yielded unusually high relatedness values (Figure 3b). These stood out as significant outliers, with no analogous low outliers observed. Because limited marker panels are expected to produce large variance in individual pairwise estimates (Queller and Goodnight 1989; Städele and Vigilant 2016), these outliers may represent statistical noise. On the other hand, some or all may represent infrequent pairs of closely related nestmate queens. Previous results (Ross et al. 1996; Ross and Fletcher 1985a) are in agreement with our finding of negligible average nestmate queen relatedness.

#### 3.3.3 Relatedness for Other Classes of Nestmate Breeders

Although rarely estimated empirically (but see Azevedo‐Silva et al. 2020; De Pletincx and Aron 2022; Fournier et al. 2002; Hannonen et al. 2004; Thurin et al. 2011), the less familiar types of relatedness for other classes of nestmate breeders (*r*_qm_, *r*_m1_, *r*_m2_; Table 2) influence relatedness within and between families and subfamilies, thus affecting within‐colony genetic structure and overall progeny relatedness (*r*_p_). In common with the cases of mates and of nestmate queens (above), none of our estimates for these relatedness components in polygyne *S. invicta* are significantly greater than zero.

### 3.4 Progeny Relatedness

To estimate polygyne nestmate progeny relatedness in the wild, we applied the Huang et al. (2014) estimator to the most comprehensive dataset from a wild, invasive population suitable for estimating relatedness of worker (*r*_p(w)_) and gyne progeny (*r*_p(g)_) (Method S11; Ross 1993). Relatively low estimates (*r*_p(w)_ = 0.086 and *r*_p(g)_ = 0.103) were obtained, which reflect the moderate to high numbers of nestmate queens in that study (see Figure 4c) and the low relatedness within and between all classes of nestmate breeders (most recent estimates in Table 2).

We estimated average relatedness within (*r*_sm_, *r*_sp_) and between (*r*_dm_, *r*_dp_) matrilines and patrilines of female progeny in the *GA‐2018* and *GA‐2023* datasets based on the progeny assignment results (see Figure 3a). Values for *r*_sp_ and *r*_dp_ are close to the expected pedigree values (0.75 and 0.25, respectively), whereas *r*_dm_ is close to zero, as expected if most mother queens (and their mates) are unrelated (Table 2). The estimate for *r*_sm_ (0.73) is expected to be slightly below that for *r*_sp_ (0.75) because of a low effective frequency of polyandry.

A unique feature of the *GA‐2023* dataset is a temporal design that makes possible direct estimation of relatedness between pupae of each caste and a sample of the nurses that reared them through their larval development (*r*_p(gw)_, *r*_p(ww)_; Method S1). Our findings that *Q*_e_ is lower, and nestmate relatedness correspondingly higher, for gyne than worker pupae may suggest a scenario in which the sterile nurse workers in some way bias larval rearing so that gynes produced at a given point are more closely related to them than are worker daughters, thus providing them some extra measure of indirect fitness returns (Bargum and Sundström 2007). However, our estimates of the relatedness between potential nurses and the brood of each caste they reared are statistically indistinguishable (Figure S17), suggesting that no such bias occurs. Nonetheless, the average relatedness of gyne pupae to nurse workers (*r*_p(gw)_ = 0.262; 95% CI from 5000 bootstraps of single‐sample/colony estimates is 0.197–0.337) is substantially greater than both the relatedness of worker pupae to nurse workers (*r*_p(ww)_ = 0.209; 95% CI is 0.174–0.254) and the average worker relatedness (*r*_p(w)_ = 0.223; 95% CI is 0.192–0.258).

## 4 Discussion

### 4.1 Overview

The fire ant *Solenopsis invicta* is notable in that a fundamental breeding system component—the number of female breeders in a colony—is under strong genetic control (Kay et al. 2022; Keller and Ross 1998; Ross 1997; Ross and Keller 1998). One major conclusion of the present report, based on results from a new experiment complemented by reanalyzed data from earlier studies, is that many other breeding system components that differ between the social forms are influenced by the same genetic factor, the *Sb* supergene (see also Avril, Purcell, et al. 2019; Avril, Zahnd, et al. 2019; De Gasperin et al. 2024; Kay et al. 2022; Lawson et al. 2012). Monogyne colonies are simple families; that is, they are headed by a single queen mated to a single unrelated male, with nestmate daughters full sisters to one another, and they lack entirely the *Sb* element. The polygyne form has a far more complex breeding system, features of which are shaped by effects of the *Sb* supergene, so we focus our discussion on the breeding system of this form.

### 4.2 Number of Breeders

Polygyne colonies contain multiple, highly variable numbers of queens, according to field studies that directly counted them (Figure 4c) and a genetic survey that yielded indirect estimates (Ross 1993); regrettably, most of these field studies are limited by the fact that mated and unmated queens were not distinguished. Unmated wingless queens are relatively common in polygyne colonies in the invasive range (Tschinkel 2006), but because they produce no daughters and few, if any, sons (Vargo and Ross 1989), they are not “breeders” in a meaningful sense.

Numerous theories have been proposed to explain the evolution of polygyny and variation in queen number in invasive *S. invicta* (Fletcher 1983; Fletcher and Blum 1983; Ross et al. 1996; Ross and Fletcher 1985b) and other ants (Bargum et al. 2007; Dahan et al. 2021; Haney and Fewell 2018; Keller 1993; Kümmerli and Keller 2007a; Lecocq de Pletincx et al. 2019; Reiner Brodetzki et al. 2020; Ross and Keller 1995), with several sources of selection, such as nest site scarcity, limited queen longevity, and habitat specialization hypothesized to favor this type of social organization. Evolutionary analyses of polygyny in *S. invicta* are complicated by the presence of the selfish *Sb* supergene that controls colony queen number. In *Formica selysi* , another ant species with a supergene that regulates queen number and exhibits selfish behavior, a formal population genetic model showed that the conditions required for the evolutionary maintenance of both social forms are complex and restrictive with regard to the degree of assortative mating, fitness differences between supergene genotypes, and fertility differences between mating pairs (Tafreshi et al. 2022). Progress in resolving the evolutionary causes and routes to polygyny in *S. invicta* and other ant species with genetic regulation of social organization will benefit from similar comprehensive models as well as empirical studies of how genomic features such as supergenes or other distinct genetic modules yield variation in breeding system components (Kay et al. 2022; Lajmi et al. 2025). Such studies will link genetic and phenotypic variation with ecological features, uncover signs of selection on supergene or other relevant genomic sequences, and reconstruct pathways of social trait and genome evolution (Helleu et al. 2022).

The contrasting breeding systems in polygyne *S. invicta* populations from the native and invasive ranges add yet another dimension to the challenge of understanding the evolution of polygyny. In the native South American range, both relatedness between nestmate queens and relatedness between gynes and their nurses were reported to be substantially higher, and nestmate queen number lower, than in the U.S. (Ross et al. 1996), indicating an effect of recent colonization in shaping the breeding system (Eyer and Vargo 2021; Suarez and Goodisman 2021). The common occurrence of colonies with numerous unrelated queens may, in fact, be an artifact of recent invasion (within the past 100 years) of a novel environment. Social behaviors originally shaped by strong kin selection (under high nestmate relatedness) often persist inappropriately in invasive unicolonial ant populations, leading to the workers rearing unrelated brood and gaining no obvious fitness benefits (Helanterä et al. 2009). We note that, with moderate numbers of queens and significant maternity skew, low but significant relatedness between nurse workers and brood can be maintained and kin selection presumably will operate at some level.

The predominance of monandry in polygyne *S. invicta* queens coupled with strong paternity skew in the daughters of polyandrous queens corresponds to a maximum effective number of matings by queens of 1.10 estimated for any of the populations we examined (GA, FL, MS; Figure 5b). Thus, although variation in the number of male breeders and paternity skew can be important in modulating nestmate relatedness and genetic structure, it is inconsequential in these respects in *S. invicta* . On the other hand, the existence of even a low level of polyandry sheds light on some unorthodox effects of the social supergene on the breeding system. For example, we observed that multiply‐mated queens appear to develop into relatively heavy and fecund reproductives months or even years after their nuptial flights. These queens almost always mate first with a male with the *Sb* supergene haplotype—males that appear to be inferior to *SB* males in many reproduction‐related attributes (Table 3). We speculate that only queens that are especially robust in traits that allow them to stay aloft in mating swarms over an extended period are able to remate. In virtually all cases, queens remate with an *SB* male simply because of these males' numerical dominance in the swarms (most of them originate from monogyne colonies; e.g., Shoemaker and Ross 1996; Lawson et al. 2012). The robust traits that enable sustained flights, or associated traits, evidently also promote enhanced fertility later in life, although the physiological links between the two phenomena are not apparent. A finding consistent with this interpretation at the species level was reported in a comparative study of 16 *Cataglyphis* desert ant species; those with larger queens tend to have higher levels of polyandry (de Lecocq Pletincx et al. 2021), perhaps because larger queens have greater energy reserves to sustain lengthier mating flights. In *S. invicta* , it is unknown if the heavier, more fecund polyandrous queens are physically larger or simply experience greater ovarian development than monandrous queens.

**TABLE 3:** Comparison of reproduction‐related attributes of adult haploid males with each supergene haplotype in invasive polygyne *S. invicta* .

Supergene haplotype	Weight (mg)	Sperm count	Proportion of all males reared	Probability that mate (queen) remates	Share of worker offspring in doubly‐mated queens (range)	Share of gyne offspring in doubly‐mated queens (range)
*SB*	7.07	7.050E+06	16.7%–21.8%	< 1%	80.4% (66%–92%)	93.7% (83%–100%)
*Sb*	6.12	4.780E+06	78.2%–83.3%	40%–60%	19.6% (8%–34%)	6.3% (0%–17%)

*Note:* Entries for the proportion of all males reared range from values corrected for sample size per colony to uncorrected values. Entries in other columns are averages. Doubly‐mated queens include queens confirmed (*n* = 4) or presumed (*n* = 2) to have mated with both an *Sb* and an *SB* male. References: body size (Goodisman et al. 1999; Hettesheimer et al. 2024); sperm count (Lawson et al. 2012); proportion of adult males reared (Hettesheimer et al. 2024); all other values are from this report.

Polyandry generally is considered to be costly to females (Fromonteil et al. 2023), and several hypotheses have emerged to explain its evolution in social insects despite such costs (Baer 2016; Eyer et al. 2013; Kraus et al. 2005; Kronauer et al. 2011). In polygyne *S. invicta* , the occasional occurrence of remating can be explained in simple proximate terms; *Sb* males evidently are not competent to induce queen nonreceptivity and landing behavior following mating, perhaps due to insufficient sperm being transferred (see also Glancey and Lofgren 1985; Lawson et al. 2012; Tschinkel 1987) or to some other deficiency related to possession of the *Sb* supergene haplotype (Table 3). Remarkably, among the three polygyne populations we examined, the FL population is vast and contains few monogyne colonies as sources of *SB* males (Fritz et al. 2006; Lawson et al. 2012), with the expected result that a relatively high proportion of queens mate with an *Sb* male and then attempt to remate; the GA population has roughly equal frequencies of colonies of the two forms that are spatially intermixed (K.G. Ross, pers. obs.), while the MS population is small and isolated within a vast tract of monogyne, *SB* male‐producing, colonies (D. Shoemaker, pers. comm.). Consequently, frequencies of matings with *Sb* males and resultant levels of polyandry are predicted to fall in the order FL > GA > MS. This was observed (Figure 5d–f), clearly illustrating effects of the supergene on components of the breeding system (Avril, Purcell, et al. 2019).

### 4.3 Reproductive Skew

Analogous to decreasing breeder number, increasing the degree of reproductive skew among breeders raises average progeny nestmate relatedness (*r*_p_) by increasing the frequency of high‐value [within (sub)family] relative to lower‐value [between (sub)family] dyad relatedness values. This increase in nestmate relatedness (decrease in genetic diversity) due to increased skew is accompanied by a drop in effective breeder number.

Skew in both maternity and paternity is prominent in polygyne *S. invicta* (Figure 6a–c), as revealed by analyses of data from laboratory colonies comprising the two Georgia datasets. Moreover, this reproductive skew was more pronounced for gyne than for worker offspring. These results held through the duration of the *GA‐2023* experiment (7 months or more) and characterized each of the 10 study colonies. Gyne offspring provide direct fitness returns to breeders by becoming breeders themselves. In contrast, *S. invicta* workers, though required for colony function (and rearing gyne offspring), are sterile and provide no direct fitness benefits to their parents. Greater inequity in the maternity (Fournier et al. 2002; Kümmerli and Keller 2007a, 2007b) or paternity (Lattorff and Moritz 2016; Orr et al. 2024; Tilley and Oldroyd 1997; Withrow and Tarpy 2018) of reproductive than worker progeny has been reported in honeybees and several ant species, suggesting that reproductive competition between same‐sex breeders must be a pervasive selective force in social insect colonies with multiple breeders.

The prominence of such selection is illustrated by the persistent differential caste‐specific maternity skew observed in the *GA‐2023* colonies, especially the subset of five in which all of the queens lived through at least the four primary sampling periods. Gyne production is concentrated in the late spring in the polygyne form in northern Georgia (Vargo and Fletcher 1987). Our primary sampling periods spanned early spring through late fall, and the lifespan of polygyne queens is relatively short (1–3 years; Tschinkel 2006); thus, we captured a substantial fraction, if not the entirety, of each queen's lifetime reproductive success in these five experimental colonies (e.g., Figure 6e–g).

Fire ant workers are sterile, and nestmate queens generally are unrelated; hence, the pervasive caste‐specific maternity skew we found in this subset of colonies implies that certain queens are “winners” in terms of their lifetime fitness, while others are “losers.” One potential cause of such skew is segregating genetic variation for strong genetic influences (or persistent maternal effects) on caste determination (Anderson et al. 2008; Dyson et al. 2022; Schwander et al. 2010; Thompson and Chernyshova 2021; Weitekamp et al. 2017). However, the expected patterns of constant dominance in gyne production across samples, accompanied by correspondingly low production of worker daughters, were not observed. The *Sb* supergene‐induced loss of reproduction caused by the low or zero viability of *Sb*‐homozygous daughters demonstrably contributes to queens becoming losers if they mate only with *Sb* males. It is not clear in what developmental stage(s) this viability selection occurs, but our data suggest that, in gynes, it mainly occurs before the pupal stage. Selection against any remaining homozygotes continues through early adulthood, and they are completely absent among egg‐laying queens (DeHeer et al. 1999; Ross 1997). Matings giving rise to S*b/Sb* daughters are rare, however. Thus, the loss of offspring caused by this factor must be a minor contributor to the widespread maternity skew we document. A thorough investigation of the proximate factors associated with maternity skew in these colonies will be presented elsewhere.

The strong paternity skew we report almost always occurred in the context of variation at the supergene, with a queen initially mating with a male bearing the *Sb* haplotype subsequently attempting to remate (typically, with a *SB* male). Importantly, patrilines sired by the *Sb* mate were virtually always greatly underrepresented compared to the *SB*‐sired patrilines, a pattern evident in embryos as well as pupae (Figure 7a). This pattern of skew, with the *Sb* mate siring significantly fewer offspring than the *SB* mate, was temporally stable, with paternity shares comparatively constant across periods of up to 16 months. These observations imply that the sperm of a queen's two mates mix freely in her spermatheca and are accessed randomly for fertilization, as inferred for diverse social Hymenoptera (Eyer et al. 2013; Holman et al. 2011; Kronauer et al. 2006; Previtali et al. 2008; Stürup et al. 2014; but see Sundström and Boomsma 2000).

Intriguingly, values for the *M*‐index of paternity skew consistently were greater for gyne than worker offspring (Figure 6b). Moreover, the level of skew in favor of *SB* males was consistently greater than the average disparity in sperm production by males of the two haplotypes (Lawson et al. 2012), a pattern especially conspicuous for gyne daughters (Figure S15). This hints that *SB* males may have some added advantage in paternity over *Sb* males beyond their higher sperm counts, especially for gyne offspring.

This added advantage for *SB* males may stem from the diverse phenotypic effects of the *Sb* haplotype; specifically, *Sb* is a recessive deleterious variant in workers but a recessive lethal in the queen caste. Because of these caste‐specific viability effects of the supergene, the *Sb* mate of polyandrous queens should produce fewer viable daughter pupae than expected on the basis of sperm counts or genotype frequencies in embryos, and such patterns should be especially pronounced for gyne offspring, as we found. We note that only one queen in the *GA‐2023* dataset did not exhibit caste‐biased paternity skew that rose to statistical significance; queen P18‐01E was unique as well among polyandrous queens in having mated only with two *Sb* males. These results implicate genetic variation at the social supergene as a novel factor influencing caste‐biased paternity skew (see Table 3), differing from other proposed factors in social Hymenoptera, such as sperm competition in the spermatheca (Gençer and Kahya 2020), nepotism by nurse workers favoring their closest kin as new sexuals (Goodisman et al. 2007; Lattorff and Moritz 2016), or a predisposition of larvae of particular “royal” patrilines to develop as gynes rather than workers (Hughes and Boomsma 2008; Kümmerli and Keller 2007b; Moritz et al. 2005; Schwander et al. 2010; Tilley and Oldroyd 1997; Withrow and Tarpy 2018).

### 4.4 Breeder and Progeny Relatedness

Daughters in monogyne nests are full sisters related to one another by 0.75 (our best empirical estimate is *r*_p_ = 0.73), a colony genetic structure that provides little scope for genetic conflict among workers but substantial opportunity for natural selection to act at the level of the colony (Boomsma 2013; Wade 1985). That is, the genetic interests of colony members generally are closely aligned and in stark opposition to those of other colonies in the population.

Female progeny relatedness in polygyne *S. invicta* is low (≈zero) between and high (≥ 0.7) within matrilines. Nestmate breeders of all types are essentially unrelated to one another, so there are few, if any, high‐relatedness pedigree links between matrilines (*r*_q_, *r*_qm_, *r*_m1_ = 0). The mates of single polyandrous queens similarly are unrelated (*r*_m2_ = 0), so there is no elevation in the relatedness of half‐sisters (*r*_dp_) above the pedigree value of 0.25. Because inbreeding is absent (*r*_(Fis)_ = 0) and the paternity success of *Sb* males is low, overall female progeny relatedness in a polygyne nest (*r*_p_) is influenced almost completely by the number of mated reproductive queens and degree of maternity skew. Evidence we present points to skew being a major factor depressing effective colony queen numbers (*Q*_e_); this means that actual queen numbers must be sufficiently high to offset strong skew such that worker nestmate relatedness in invasive polygyne populations on different continents typically is quite low (maximum *r*_p_ = 0.20) (Henshaw et al. 2005; Kjeldgaard et al. 2022; Ross 1993; Ross and Fletcher 1985a; this report; Yang et al. 2008). Based on our analyses of queen numbers and skew from the *GA‐2023* dataset, this maximum *r*_p_ corresponds to a minimum *Q*_e_ of ≈3.75, which can be translated as actual colony queen numbers of no fewer than six (see Figure S20).

Because maternity skew is higher for gyne than for worker offspring, *r*_p(g)_ generally is expected to exceed *r*_p(w)_, as we document using the *GA‐2023* dataset (Figure S9). This suggests that unrelated nestmate queens compete for reproductive success, consistent with natural selection operating predominantly at the level of individual queens in invasive polygyne *S. invicta* . Viewed differently, low nestmate queen (and mate) relatedness, coupled with the rarity of polyandry and strong paternity skew, means that the disparity in relatedness of workers in the same vs. different matrilines is about as large as possible, reinforcing the notion of strong genetic conflict between cohabiting queens and between progeny workers of different matrilines. Moreover, low average *r*_p_ in polygyne colonies in the invasive range suggests that colonies or nests are not important units of selection (Helanterä et al. 2009). On the other hand, lower colony queen numbers and higher nestmate relatedness in polygyne colonies in the native range (Ross et al. 1997) suggest that colony‐level selection is important there.

We measured the relatedness of gyne and worker pupae to a set of adult workers representative of their nurses (*r*_p(gw)_ and *r*_p(ww)_, respectively) using the *GA‐2023* dataset. These relatedness values were found to be similar for the two offspring castes, suggesting that the sterile workers do not manipulate care of the brood in any way to enhance average relatedness between the sexual brood and them (Bargum and Sundström 2007) and that genetic factors or long‐term maternal effects do not influence caste determination in *S. invicta* . This result also is incompatible with the existence of royal matrilines, just as the consistent deficits in paternity by *Sb* males are incompatible with the existence of royal patrilines; either or both types of royal lineages have been implicated in some ants and in honey bees (Hughes and Boomsma 2008; Kümmerli and Keller 2007a, b; Moritz et al. 2005; Orr et al. 2024; Withrow and Tarpy 2018) (but see Wiernasz and Cole 2010). In the absence of any of these phenomena in polygyne *S. invicta* , the parameter of greatest interest is simply the average relatedness of gynes to their nurses, which provides a suitable measure of indirect fitness returns to the sterile workers (Kennedy et al. 2021). The value of *r*_p(gw)_, estimated at 0.26 in our *GA‐2023* dataset, suggests modest fitness returns to workers in laboratory colonies known to have quite low queen numbers and high maternity skew. An earlier estimate using adult workers (ages unknown) and young adult gynes in a wild population yielded *r*_p(gw)_ = 0.00 (95% CI −0.03 to 0.03) (Ross et al. 1996). In the absence of any higher‐level genetic structure among groups of nests (Goodisman and Ross 1998; Ross and Shoemaker 1997), the sterile workers in invasive polygyne *S. invicta* may receive quite low, but nonetheless biologically significant, indirect fitness benefits.

## 5 Conclusion

Our report provides a comprehensive analysis of the breeding systems in *Solenopsis invicta* , highlighting the pronounced influence of the *Sb* supergene on several components. Monogyne and polygyne colonies exhibit strikingly different kin structures, with monogyne colonies maintaining a single reproductive queen and high average nestmate relatedness, whereas polygyne colonies harbor multiple, largely unrelated queens, contributing to low average offspring relatedness and high potential for genetic conflict. The *Sb* haplotype disrupts mating dynamics, amplifies paternity skew, diminishes sperm production, and alters maternal contributions to the production of sexual daughters. The pronounced reproductive skew observed in polygyne colonies illustrates persistent reproductive competition that generates variation in fitness among nestmate queens and between the male mates of polyandrous queens. Future work will investigate the proximate determinants of reproductive success of polygyne queens and analyze breeding system components with respect to male offspring.

## Author Contributions

**Sierra Hale Walker:** investigation (equal). **Kip D. Lacy:** investigation (supporting), methodology (supporting), writing – review and editing (supporting). **Kenneth G. Ross:** conceptualization (equal), data curation (equal), formal analysis (equal), funding acquisition (equal), investigation (equal), methodology (equal), project administration (equal), writing – original draft (lead), writing – review and editing (equal). **Haolin Zeng:** data curation (equal), formal analysis (supporting), investigation (supporting), methodology (supporting), visualization (lead), writing – original draft (equal), writing – review and editing (equal).

## Conflicts of Interest

The authors declare no conflicts of interest.

## Supporting information


**Data S1:** ece371888‐sup‐0001‐supinfo.docx.

**Video S1:** ece371888‐sup‐0002‐VideoS1.mp4.

**Video S2:** ece371888‐sup‐0003‐VideoS2.mp4.

**Video S3:** ece371888‐sup‐0004‐VideoS3.mp4.

## Data Availability

All relevant original and aggregated data can be found at DRYAD (https://doi.org/10.5061/dryad.vhhmgqp5g).

## Endnotes

^1^
Based on numbers of published scientific papers that refer to nine genera of commonly studied eusocial insects in their titles, abstracts, or key terms for the period from 1972 (when *S. invicta* was formally described) through 2020 using the Web of Science Core Collection Indexes.

^2^
Note that our framework here does not explicitly consider male progeny, which will be discussed in future reports.
