# Supplementary material for: A Comprehensive Account of the Breeding Systems of the Fire Ant Solenopsis invicta
Source: Ecol Evol. 2025 Aug 25;15(8):e71888. doi: 10.1002/ece3.71888 (PMC12378010; doi:10.1002/ece3.71888)
Supplement: Supplementary file 1 — Data S1: ece371888‐sup‐0001‐supinfo.docx. [file ECE3-15-e71888-s003.docx]

**Supplemental Information for:**

**A Comprehensive Account of the Breeding Systems of the Fire Ant *Solenopsis invicta***

Sierra Hale Walker, Kip D. Lacy, Kenneth G. Ross, Haolin Zeng

# Supplemental Information – Methods

Method S1: *GA-2023* dataset

### Ant Collection and Rearing.

Ten polygyne *S. invicta* colonies were collected in their soil nests in 5-gallon talc-dusted buckets in the field in Clarke, Oconee, and Oglethorpe Counties, Georgia, in February of 2018. Each nest was spaced at least 20m from other collected nests. Upon return to the laboratory, the ants were separated from the soil by slowly dripping water into each bucket to induce them to float on the water’s surface, enabling transfer of the relatively intact colonies into plastic enclosures (26×40×8 cm trays) containing 14-cm-diameter round plastic nests with dark covers and moistened plaster bottoms (Jouvenaz et al., 1977; Ross, 1988). The enclosures with their live ants were maintained in a controlled-environment insectary in which the photoperiod (14hrs light:10hrs dark), temperature (32°C), and humidity (25% RH) were held constant. Colonies were provisioned with water and food (a high-protein diet [tuna/dog food/peanut butter mix], a high-carbohydrate diet [assorted vegetables/granulated sugar mix], and frozen crickets and mealworms) (Ross, 1988; Trible and Ross, 2016). Each experimental colony was confirmed to be of the polygyne social form by finding multiple wingless (reproductive) queens and by determining the presence in hundreds of workers per colony of the *C294****^92^*** microsatellite allele found only in the polygyne-specific *Sb* supergene haplotype (Ross and Shoemaker, 2018).

Because wild colonies were used as the source of the experimental colonies, ages of the focal queens were unknown; however, the colonies were collected in late February and queen recruitment apparently occurs primarily in the late spring (Glancey and Lofgren, 1988; Goodisman and Ross, 1999), so all these queens are presumed to have been at least nine months old at the onset of the experiment.

### Experimental Colony Set-up.

Once the colonies were habituated to the insectary (2-3 days), all sexual forms (males and gynes^[[1]](#footnote-2)^ in the 4^th^ instar larval, pupal, or adult stages) were separated and removed from each colony by sieving the ants with a #14 grade geological sieve (1.4mm apertures). To further prepare the ten colonies for use in the experiment, the number of reproductive queens was then reduced to five in half of the colonies and to eight in the remaining half. The 65 reproductive queens retained were uniquely marked using a single or two spots of Markal® Ball Paint (Wojcik et al., 2000) on the dorsum of the alitrunk (thorax) to serve as a unique identifier by which each could be distinguished through the course of the experiment (see also Fig. 2; Movie S1).

Four primary sampling periods spanned much of the study, with each period culminating in a sampling point at which worker and gyne pupae were collected for genotyping. At the start of the first sample period in April 2018, all ten colonies were sieved a second time to remove all sexual adults and brood that had appeared since the initial sieving. This was done to ensure that all of the subsequently appearing gyne pupae and young adults were daughters of the paint-marked queens rather than offspring carried over from previous resident queens. The time of this second sieving is considered Day 1 of the experiment. The period between each of the first four (primary) pupal sampling points was approximately 45 days. This interval was chosen to balance the requirement for relatively even sample coverage over the expected 6-9 month study with a need for sufficient temporal resolution to detect crucial short-term changes in queen fecundity or sperm usage over their relatively brief life spans (polygyne *S. invicta* queens are thought to live 1-3 years [Tschinkel, 2006]). Also, by virtue of this timing of sampling and known developmental periods of the different castes’ brood stages (O’Neal & Markin, 1975; Porter, 1988) worker pupae at one sampling point were expected to be 1-3 week old adults during the early larval stages of the individuals sampled at the next sampling point. These young adults largely comprise the pool of nurses that feed and otherwise tend larvae (Cassill & Tschinkel, 1999; Porter & Tschinkel, 1985; Tschinkel, 2006) and play a role in determining the caste fate of larval daughters via the options of differential feeding and/or destruction. Thus, worker pupae sampled at one point were likely to have acted as brood nurses for pupae sampled at the following point; the relatedness between worker pupae in one sample and pupae of each caste in the subsequent sample therefore was estimated to approximate nurse-larva relatedness.

Six of the ten colonies were sampled at one to three additional sampling points beyond the primary four, with the final sample collected 69 weeks after the start of the experiment.

### Sample and Data Collection.

At each sampling point marking the end of a sample period, young pupae of both workers and gynes were collected. We weighed all marked queens, measured their oviposition rates, and took samples of their eggs 20 and 27 days prior to each collection of worker and queen pupal samples, respectively. Oviposition rates were measured by isolating queens with 2-3 adult workers from their colony in 6 mL plastic specimen cups with moistened plaster bottoms for 4 h (Fletcher et al., 1980). Queens were then returned to their nests and the numbers of eggs laid were counted (e.g., Ross and Shoemaker, 2018).

For each of sample periods 2-4, we instituted an additional weigh-in of the queens at a point about halfway between the previous pupal sampling point and the next weigh-in/oviposition test linked to the subsequent pupal sample (Fig. 2; Movie S1). The additional weigh-in was done to improve temporal resolution of the dynamics of queen oviposition rates without stressing queens unduly by subjecting them to extra oviposition tests (weight and fecundity are highly correlated in *S. invicta* reproductive queens―(K. G. Ross, 1988) (Fig. S4). Following Sample 4, all colonies reduced their growth as winter approached; but the six colonies that resumed growth near the end of winter were sampled 1-3 additional times at varying intervals (Sampling Periods 5-7). Queen weigh-ins and oviposition tests were conducted opportunistically at least once between the time each of these supplemental samples was taken.

Either 95 (5-queen colonies) or 190 (8-queen colonies) young (white, pink-eyed) pupae of each caste were collected at each of the four principal sampling points; in a few instances, sufficient numbers of gyne pupae were not available, so older, darker pupae or newly emerged (teneral) alates were collected opportunistically. The resulting range of age differences of up to about 10 days is unimportant for most analyses; other analyses were done with these older individuals excluded. Upon completion of offspring sampling, all remaining sexuals of all stages were removed from colonies to prevent carry-over to the next sampling point.

Following conclusion of the fourth sampling period in October 2018, additional samples were collected at varying intervals from six colonies on an opportunistic basis. Up to three of these supplemental samples were collected (in two of the colonies) before most focal queens in a colony had died and the colony was significantly weakened. The two longest-surveyed colonies were sampled seven times each over a period of 15 months. We focus on the first four (primary) samples for most analyses.

Colonies were inspected daily to discern their general health based on the amount and type of brood, as well as to locate every queen to ascertain her well-being. Queens that died between observations or that clearly were being attacked by workers (which inevitably leads to their death; Keller and Ross, 1998) were immediately collected and frozen at -80^○^C pending subsequent genotyping. Seven queens (10.8% of total) died without their bodies being recovered, so genotypic data were not directly available for them or the sire(s) of their female offspring. Nonetheless, the parental genotypes at most loci could be reconstructed from the daughter genotypes in the process of parentage assignment (Method S3).

Sampled female progeny, as well as almost all reproductive queens and their mates were genotyped at 13 microsatellite marker loci (Method S3; Ross & Shoemaker, 2018).

Method S2: *GA-2018* dataset

The principal data set was supplemented by a second set derived from an earlier study of transmission ratio distortion in polygyne *S. invicta* queens from twelve colonies obtained from the same area of northern Georgia as the colonies collected for the *GA-2023* dataset (K. G. Ross & Shoemaker, 2018). These colonies were established in our insectary, and mated reproductive queens from each were removed sequentially to be isolated for 12 h with a few adult workers in small specimen cups. The queens were then frozen and the eggs they laid (actually, embryos in the egg coat by this point) were maintained with the workers for another 48h, at which point they were frozen. Genomic DNA was extracted from the mother queens and individual embryos (K. G. Ross & Shoemaker, 2018), and genotypes at 13 microsatellite loci were determined for 3346 diploid embryo offspring of 101 queens and 105 of their mates. These genotypic data comprise the *GA-2018* dataset (see also Movie S2).

Method S3: Microsatellite data quality control and matriline/patriline assignment

Individuals sampled for the *GA-2018* and *GA-2023* datasets were genotyped at 13 microsatellite marker loci (Ross & Shoemaker, 2018) (Table S1). Details of the DNA extraction procedures, PCR protocols, sequencing of amplicons, and scoring of sequence chromatograms common to both studies are detailed elsewhere (Ascunce et al., 2009; Ross & Shoemaker, 2018). Briefly, appropriately diluted genomic DNA from whole embryos/pupae, queen heads, or queen spermathecal contents was used as template in multiplex PCR reactions; DNA from the latter two sources was used to directly determine the genotypes of queens and haplotypes of their mate(s). The PCRs based on the spermathecal contents failed in ca. 15% of cases, but each fathers’ multilocus haplotypes were readily inferred from a combination of the mother’s and presumed daughters genotypes by a process of reciprocal illumination. One primer of each of the 13 primer-pairs was labeled at the 5' end with one of four fluorescent dyes, and primer pairs were combined in three separate multiplex reactions. The resulting PCR amplicons were diluted and pooled into a single plate for injection into an ABI-3730XL 96-capillary sequencer (Applied Biosystems). All microsatellite genotypes were scored initially from sequence chromatograms using the software GENEMARKER (SoftGenetics). All chromatograms were inspected to confirm the accuracy of the software genotype calling, with special attention paid to suspect calls (e.g., daughter genotype calls not matching any other nestmate genotype). We note that errors in genotype scoring caused by allelic dropouts, stutter bands (PCR products that result from slippage during DNA replication), DNA contamination of samples, or other causes must be rare in both datasets. Because queens of *S. invicta* generally mate with a single haploid male, it is usually straightforward to predict daughters’ multilocus genotypes from parental genotypes. Scoring errors lead to inconsistencies in the parent/offspring genotypes and difficulty assigning daughter pupae to matrilines, deviations from Mendelian genotype distributions above the level due to sampling error, and deviations from Hardy-Weinberg equilibrium (HWE), none of which was prevalent (below).

Similar comments apply to our ability to confidently assign female progeny to matrilines and patrilines. Such assignments were simplified because the multilocus mothers’ genotypes and fathers’ haplotypes generally were known (fertile males are haploid in *S. invicta* as in other Hymenoptera). Along with parental genotypic data, information on dates of queen death or loss was helpful in excluding specific queens (and males) as parents when queens were no longer alive during a relevant oviposition window (*GA-2023* dataset). General conformation of the observed genotype frequencies to HWE at all loci except the two on Chr16 linked to the *Sb* supergene (below), as documented in Table S1, supports the robustness of our genotype scoring and family assignments, and further suggests that null alleles are not prevalent in our marker set and study populations; this latter conclusion is supported also by specific tests for null alleles and PCR failures (Table S1). Finally, for the *GA-2023* dataset, inspection of the ratios of genotypes at segregating loci in progenies comprising ≥20 individuals gave us further confidence in our ability to recognize and effectively assign almost all genotyped offspring to their correct family/subfamily. In only 27 of the 402 locus/progeny combinations segregating two genotypes (6.7%) did these genotypes fail to occur in Mendelian (1:1) proportions according to binomial tests (α=0.05); this proportion does not differ significantly from the expected proportion of chance failures due to sampling error with our sample sizes when no deviations from Mendelian proportions exist (randomization test; 5000 replicates; *P*=0.156). These analyses and results, interpreted holistically through a process of reciprocal illumination, give us confidence in our parentage assignments in the *GA-2023* dataset.

Method S4: Sources of additional genetic data

Genetic data from several earlier studies were extracted and compiled for specific analyses. Extensive allozyme genotypic data (ten loci) for a large polygyne population in northern Georgia (Walton County) were used to estimate nestmate relatedness for workers (several life stages combined) and the inbreeding coefficient (*F*_IS_) for adult reproductive queens (data from Ross, 1993). Similar genotypic data were extracted for a sample of polygyne nestmate reproductive queens from central Texas (Travis County; six loci), and a sample of monogyne nestmate non-reproductive adult gynes from northern Georgia (Morgan, Oconee, and Walton Counties; seven loci) (data from Ross et al., 1996; Ross and Shoemaker, 1997) to estimate nestmate relatedness. Allozyme (nine loci) and microsatellite (seven loci) data from six monogyne populations located throughout the southern U.S. (one gyne from each nest) were aggregated to estimate *F*_IS_ (Shoemaker et al., 2006). Two additional microsatellite data sets were compiled as well to estimate this statistic in the monogyne form. The first set comprised genotypic data from Ascunce et al. (2011) for 12 microsatellite loci scored in samples of monogyne gynes (one per nest) in northern Georgia (several unspecified counties); the second comprised allele frequency data from 105 marker loci (99 microsatellites, six allozymes) scored from newly mated monogyne queens recovered from a single nuptial flight in northern Georgia (Oglethorpe County) (data retrieved from Ph.D. dissertation of M. A. Fisher [Univ. of Georgia; 2013]). Finally, for comparison of queen mating patterns among geographic regions in the southern U.S., supergene genotype or haplotype data for 84 (Florida polygyne), 92 (Florida monogyne), and 88 (Mississippi polygyne) reproductive queens and their mates reported in Lawson et al. (2012) were compiled for analysis.

Method S5: Choice of relatedness estimator

We first evaluated the performance of each of 19 estimators using simulations based on our genotype data (from the *GA-2018* and *GA-2023* data sets [microsatellites] and from Ross (1993) [allozymes]), conducted with the software package PolyRelatedness 1.11b (Huang et al., 2014). The simulations estimated the bias and variance for values generated by each estimator relative to pedigree *r* values for six specific simulated relationships as well as simulated pairs (dyads) of unrelated individuals. The performance of the estimators varied considerably in these simulations (Fig. S1). The ML (maximum likelihood) estimators often returned upwardly biased estimates across relationships for both the allozymes and microsatellites, a common issue with this class of estimators when used with modest-size marker sets (Milligan, 2003; Huang et al., 2015). Among the overall best performers for both types of markers was the MOM (method of moments) estimator of Huang et al. (2014).

To further test the performance of the Huang et al. (2014) MOM estimator, we compared it with the Huang et al. (2015) ML estimator, or with pedigree *r* values, using real-life data. The Huang et al. ML estimator was selected because it performed similarly on the two types of data (allozymes and microsatellites) in the simulations, it was representative of ML estimators in the simulation results, and it was developed by the same research group as the MOM estimator. A second ML estimator not represented in the simulations (Kalinowski et al., 2006) was included along with the Huang et al. ML estimator in the first of four evaluations.

For all the Georgia microsatellite-based *r* estimates in this report, a specified reference (base) population consisted of 172 queens and 166 of their haploid mates from the 22 colonies in the *GA-2018* and *GA-2023* datasets. These individuals provide an unbiased estimate of the allele frequencies for polygyne *S. invicta* in northern Georgia and constitute an ideal reference set for microsatellite-based relatedness estimation—the reproductive individuals comprising it are genetically independent (non-inbred and unrelated), they presumably represent the founding population in northern Georgia, and, in most cases, the focal individuals for which *r* was estimated are not included in the reference set (see Weir et al.,, 2006; Kümmerli and Keller, 2007; Powell et al., 2010; Wang, 2014; Weir and Goudet, 2017; Waples et al., 2019 for importance of reference set). All allozyme marker-based estimates used the entire collection of samples in the analysis as the reference population.

The first evaluation comparing the MOM and ML estimators involved microsatellite (msat) genotypes of polygyne reproductive nestmate queens from northern Georgia (Fig. S2a). Their average relatedness (*r*_q_) estimated using the MOM estimator was statistically indistinguishable from zero, as reported earlier using allozyme markers and estimators different from those tested here (Ross and Fletcher, 1985; Ross et al., 1996). The two tested ML estimators returned estimates approaching 0.1, whether nests or individuals were weighted equally in taking the averages. This result is consistent with the reported upward bias of ML estimators with modest-size marker panels (Csilléry et al., 2006; Huang et al., 2014; Wang, 2014; Huang et al., 2015) and with our simulation results showing such bias for several ML estimators, including that of Huang et al. (2015).

This upward bias is probably due to a characteristic feature of ML estimators that does not pertain to most MOM estimators, which is to preclude in the estimation procedures “biologically implausible” values outside the range [0-1] (Huang et al., 2014, 2015; Milligan, 2003; Wang, 2014). Thus, our next evaluation compared (i) the MOM estimator, (ii) this estimator with each set of pairwise estimates truncated to fall between 0 and 1, and (iii) the Huang et al. (2015) ML estimator, using nestmate queens from various localities genotyped with different marker types (Fig. S2b). Again, the ML estimates appear inflated, comparable to the truncated MOM estimates.

Next, we compared the performance of the MOM and ML estimators on structured and unstructured data sets; both consisted of polygyne nestmate queen msat genotypes from the *GA-2018* and *GA-2023* data sets, but the structured version contained information on each individual’s nest of origin while the unstructured version did not. Because the reference population was the same as the test population, and dyad *r*_q_ values for the entire data set are averaged when information on nest membership is not provided, an unbiased estimator is expected to return a value of zero for the unstructured data set (Goudet et al., 2018; McGuire et al., 2022). While the two MOM estimates were statistically indistinguishable from zero, both ML estimates were inflated (Fig. S2c). This effect was demonstrated also with a very different data set containing allozyme genotypes for monogyne nestmate gynes (Fig. S2c inset). While, in this case, both estimators returned *r*_p_ values close to the expected 0.75 for the structured data, for the unstructured data the MOM estimate was zero, as expected, while the ML estimate again was inflated, to > 0.2.

Finally, we compared the estimates from the MOM estimator with pedigree *r* values for individuals of suspected or known relationship (as determined by maternity/paternity assignment in this report). In all four instances, the estimates were very close to the expected (polygyne nestmate queens) or pedigree values (Fig. S2d).

In view of these results, all estimates of *r* in the present study were obtained using the Huang et al. (2014) MOM estimator.

Method S6: Numbers of breeders

**Queen numbers in polygyne nests in the wild**

We aggregated and summarized data from 14 studies in which polygyne nests at various locales in the southern U.S. were thoroughly excavated and attempts were made to count all wingless, presumably reproductive queens. Typically, in the invasive range, a substantial proportion of such queens is unmated and cannot produce daughters (Ross et al., 1996). Many reports did not distinguish between mated and unmated queens, which requires dissection of the spermatheca or isolation of queens to determine if they produce daughters.

**Metrics for breeder numbers**

Three different metrics for numbers of breeders in a social group, or their derivatives, are commonly used in studies of breeding systems (Crozier & Pamilo, 1996; Hannonen et al., 2004; Kronauer et al., 2006; Kümmerli & Keller, 2007b; Loope et al., 2014; Queller, 1993; K. G. Ross, 2001). The first two are means and thus are summary measures of breeder number over multiple colonies or queens. The third metric (effective number) can be calculated for single nests or single queens but also is commonly used as a summary statistic.

Arithmetic mean number (*Q*_x̅_; *M*_x̅_): The arithmetic mean (average) shows the central tendencies in direct counts of breeders, families, or subfamilies per social group.

Harmonic mean number (*Q*_h_; *M*_h_): The harmonic mean, the reciprocal of the average of the reciprocals of the data points, is a summary measure that gives more weight to smaller values in a series, such as numbers of queens per nest in a population. The harmonic mean scales more closely to the changes in *r*_p_ caused by changes in breeder number than does the arithmetic mean, especially when low numbers of breeders occur commonly (see Movie S3). It accounts for variation in colony queen number or queen mate number and can be conceptualized as the number of breeders per colony that would give rise to the observed level of genetic variation observed in the collective offspring (*r*_p_ or *r*_sm_) were there no variation in the numbers of such breeders. With such variation, the harmonic mean number of breeders will be lower than the arithmetic mean (Movie S3). Wade (1985) first pointed out that the harmonic mean number of breeders is a more useful summary statistic than the arithmetic mean for characterizing the potential for social evolution because of its closer relationship to *r*_p_ (or *r*_sm_).

Genetically effective (mean) number (*Q*_e_; *M*_e_): The effective numbers of queens (*Q*_e_) or their male mates (*M*_e_) account for both variation in colony queen number or queen mate number and variation in maternity shares in polygyne colonies or paternity shares in polyandrous queens. They can be conceptualized as the number of breeders that would give rise to the observed level of genetic variation observed in the offspring (*r*_p_ or *r*_sm_) were there no variation in breeder numbers and no reproductive skew. The effective number of breeders should be lower than both the arithmetic and harmonic mean numbers when skew is present (Trontti et al., 2005; Orr et al., 2024) (see Movie S3 for explanation). Note that the effective number of breeders can be calculated using two different approaches—directly by assigning progeny to matrilines/patrilines and calculating the variance in parentage shares, or indirectly, by estimating average pairwise genetic relatedness in colonies or (sub)families.

The first, direct, approach uses the known variance in parentage derived from empirical assignments of offspring individuals to families or subfamilies to calculate effective breeder number by means of one of several different estimators (below). The second, indirect, approach makes use of empirically derived estimates of the average pairwise genetic relatedness within colonies/families/subfamilies to calculate effective breeder numbers expected to produce such relatedness values, using appropriate equations (e.g., Equations (1) and (4) in Ross [2001]).

We compared the two approaches by estimating effective numbers of queens per nest (*Q*_e_) using genotypic data from 11 microsatellite loci for 1347 worker pupae from ten colonies sampled at one time point in the *GA-2023* experiment. Estimates from the two approaches agreed well with one another (Fig. S3).

Several different estimators of effective breeder numbers are available for the direct approach, including Equations (4.15) and (4.20) in Crozier and Pamilo (1996), Equations (2) and (6) in Ross (2001), and Equation (16) in Nielsen et al. (2003). We compared the performance of the three estimators of the effective numbers of male mates (*M*_e_) of each of 14 polyandrous queens, based on the *GA-2018* and *GA-2023* datasets. The different estimators returned similar values of *M*_e_ (Fig. S18). Unless otherwise indicated, the equations of Ross (2001) are used in the direct approach to estimate effective breeder numbers for data from the *GA-2018* and *GA-2023* experiments.

Method S7: Reproductive skew

### Maternity skew

In a first analysis to test how widespread and prominent maternity skew was in the *GA-2023* data set, values of the *M*-index (Ross et al., 2020) were calculated separately for each caste and colony at each of the four primary sampling points. *M*-index values of zero mean that the apportionment of maternity is distributed as expected under a random multinomial model with equal apportionment, *M*>0 means that skew occurs, and *M*<0 means that maternity is shared more equally than expected under the model. From each of our eight sets of ten values were taken 1000 bootstrap samples that were then used to compute the mean and 95% CIs for each set. Each of the eight sets of values was judged to be significantly greater than zero based on the 95% CIs, indicating significant and widespread skew in the maternity of offspring of each caste (minimum for workers: *M* = 0.618 [95% CI, 0.366-0.911]; minimum for gynes: *M* = 1.329 [0.963-1.737]). Almost all values for gyne pupae were substantially greater than 1.0, while in all cases for workers, 0 ≤ *M* ≤ 1.0.

A follow-up simulation analysis of up to seven sampling points with three or more matrilines per colony tested which, if any, of the 75 observed maternity apportionment patterns could be explained solely by sampling error. This was achieved by simulating a population of 10,000 daughter pupae to which each of *N* mother queens contributed 1/*N* ● 10,000 of the pupae. A subset of 500 of these daughters was randomly sampled, then a second subset of *n* of the 500 daughters was again randomly sampled, where *N* equals the number of matrilines and *n* the number of offspring collected for the focal empirical colony sample. Both the *S*_3_ skew statistic (Pamilo & Crozier, 1996) and the *M*-index of skew were calculated for 200 simulation iterations for each of the 75 empirical samples. The rank of the empirically estimated values in the list of 200 randomly generated ones was taken to be the probability that as large or larger skew values could arise due solely to sampling effects. In only three of the 75 cases were the empirical values above the 95^th^ percentile of the simulated ones using either estimator of skew; all involved worker progeny (colony P18-01 Sample 2; colony P18-10 Samples 1 and 2).

The apparent difference between the castes evident in the analyses above prompted more formal tests in the form of a resampling bootstrap difference routine to determine if values of the *M*-index are significantly greater for gyne than worker offspring after accounting for repeated sampling. For a randomly selected primary sampling point (1 – 4), the value of the *M*-index for worker pupae was subtracted from the value for gyne pupae for each colony (with replacement), yielding ten values; the bootstrapped mean of these ten values was recorded and the process was repeated for a total of 1000 iterations. The probability of a result as or more extreme than that observed was taken as the proportion of the 1000 bootstrap difference values that were less than zero (the null hypothesis is that the differences average zero). Only eight of the 1000 iterations yielded values ≤0 for the pseudo-distributions of *M*-index_gyne_ – *M*-index_worker_, suggesting *P*=0.008 for the null hypothesis that maternity skew is equal for the two offspring castes.

### Paternity skew

Overall, the distribution of paternity *M*-index estimates (including all samples regardless of colony of origin, sampling point, or caste of pupae) is similar to that of maternity *M*-index estimates for nestmate queens (Fig. S11). Essentially identical analyses were conducted for paternity skew as for maternity skew.

To learn if differences in viability of *Sb/Sb* supergene homozygotes between the castes may be responsible for observed caste-specific differences in paternity skew, we first summarized supergene genotype proportions in diploid embryos and female pupae of each caste produced by queens in colonies from the *GA-2018* and *GA-2023* datasets, respectively. The point estimates (and 95% CIs for genotype *Sb/Sb*) were derived using a resampling approach in which a single genotype per patriline/matriline was drawn randomly (with replacement) 1000 times then finding the mean and 2.5^th^–97.5^th^ percentiles for the resulting distributions. The probability that the proportions of *Sb/Sb* individuals in two groups did not differ was taken as the proportion of iterations at which this genotype was less frequent in the focal group of pupae than in embryos or in pupae of the other caste.

Method S8: Analyses of remating probabilities

### Part 1: Base model

Based on our understanding of fire ant mating habits (Tschinkel, 2006; see also video at <https://youtu.be/0Q4iqLyqA34>) and on data concerning the effect of the *Sb* supergene on the mating system (Lawson et al., 2012; this report), we developed a model to assess the probabilities that polygyne queens will remate after first mating to a male of each supergene haplotype (*SB*, *Sb*). In this model, we first make the following assumptions:

1. In mating swarms, individual *SB* and *Sb* males have an equal probability of successfully mating with a queen; that is, males of each haplotype are equally capable of finding, grasping, and successfully inseminating a flying queen. (This assumption is possibly false. Because *SB* males are on average larger and heavier than *Sb* males [Hettesheimer et al., 2024], *SB* males may also be physically stronger and thus have a higher probability of successful copulation than *Sb* males. However, such a difference does not affect model results; see Part 2 below.)
2. The male composition of the mating swarm is equal to the proportional representation of sperm of each haplotype collectively in the spermathecae of reproductive queens in the population (M*_SB_* and M*_Sb_*, respectively); in other words, the mates of reproductive queens in polygyne nests are assumed to be representative of the male haplotype makeup of the mating swarm. (This assumption is also possibly false; see Part 2.)
3. Males do not mate with more than a single polygyne queen. This assumption is supported by the fact that the number of sperm in the seminal vesicles of mature adult males (mean of 7.05 and 4.78 million, respectively, for *SB* and *Sb* males; [Lawson et al., 2012]) is not sufficient to fully inseminate a second queen (males do not produce sperm after eclosing as adults). Newly mated queens hold an average of 7.3 million sperm in their spermatheca (Tschinkel, 2006), closely matching the sperm counts for *SB* males.
4. A queen exerts no female choice in the mating swarm; that is, mating is random with respect to a male’s supergene haplotype, with a queen and male pairing as they randomly encounter one another in the swarm. Thus, the probability of a queen mating with a male of each haplotype is directly proportional to the relative abundance of such males in the mating swarm.
5. After the first mating, the probability that a queen will mate with a second male depends on the first male’s supergene haplotype. These remating probabilities are denoted by P_(re-_*_SB_*_)_ and P_(re-_*_Sb_*_)_ for *SB* and *Sb* males, respectively. The second mating is similar to the first in that the queen does not choose a mate based on his supergene haplotype, making the probability of mating with a particular haplotype, as before, equal to its proportional representation among males in the mating swarm.
6. Diploid males are not important. This is based on the finding that fewer than 0.1% of mated polygyne queens produce offspring sired by diploid males (this report), presumably because the vast majority of such males are sterile (Krieger et al., 1999).

Based on these assumptions, we can calculate the hypothetical number of queens of each mating type (nQ_x_), where x is the male mate haplotype(s), with the following equations:

nQ*_SB_* = nQ_tot_ * M*_SB_* * [1-P_(re-_*_SB_*_)_] ………………………………………...……...….. equation 1

nQ*_Sb_* = nQ_tot_ * M*_Sb_* * [1-P_(re-_*_Sb_*_)_] ……………………………………….…….……… equation 2

nQ*_SB+SB_* = nQ_tot_ * M*_SB_* * P_(re-_*_SB_*_)_ * M*_SB_* ………………………………………..….… equation 3

nQ*_Sb+Sb_* = nQ_tot_ * M*_Sb_* * P_(re-_*_Sb_*_)_ * M*_Sb_* …………………….....….………………….. equation 4

nQ*_SB+Sb_* = nQ_tot_ * M*_SB_* * P_(re-_*_SB_*_)_ * M*_Sb_* + nQ_tot_ * M*_Sb_* * P_(re-_*_Sb_*_)_ * M*_SB_* …………........ equation 5.

We then compute for each nQ the error between the observed number of queens and hypothetical number estimated by the model, for instance:

Error i = [nQ*x* – (observed Q*x*)], so Error 1 = nQ*_SB_* – (observed number of queens that mated once with an *SB* male).

The total error is the sum of the five types of error corresponding to the five predicted nQ*_X_*:

Error_total_ = Error 1 + Error 2 + Error 3 + Error 4+ Error 5

The combination of P_(re-_*_SB_*_)_ and P_(re-_*_Sb_*_)_ that yields the lowest Error_total_ value represents the best estimate of these two central parameters.

Taking the Georgia population as an example, with the *GA-2018* and *GA-2023* data, we first obtain the proportion of *SB* and *Sb* males (M*_SB_* and M*_Sb_*) in the mating swarm: M*_SB_* = 0.853 M*_Sb_* = 0.147.

For simplicity, we consider nQ*_Sb+Sb+SB_* as nQ*_SB+Sb_* in the error calculation, as queens rarely mate three times and, when this occurs, the third male sires very few offspring (this study).

Using a grid search algorithm to locate the minimal total error (Error_total_), the estimated number of queens of each mating type was compared to the observed number, with the results represented by a "heatmap" of possible P(re-*Sb*) and P(re-*SB*) values on the grid (Fig. 5h). The best match between the model-estimated and empirically observed values for number of queens of each mating type gives:

Error_total_ = 5.2895; P_(re-_*_Sb_*_)_ = 0.6012; P_(re-_*_SB_*_)_ < 0.001.

In other words, this model estimates that queens mating with an *Sb* male have a 60% probability of remating, whereas those mating with an *SB* male have less than a 1% chance of remating.

Taking the same approach, for the Florida population, the best estimate gives: minimal error = 2.3108, P_(re-_*_Sb_*_)_ = 0.5731, P_(re-_*_SB_*_)_ = 0.0400 (Fig. 5i). For both populations combined, the best estimate gives: minimal error = 3.0183, P_(re-_*_Sb_*_)_ = 0.5551, P_(re-_*_SB_*_)_ = 0.0140.

### Part 2: Adjusted model

If assumption #1 was false, our estimation of the number of queens in each mating category is unaffected because it does not require knowledge of the differences in mating success between male genotypes. A queen mates with an *SB* or *Sb* male with a certain probability, which depends on both the availability of males and the effectiveness of mating. This “true mating probability” for each male genotype can be interpreted as the “effective proportion” of male haplotypes in the mating swarm, denoted as E***_SB_*** and E***_Sb_***.

If assumption #2 was false, we would need to find the “effective proportion” of each male haplotype in a mating swarm. These effective proportions inherently account for phenotypic differences between the two male types and are influenced only by the remating probabilities. (From another perspective, the remating probabilities also indirectly reflect the fitness differences between the male genotypes.)

If remating does not occur, for example, the proportions of *SB* and *Sb* males calculated from queen mating types will represent the “effective” proportions (E*_SB_* and E*_Sb_*), as a queen will either mate with an *SB* or *Sb* male. But if remating does occur, we can apply the probabilities of remating on effective proportions to weight the contributions of each male type, leading to the “observed” male genotype proportions O*_SB_* and O*_Sb_* (calculated by genotype counts from reproductive queen spermatheca and offspring, equivalent to M*_SB_* and M*_Sb_* in part 1):

O***_SB_*** = E*_SB_* * (1 - P_(re__*_SB_*_)_) + E*_SB_* * P_(re__*_SB_*_)_ * E*_SB_* * 2+ E*_Sb_* * P_(re__*_Sb_*_)_ * E*_SB_*

O***_Sb_*** = E*_Sb_* * (1 - P_(re__*_Sb_*_)_) + E*_Sb_* * P_(re__*_Sb_*_)_ * E*_Sb_* * 2 + E*_SB_* * P_(re__*_SB_*_)_ * E*_Sb_*

We can then express E*_SB_* and E*_Sb_* in terms of O*_SB_* and O*_Sb_* as follows:


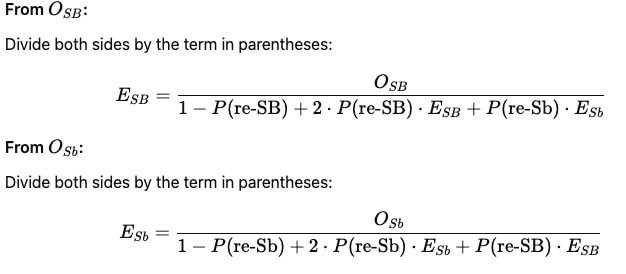


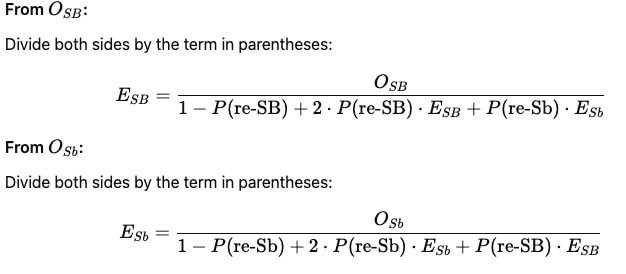


Next, we replace M*_SB_* and M*_Sb_* with E*_SB_* and E*_Sb_* in equations 1–5 of Part 1, and search for the best estimates of P_(re-_*_SB_*_)_ and P_(re-_*_Sb_*_)_. In this model, the best estimates are close to the results from the base model in part 1: for the Georgia population, P_(re-_*_Sb_*_)_ = 0.4208, P_(re-_*_SB_*_)_ < 0.001; for the Florida population, P_(re-_*_Sb_*_)_ = 0.6432, P_(re-_*_SB_*_)_ < 0.001. When combining data from both populations, the best estimates are: P_(re-_*_Sb_*_)_ = 0.5711, P_(re-_*_SB_*_)_ < 0.001.

Method S9: Differences in attributes of polyandrous and monandrous queens

We investigated whether polyandrous polygyne queens differed from monandrous ones for any of the ancillary data recorded for our *GA-2023* dataset. First, the two types of queens were compared with respect to their relative production of worker or gyne daughters. Proportionate success in maternity of pupae of each caste relative to her nestmates was tallied for each queen at each sampling point, and the values for each group (polyandrous, monandrous) were compared after pooling all colonies and samples by using 1-tail bootstrap difference tests (5000 iterations); differences significantly greater than zero would suggest greater success of polyandrous than monandrous queens with respect to their maternity shares for daughters of a given caste. Because no differences were found using this rough method of comparison that does not account for non-independence of data or for colony effects, we judged that more complex analyses were not warranted.

For comparison of weights and fecundity, similar preliminary analyses using mean values for monandrous queens in each colony at each sample point did suggest possible differences between the two types of queens, so resampling/bootstrap difference tests were implemented that accounted for repeated sampling and colony effects while making full use of the raw data. For each attribute, the difference between a single polyandrous queen and a randomly chosen monandrous nestmate queen was calculated for a single, randomly selected sampling point for each of the five colonies containing polyandrous queens; thus, five difference values were obtained for each iteration. The procedure was performed 100 times, yielding 500 difference values that were then bootstrapped 5000 times to provide the mean differences and the probabilities that these means were greater than zero. These were taken as the probabilities that polyandrous queens weigh more and are more fecund than monandrous queens.

Method S10: Nestmate progeny relatedness in the monogyne form

The genetic relatedness of monogyne nestmate gynes (*r*_p(g)_) was calculated from data extracted from Ross and Shoemaker (1997) using the individual pairwise MOM estimator of Huang et al. (2014). The data were genotypes at seven allozyme loci for 799 progeny gynes from 96 monogyne nests in northeastern Georgia, U.S. The overall mean and 95% confidence interval were based on 5000 bootstrap resamplings of the single-colony estimates.

Method S11: Nestmate progeny relatedness in the polygyne form in the wild

We calculated the average relatedness of the worker progeny (*r*_p(w)_) and gyne progeny (*r*_p(g)_) in 28 confirmed polygyne colonies from the allozyme data of Ross (1993) obtained for a wild population in northern Georgia, using the Huang et al. (2014) MOM estimator and combining life stages (larvae [workers only], pupae, adults) for each caste. The estimates are *r*_p(w)_=0.086 (95% CI 0.049-0.128; *n*=3073 individuals; eight loci) and *r*_p(g)_=0.103 (0.036-0.172; *n*=1926 individuals; six loci). Although the difference between the two average estimates is in the expected direction given that fewer effective numbers of mothers of gynes than of workers were estimated (see Fig. 6a, c), the difference is not statistically significant (*P*=0.26; one-tail bootstrap difference in means for the castes in each colony; 1000 iterations).

# Supplemental Information – Tables

Table S1. Diversity metrics, quality control statistics, and genomic locations for 13 microsatellite loci in *S. invicta* from northern Georgia

| Locus | Count of alleles observed | Expected heterozygosity (*H*_exp_)† | Estimated probability of HWE (*H*_obs_=*H*_exp_)‡ | Estimated probability of null allele§ | Estimated genotyping failure rate§ | Chromosome |
| --- | --- | --- | --- | --- | --- | --- |
| *Bertha* | 7 | 0.643 | 0.682 | 0 | 0 | 3 |
| *C27* | 3 | 0.328 | 0.192 | 0.018 | 0.011 | 6 |
| *C294*^¶^ | 6 | 0.696 | 0 | ― | ― | 16 |
| *C536* | 9 | 0.809 | 0.456 | 0.001 | 0 | 6 |
| *Cassidy* | 5 | 0.654 | 0.865 | 0 | 0.012 | 7 |
| *i_109* | 6 | 0.729 | 0.963 | 0 | 0 | 14 |
| *i_114* | 7 | 0.716 | 0.077 | 0 | 0 | 5 |
| *i_120* | 7 | 0.707 | 0.511 | 0 | 0.017 | 10 |
| *i_126*^¶^ | 8 | 0.749 | <0.001 | ― | ― | 16 |
| *i_129* | 5 | 0.323 | 0.413 | 0 | 0.075 | 4 |
| *Sol_42f* | 16 | 0.77 | 0.388 | 0.001 | 0.012 | 15 |
| *Sol_49* | 8 | 0.753 | 0.907 | 0 | 0.012 | 8 |
| *Sunrise* | 5 | 0.498 | 0.558 | 0 | 0.006 | 14 |
| † based on *n*=175 wingless reproductive polygyne queens; this is the proportion of heterozygotes expected conditional on observed allele counts under Hardy-Weinberg equilibrium (HWE) with Levene's (1949) correction | | | | | | |
| ‡ based on *n*=175 wingless reproductive polygyne queens using methods of Guo and Thompson (1992) and Rousset and Raymond (1995) | | | | | | |
| § based on *n*=9287 worker and gyne pupae using methods of Kalinowski and Taper (2006) | | | | | | |
| ¶ loci located on Chr16; these are in strong (*i_126*) or complete (*C294*) linkage disequilibrium with the *Social* *supergene* located on the distal arm of Chr16 (Ross & Shoemaker, 2018) | | | | | | |

| Table S2. Estimates of *M*_e_ (genetically effective number of mates per queen) and *F*_IS_ (inbreeding coefficient) for monogyne *S. invicta* from the southern U.S. All values were generated from newly aggregated and/or unpublished data from listed source. | | | | | |
| --- | --- | --- | --- | --- | --- |
|  | **Sampling locale(s)** | **Sample sizes** | |  | **Data source** |
| ***M*_e_** |  | **individuals** | **nests** | **Markers** |  |
| **1.04^†^**  (1.00-1.14) | northern Georgia | 799 young adult gynes (unmated) | 96 | 7 allozyme loci | Ross and Shoemaker (1997) |
| **1.01^‡^**  (1.00-1.04) | northern Florida | 92 mated reproductive queens | 92 | 11 msat loci | Lawson et al. (2012) |
|  |  |  |  |  |  |
| ***F*_IS_** |  |  |  |  |  |
| **0.047^§^**  (0.026-0.067) | Texas, Louisiana (2 sites), Mississippi, Georgia, Florida | 503 young adult gynes | 503 | 7 msat and  9 allozyme loci | Shoemaker et al. (2006) |
| **0.034^¶^**  (0.024-0.045) | northern Georgia | 559 newly mated queens | queens captured on ground | 99 msat and  6 allozyme loci | Fisher (2013) |
| **0.007^§^**  (-0.073-0.083) | northern Georgia | 61 young adult gynes | 61 | 12 msat loci | Ascunce et al. (2011) |
| 95% confidence intervals in parentheses obtained using 1000-5000 bootstrap replicates over loci, nests, or individuals; msat, microsatellite | | | | | |
| ^†^Estimated indirectly based on *r*_p(g)_ estimate of 0.730 (main text; Method S10) and assumptions that *r*_sp_=0.75 and *r*_dp_=0.25 using Equation (4) in Ross (2001) | | | | | |
| ^‡^Estimated directly from spermathecal contents of queens, and assuming paternity skew estimated for polyandrous polygyne queens in current report, using Equation (6) in Ross (2001) as amended in footnote to Box 3 in that article | | | | | |
| ^§^ Calculated using method of Weir and Cockerham (1984); *F*_IS_ values close to zero signify no inbreeding | | | | | |
| ^¶^ Calculated using Equation (7.7c) of Hedrick (2005) | | | | | |

| **Table S3. Numbers of wingless, presumed reproductive queens per nest in polygyne *S. invicta* in the invasive (U.S.) range.**  Statistics are given separately for all queens or only mated queens. | | | | | | | | | | |  |
| --- | --- | --- | --- | --- | --- | --- | --- | --- | --- | --- | --- |
|  |  |  | **All dealate queens count** | | |  | **Mated dealate queens count** | | | | |
| **Reference** | **Locale (collection year)** | **Number of nests** | **Range** | **Arithmetic mean (±SD)** | **Harmonic mean** |  | **Range** | **Arithmetic mean (±SD)** | **Harmonic mean (±SD)** | **Proportion mated (±SD)** | |
| Lofgren et al. (1975) | Jackson Co., MS (1974) | 10 | 7 – 677 | > 300 | ― |  | ― | ― | ― | ― | |
| Vargo & Fletcher (1987) | Walton Co., GA (1986) | 20 | 25 - 452 | 103.7 ± 105.1 | ― |  | ― | ― | ― | 0.74 ± 0.14 | |
| Vargo & Fletcher (1989) | Travis Co., TX (1989) | 12 | 5 – 186 | 70.9 ± 54 | ― |  | ― | ― | ― | 0.70 ± 0.19 | |
| Goodisman & Ross (1996) | Walton Co., GA (1990) | 35 | 2 – 143 | 32.3 ± 32.2 | 15.27 |  | ― | ― | ― | ― | |
| Goodisman & Ross (1998) | Walton Co., GA (1997) | 85 | 2 – 257 | 30.56 | ― |  | ― | ― | ― | 0.49 | |
| This report | Clarke Co., GA (2024) | 8 | 5 – 72 | 40.88 ± 21.48 | 21­.48 |  | ― | ― | ― | ― | |
| Fletcher et al. (1980) | Jackson Co., MS (1980) | 15 | ― | ― | ― |  | 3 – 56 | 27.67±16.85 | 13.82 | ― | |
| Ross et al. (1996) | Walton Co., GA (1985) | 26 | ― | ― | ― |  | ― | 33.8 ± 8.2 | 8.74 ± 3.95 | ― | |
| Ross et al. (1996) | Travis Co., TX (1987) | 33 | ― | ― | ― |  | ― | 20 ± 7.7 | 5.67 ± 3.06 | ― | |
| Ross et al. (1996) | Walton Co., GA (1990) | 27 | ― | ― | ― |  | ― | 28.3 ± 10.5 | 9.54 ± 3.86 | 0.52 – 0.95 | |
| Goodisman & Ross (1997) | Walton Co., GA (1990 & 1994) | 63 | 2 – >50 | 30.49 ± 7.79 | ― |  | ― | 21.56 ± 5.89 | ― | 0.71 | |
| Ross (1993) | Walton Co., GA (1993) | 31 | ― | ― | ― |  | 1 – 92 | 24.81 ± 27.36 | 4.54 | ― | |
| Ross et al. (1996) | Travis Co., TX (1995) | 20 | ― | ― | ― |  | ― | 35.4 ± 18.5 | 10.4 ± 7.2 | ― | |
| ― Data not available | | | | | | | | | | |  |

**Table S4. Estimates of *F*_IS_ (inbreeding coefficient) for polygyne *S. invicta* from various sites in the southern U.S.** All values were generated from newly aggregated and/or unpublished genotypic data for ten or more markers from the listed sources.

|  | Sampling locale(s) | Sample sizes | |  | Data source |
| --- | --- | --- | --- | --- | --- |
| ***F*_IS_** |  | **individuals** | **nests** | **Markers** |  |
| **0.045**  (0.017-0.073) | Texas, Louisiana (2 sites), Mississippi, Georgia, Florida | 557 adult females | 557 | 7 msat and  8 allozyme loci | Shoemaker et al. (2006) |
| **-0.016**  (-0.046-0.016) | northern Georgia | 3073 workers | 31 | 10 allozyme loci | Ross (1993) |
| **-0.011**  (-0.030-0.008) | northern Georgia | 175 reproductive queens | 22 | 11 msat loci | this report |
| *F*_IS_ was calculated using the method of Weir and Cockerham (1984); 95% confidence intervals in parentheses were obtained using 1000-5000 bootstrap replicates over loci or nests; *F*_IS_ values close to zero signify no inbreeding; msat, microsatellite | | | | | |

| Table S5. Estimates of three metrics for colony queen number in polygyne *S. invicta* in the field (northern Georgia, U.S.). Values are shown for all nests, the subset of nests with greater than ten queens, and the subset with >40 queens. The expected ordering of values of the three metrics absent high queen turnover (*Q*_e_ < *Q*_h_ < *Q*_x̅_) is most evident when nests with very few queens are excluded from the analysis, as the ranges for the difference in harmonic mean and genetically effective mean numbers are otherwise relatively compressed. | | | | |
| --- | --- | --- | --- | --- |
| Counted nest-mate queens | No. nests^†^ | *Q*_x̅_  (arithmetic mean no.) | *Q*_h_  (harmonic mean no.)^‡^ | *Q*_e_  (genetically effective no.)^§^ |
| 1-92 | 31 | 24.8 | 4.5 | 4.6 |
| >10 | 18 | 40.2 | 25.5 | 9.8 |
| >40 | 8 | 63.9 | 57.7 | 21.2 |
| Data from Ross (1993); *Q*_x̅_ and *Q*_h_ are estimated from direct counts of queens, while *Q*_e_ is estimated indirectly from progeny relatedness | | | | |
| **^†^** 31 nests sampled in entire population | | | | |
| **^‡^** takes into account variation in number of mated queens per nest | | | | |
| **^§^** takes into account variation in number of mated queens per nest and maternity skew; based indirectly on *r*_p(w)_ estimates from worker genotypes (larvae, pupae, and adults) at seven allozyme loci (Ross, 1993) and assuming *r*_dm_=0 and *r*_sm_=0.75, using equation (1) of Ross (2001) | | | | |

| Table S6. Estimates of *Q*_e_ (genetically effective colony queen number) for maternity of pupae of the two female castes in polygyne *S. invicta* in the field (northern Georgia). Values are shown for the entire collection of nests that produced pupae of both castes, the subset with greater than ten queens, and the subset with greater than 40 queens. | | | |
| --- | --- | --- | --- |
| Counted queens | No. nests^†^ | *Q*_e_ | |
|  |  | workers | gynes |
| 1-92 | 25 | 8.80 | 7.31 |
| >10 | 16 | 6.23 | 5.30 |
| >40 | 8 | 9.49 | 5.28 |
| Data from Ross (1993); *Q*_e_ estimated indirectly based on *r*_p(w)_ and *r*_p(g)_ estimates from seven allozyme loci and assuming *r*_sm_=0.75 and *r*_dm_=0 using equation (1) in Ross (2001) | | | |
| ^†^ 31 nests sampled in entire population, but only 25 produced gyne as well as worker pupae | | | |
|  | | | |

# Supplemental Information – Figures


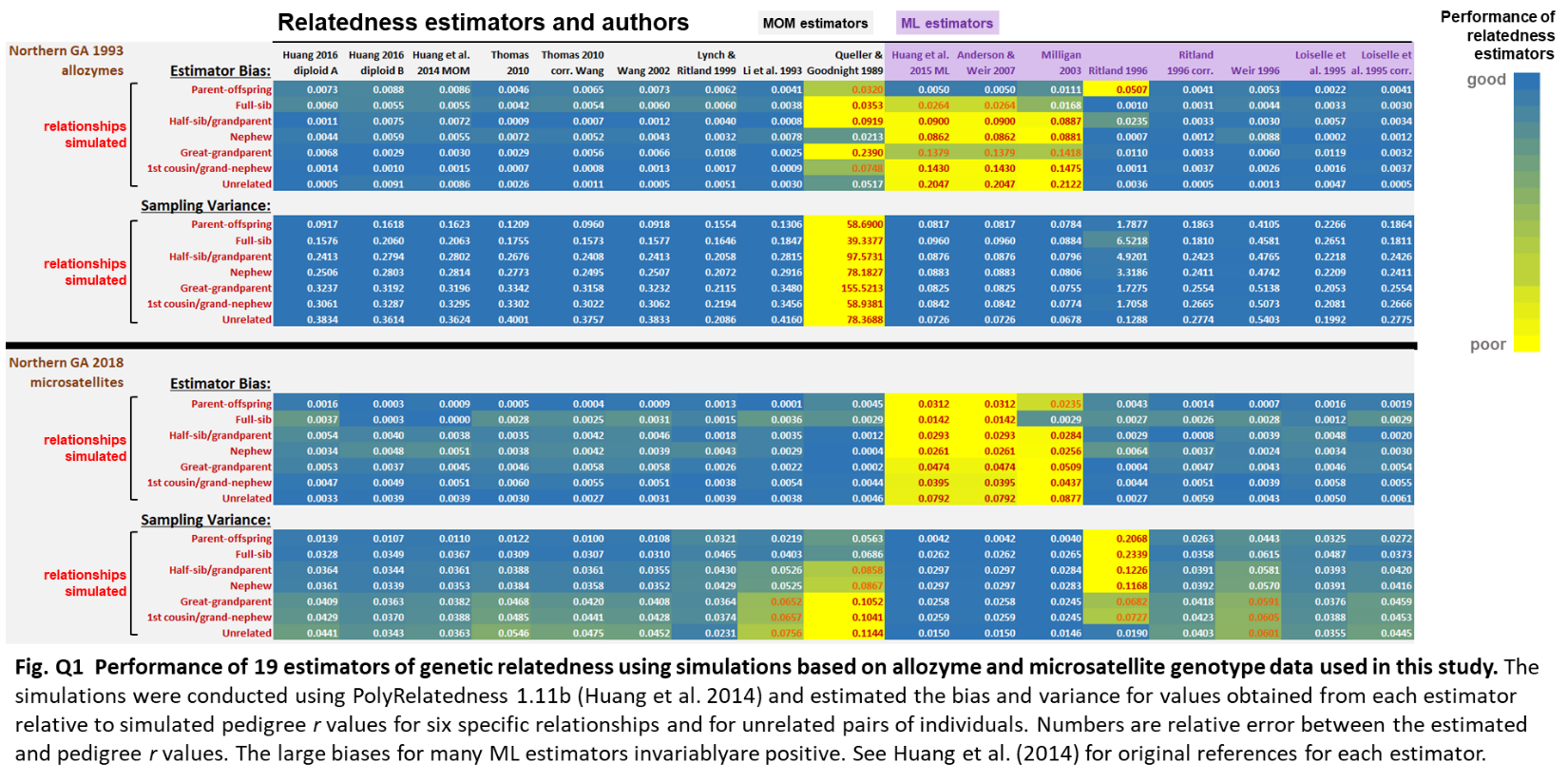


**Figure S1. Performance of 19 estimators of genetic relatedness using simulations based on allozyme and microsatellite genotype data used in this report.** The simulations were conducted using PolyRelatedness 1.11b (Huang et al., 2014) and estimated the bias and sampling variance for values obtained from each estimator relative to simulated pedigree *r* values for six specific relationships and for unrelated pairs of individuals. Method of moments (MOM) estimators are on the left and maximum likelihood (ML) estimators are on the right. Numbers are relative error between estimated and actual values. The large biases for many ML estimators are upward. See Huang et al. (2014) for original references for each estimator.


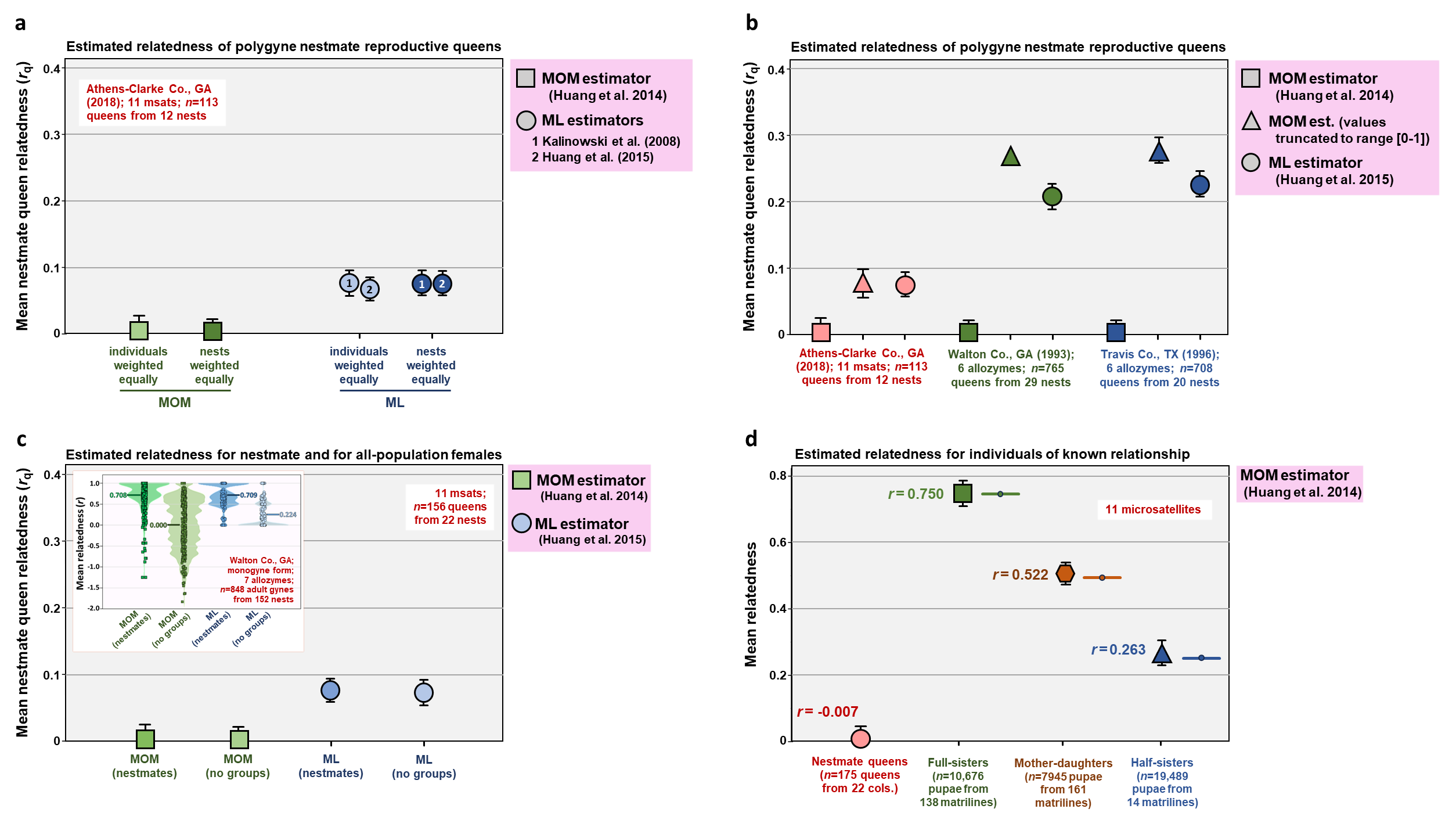


**Figure S2. Comparison of method of moments (MOM) and maximum likelihood (ML) estimators for estimating average pairwise genetic relatedness in invasive (U.S.) *S. invicta*.** Error bars represent 95% confidence intervals based on 5000 bootstrap replicates over nests (or, in a few instances, individuals). (a) Comparison of MOM and ML estimators of polygyne nestmate queen relatedness (*r*_q_) using genotypic data from microsatellites (msats) (*GA-2018* dataset) and employing two different weighting schemes; (b) Comparison of MOM (values truncated or not) and ML estimators of *r*_q_ using genotypic data from microsatellites (*GA-2018* data set) or allozymes (Walton County, Georgia data from Ross [1993]; Travis County, Texas data from Ross et al. [1996]); (c) comparison of MOM and ML estimators of polygyne female relatedness when specifying nestmates or ignoring group membership using data from microsatellites (combined *GA-2018* and *GA-2023* data sets); inset–same for monogyne females using allozymes (violin plots [with means]; data from Ross & Shoemaker [1997]); (d) comparison of MOM estimates of polygyne female relatedness (symbols) with expected pedigree values for pairs of known relationship (bars next to large symbols) and estimates for polygyne nestmate queens (using combined *GA-2018* and *GA-2023* data sets).


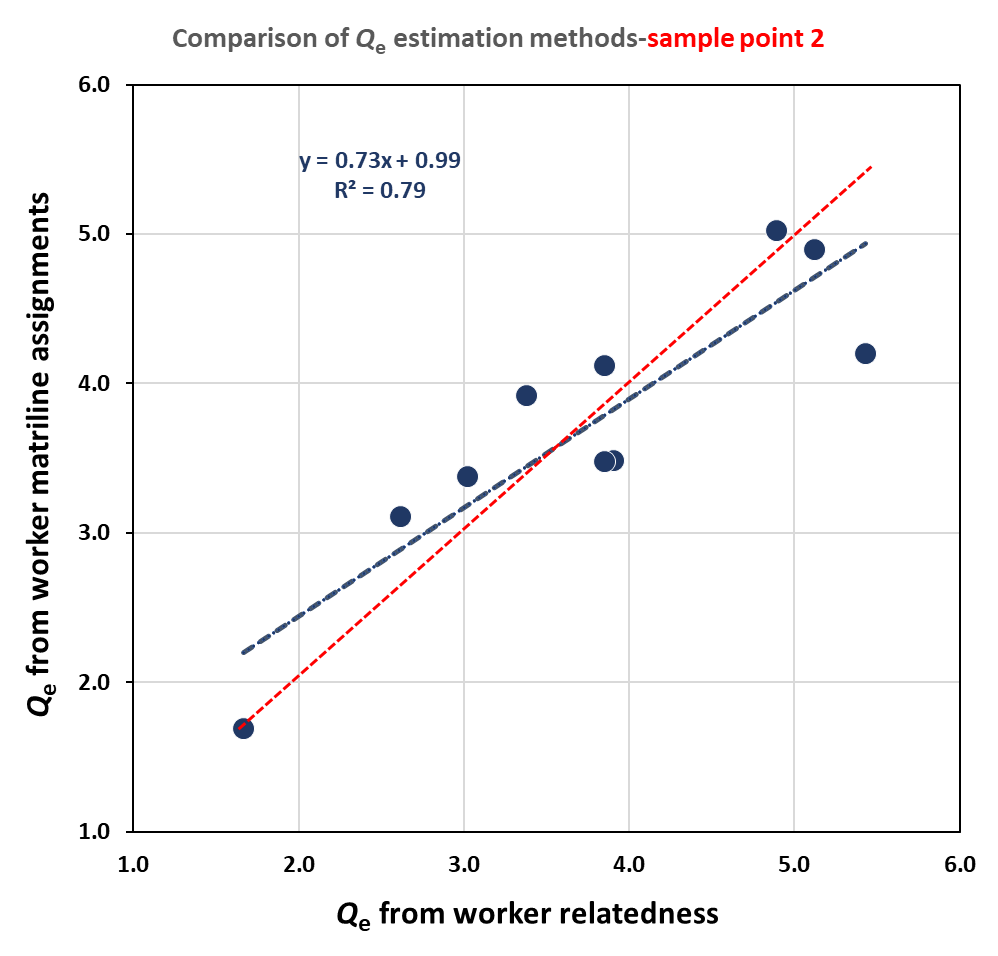


**Figure S3. Effective numbers of mother queens in polygyne *S. invicta* colonies (*Q*_e_) estimated using two different approaches.** Estimates for ten experimental colonies sampled at one timepoint (*GA-2023* dataset; sampling point 2) are based on microsatellite genotypes at 11 loci for 1347 worker pupae (see Method S1 and Method S3). On the x-axis are plotted the indirect estimates obtained by plugging the empirically derived values for average pairwise genetic relatedness of worker nestmates of different classes (*r*_p(w)_, *r*_sm_, and *r*_dm_) into Equation (1) in Ross (2001). On the y-axis are the direct estimates obtained from the known variance in maternity in each colony, as determined from parentage assignments, using Equation (4.20) in Crozier & Pamilo (1996). (Virtually identical results were obtained when Equation (16) in Nielsen et al. [2003] was used instead.) The least squares regression line (blue dashed) and its equation are shown along with the line showing complete concordance in estimates using the two approaches (red dotted).


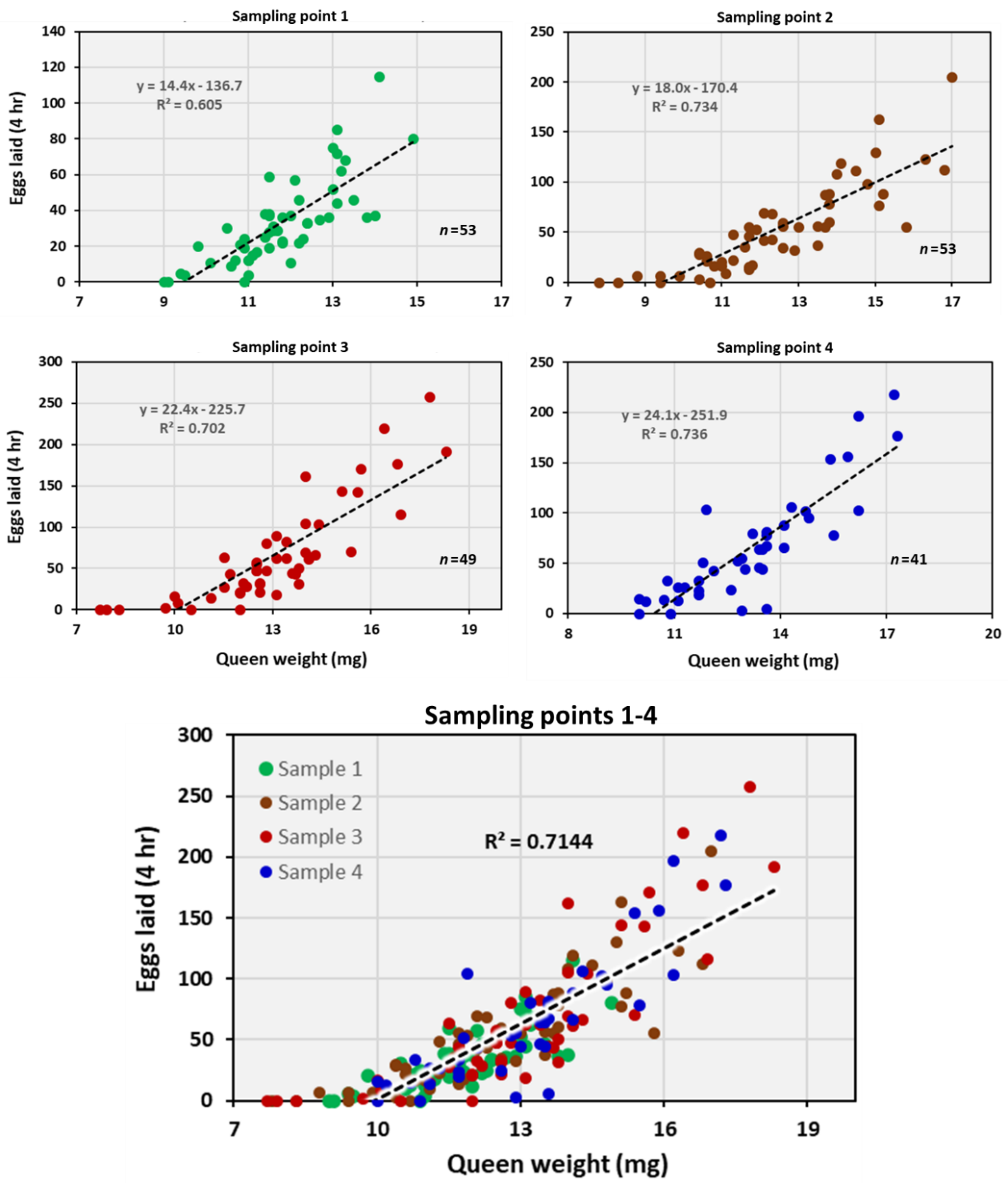


**Figure S4. Relationship between queen weight and fecundity in polygyne colonies.** All queens were mated egg-laying queens from ten colonies reared in the laboratory (*GA-2023* dataset); data are from the four primary sampling points covering the initial 6-7 months of the experiment. Fitted least squares regression line and equation are shown for each sampling time; queen weight typically explains almost three-quarters of the variance in fecundity. All correlations are highly significant (Spearman rank correlation tests; all *P*<0.001).


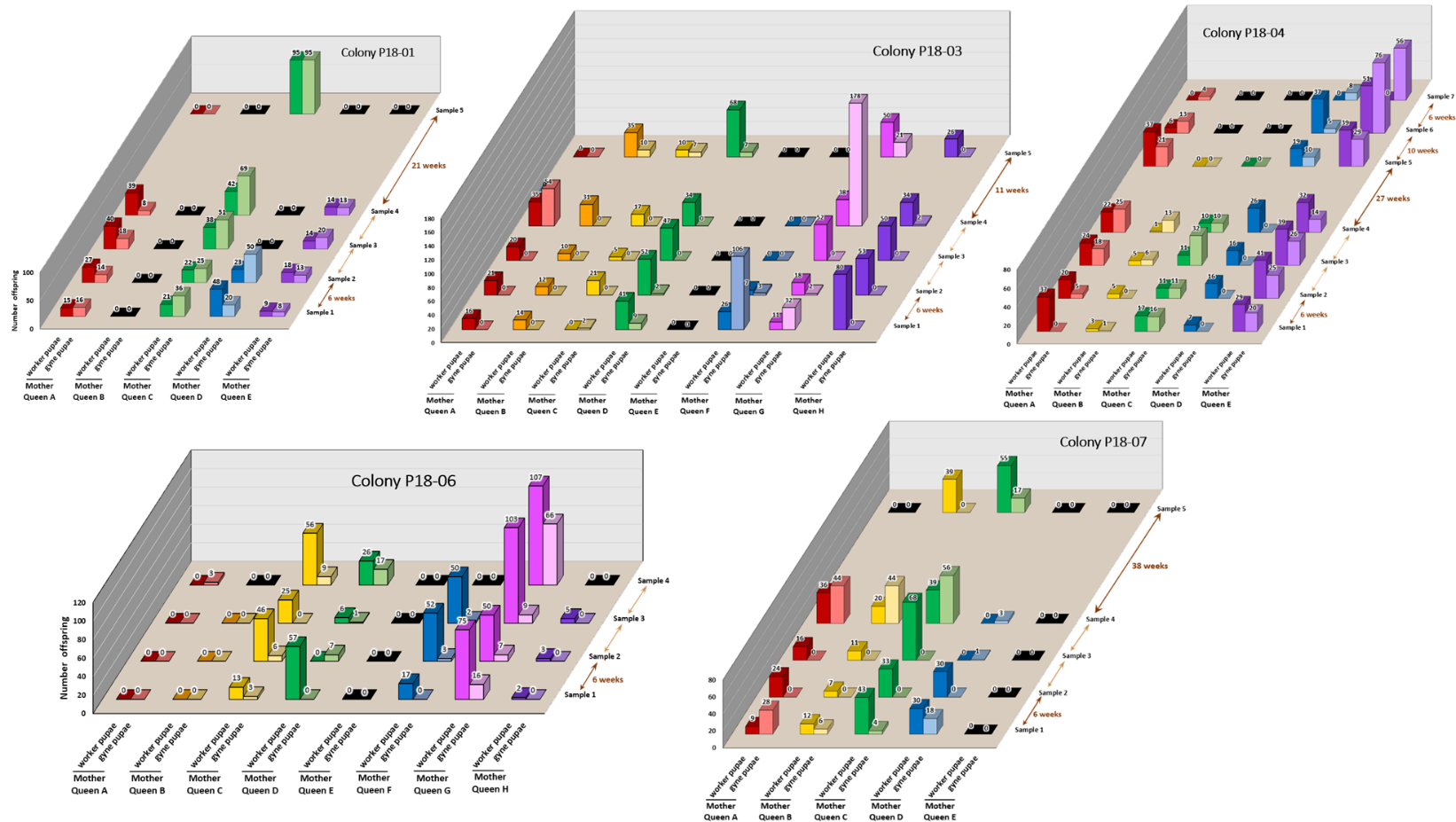
Figure S5

**
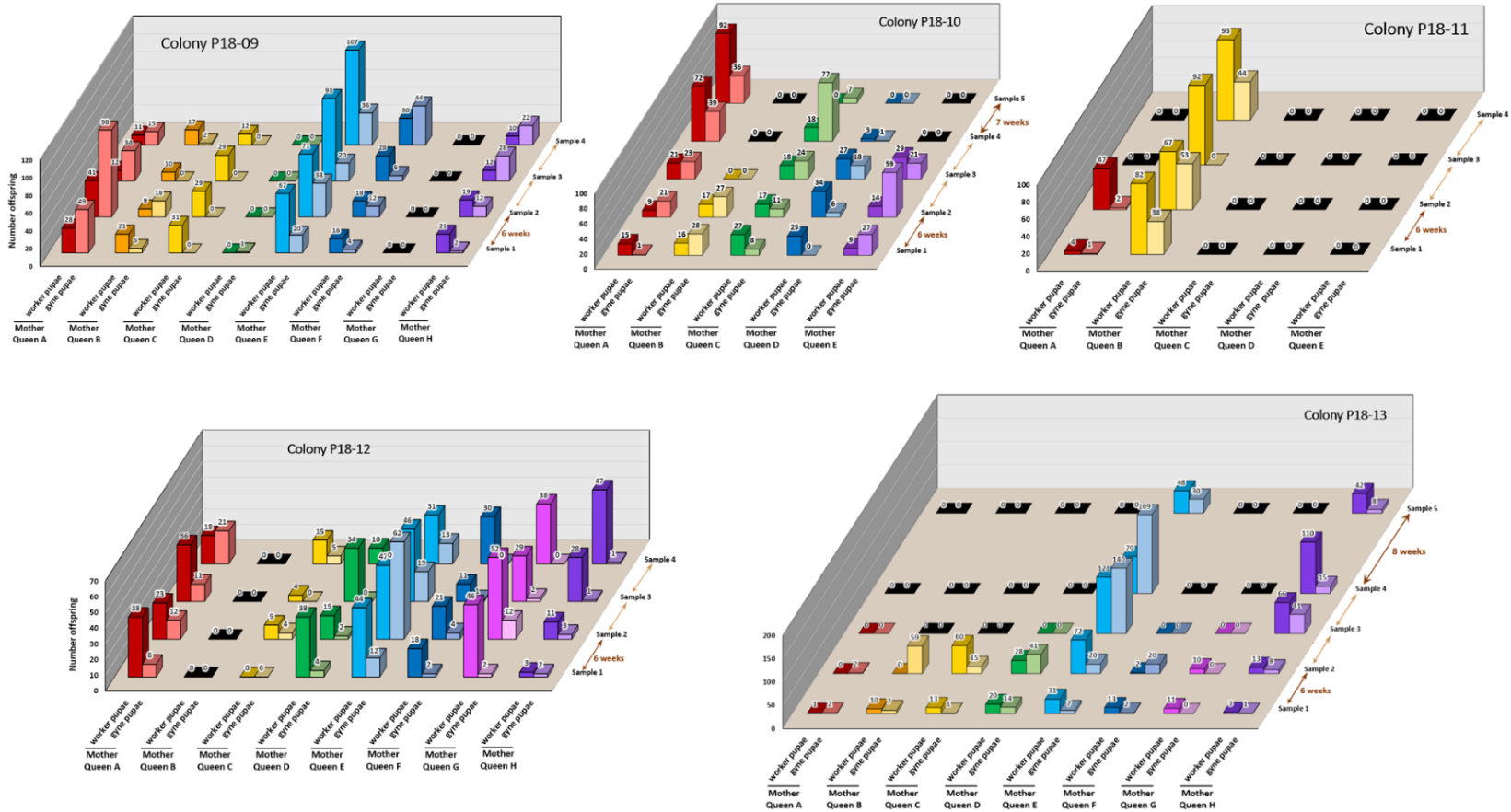
**Figure S5 (continued)

**Figure S5. Reproductive skew and temporal variance in maternity apportionment in ten semi-natural polygyne colonies (*GA-2023* dataset).** Columns show numbers of worker and gyne daughters produced by each queen alive at the previous sampling time (when eggs giving rise to the focal samples were laid). Data from each colony were obtained at 1-7 sampling points, the first four of which are the primary points. Unmated queens are indicated by continuous black tiles, while queens that died between sampling points are indicated by black tiles that appear after Sample 1.


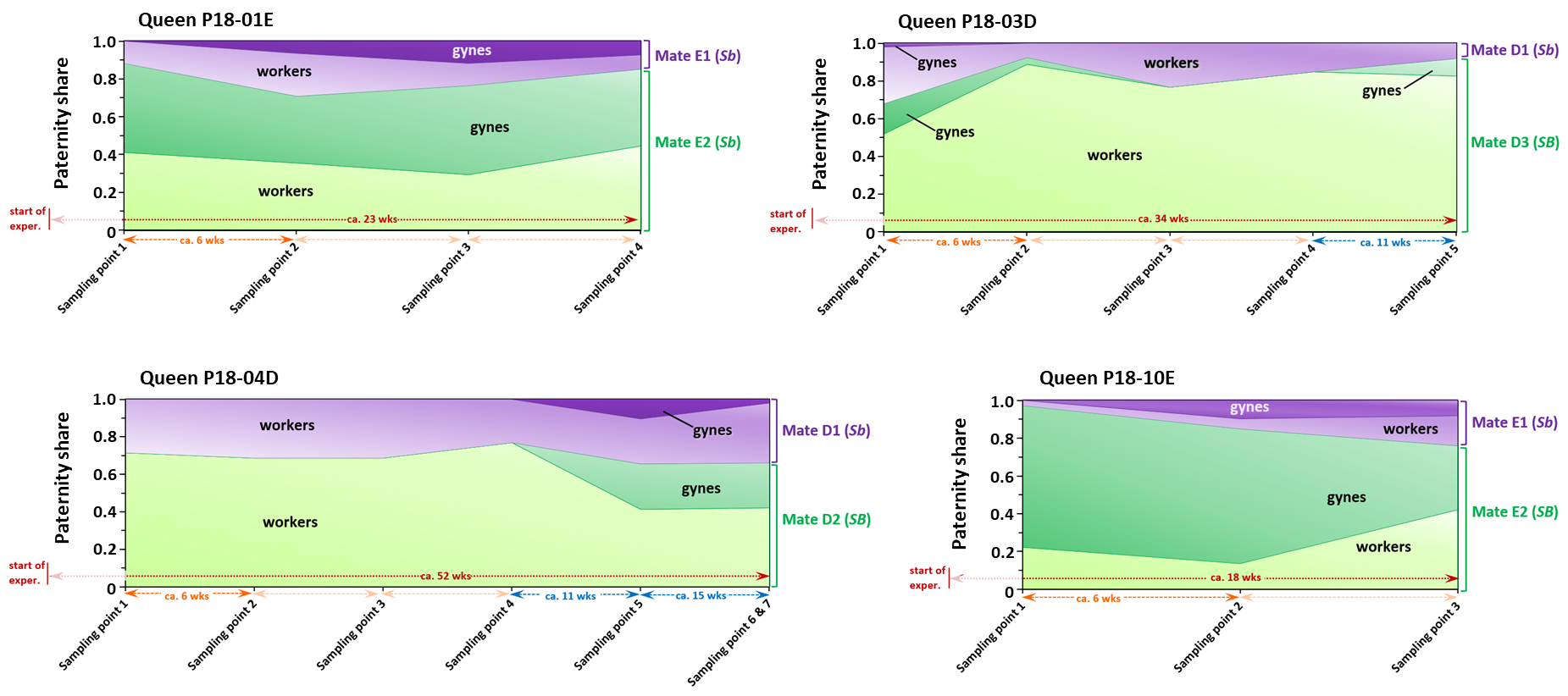
­­Figure S6


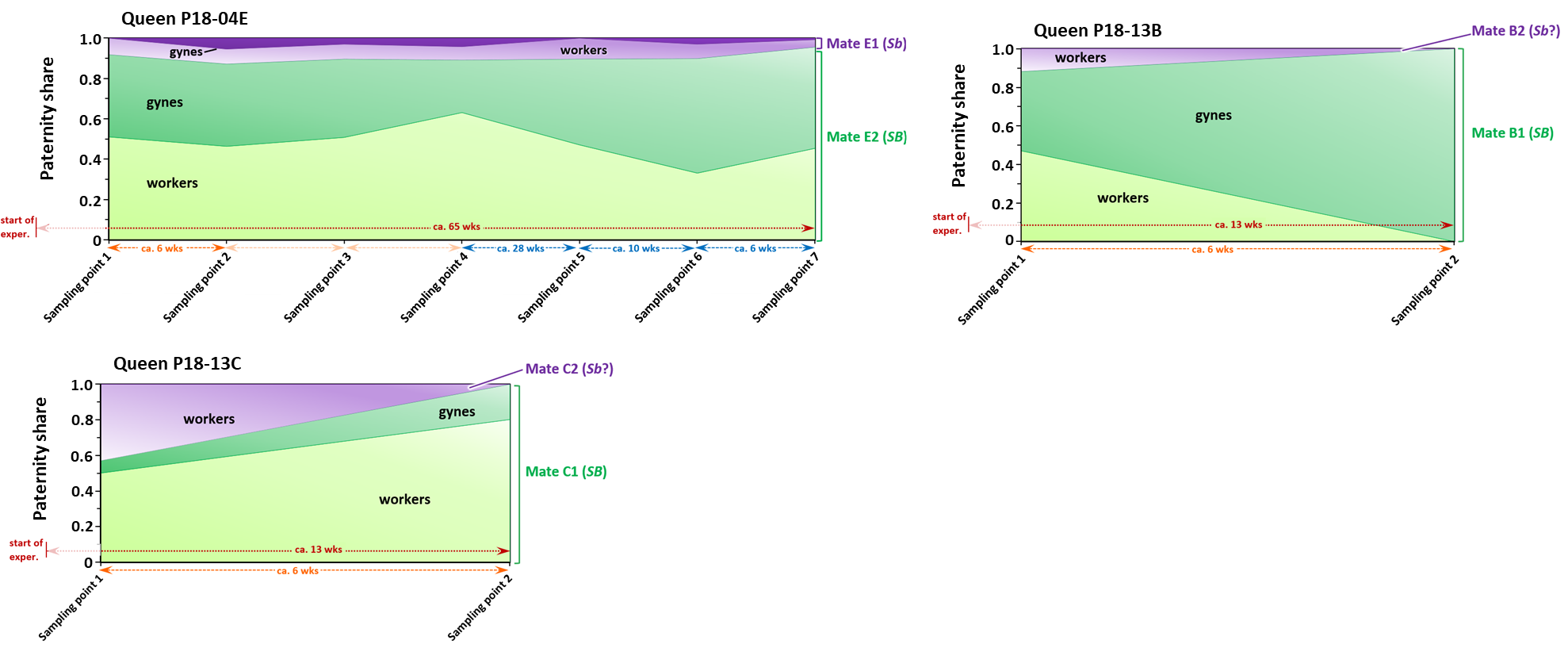
Figure S6 (continued)

**Figure S6.** **Paternity apportionment over time for twice-mated polyandrous queens from semi-natural polygyne colonies from northern Georgia.** The graphs show patrilines of female pupae of each caste (*GA-2023* dataset), with different patrilines distinguished by fundamental color. Gyne paternity is highlighted by darker shading and worker paternity by lighter shading of the fundamental color. Points on the x-axis are not scaled according to actual intervals between sampling times; these intervals are indicated on the arrows linking the points (arrows are unlabeled for intervals identical to previous one). The period from start of the experiment to the first sampling point was 6-7 weeks. See Fig. S13 for more information on paternity variance in each colony, including estimates of the *M*-index skew statistic.


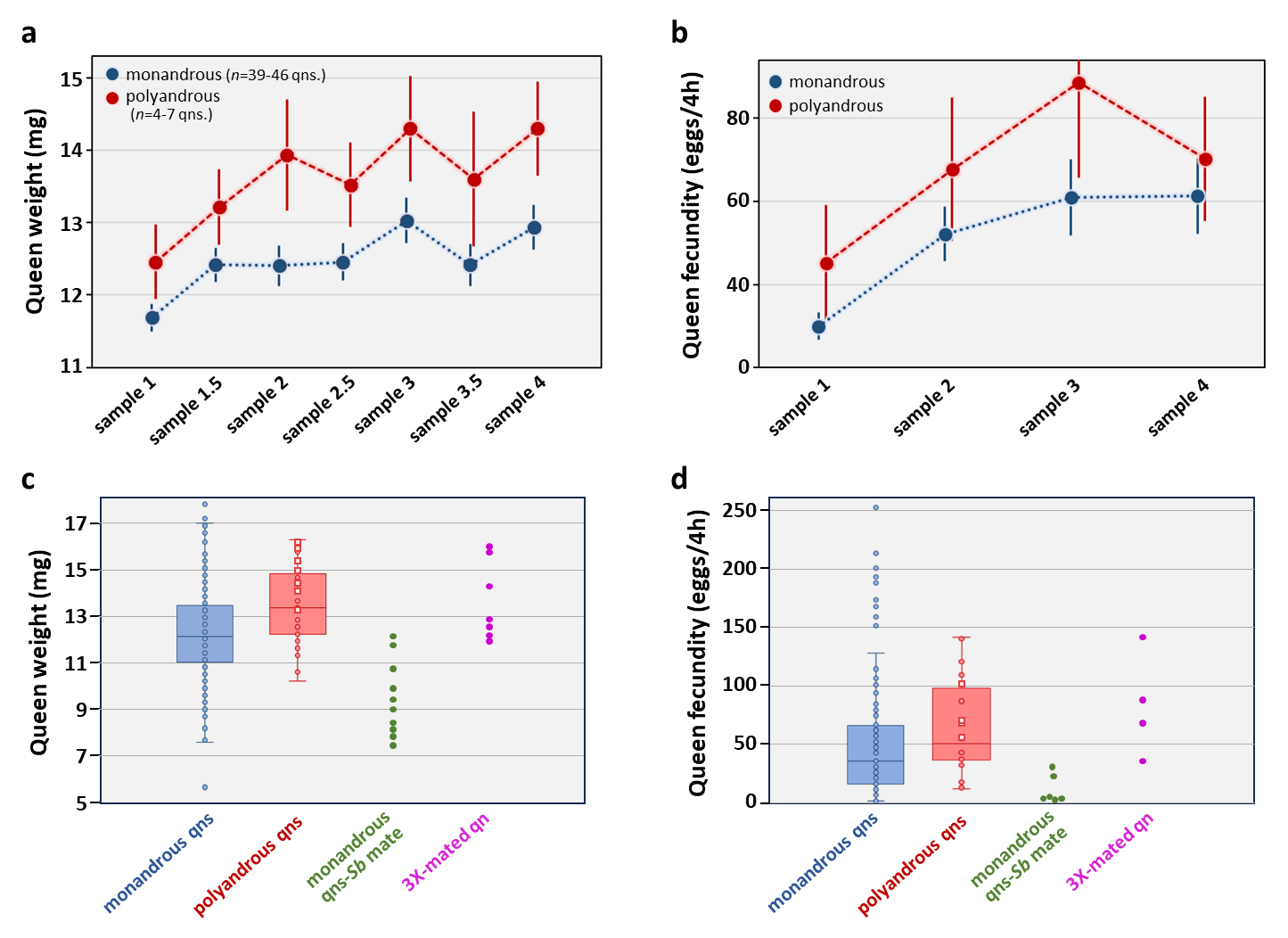


**Figure S8. Evidence for relationship between number of matings by a polygyne queen and her weight/fecundity later in life.** (a) Comparison between mean weights of monandrous and polyandrous queens at seven sampling points over a six-month period. The differences are statistically significant at each of the seven points (all *P*<0.0005, sequential resampling/bootstrap difference tests, 5000 iterations). Bars depict ±1 SE. (b) Comparison between mean fecundity of monandrous and polyandrous queens at four sampling points over a six month period. The differences are statistically significant at the first three individual points (all *P*<0.0005) but not at the fourth (*P*=0.279). (c) Comparison between weights of monandrous and polyandrous queens considered over the entirety of the experiment (data from each sampling point shown for each queen). The difference between all monandrous and all polyandrous queen/sample values is highly significant (*P*<0.001, bootstrap difference test using independent samples, 5000 iterations). Values for two monandrous queens that mated with an *Sb* male and for a single queen that mated with three males (1 *SB*, 2 *Sb*) are shown separately, while values for the sole polyandrous queen that mated with two *Sb* males are highlighted as white rectangles in the “polyandrous” box plot for comparison. Lines in box plots represent medians. (d) Comparison between fecundity of monandrous and polyandrous queens considered over the entirety of the experiment. The difference between all monandrous and all polyandrous queen/sample values is statistically significant (*P*=0.035, bootstrap difference test using independent samples, 5000 iterations). Categories and white rectangles in the “polyandrous” box plot are as in (c). All data from *GA-2023* dataset.


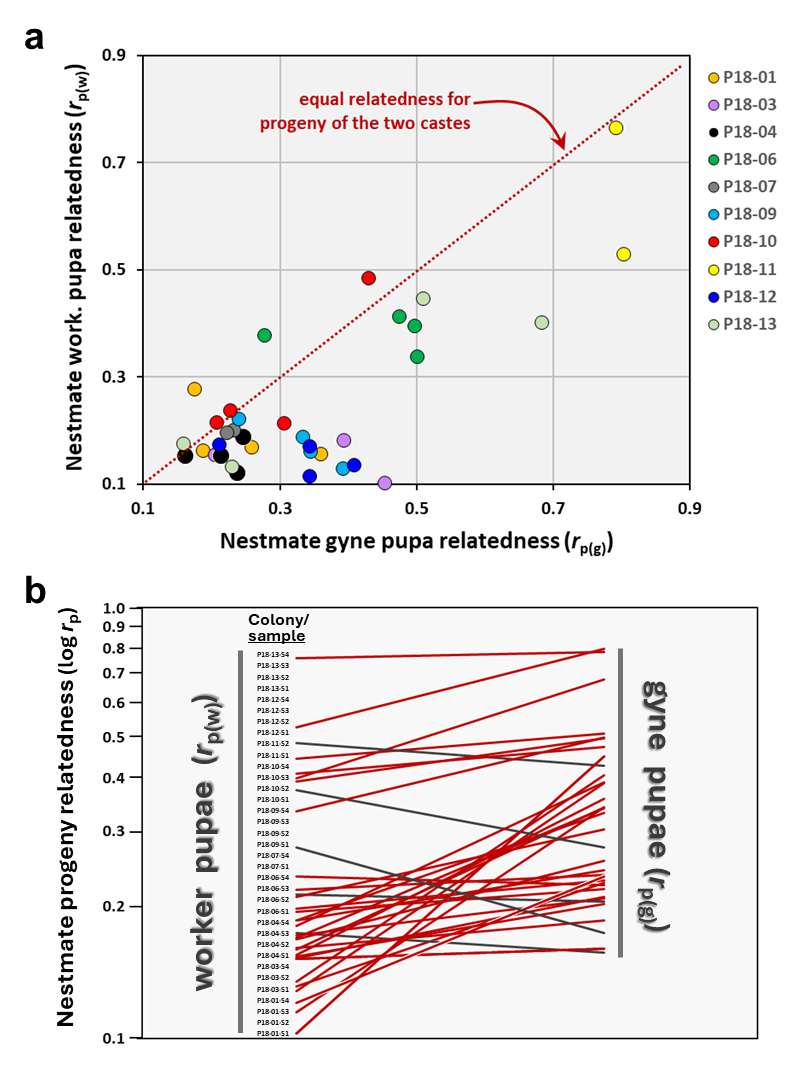


**Figure S9. Comparison of relatedness of nestmate offspring pupae of each caste.** (a) Worker relatedness plotted against gyne relatedness at 2-4 sampling points for each of ten colonies (35 total; *GA-2023* dataset). The binomial probability of 29 or more of the 35 points falling below the line assuming a 50:50 distribution is: *P*=0.00015. (b) Same data plotted on log scale; black lines indicate reversals of the common pattern of higher relatedness for nestmate gynes than workers from the same colony and sampling point. Because the number of mothers was known throughout the experiment, such elevated gyne relatedness at single sampling points necessarily reflects higher maternity skew and lower effective queen numbers for daughters of this caste compared to worker daughters.


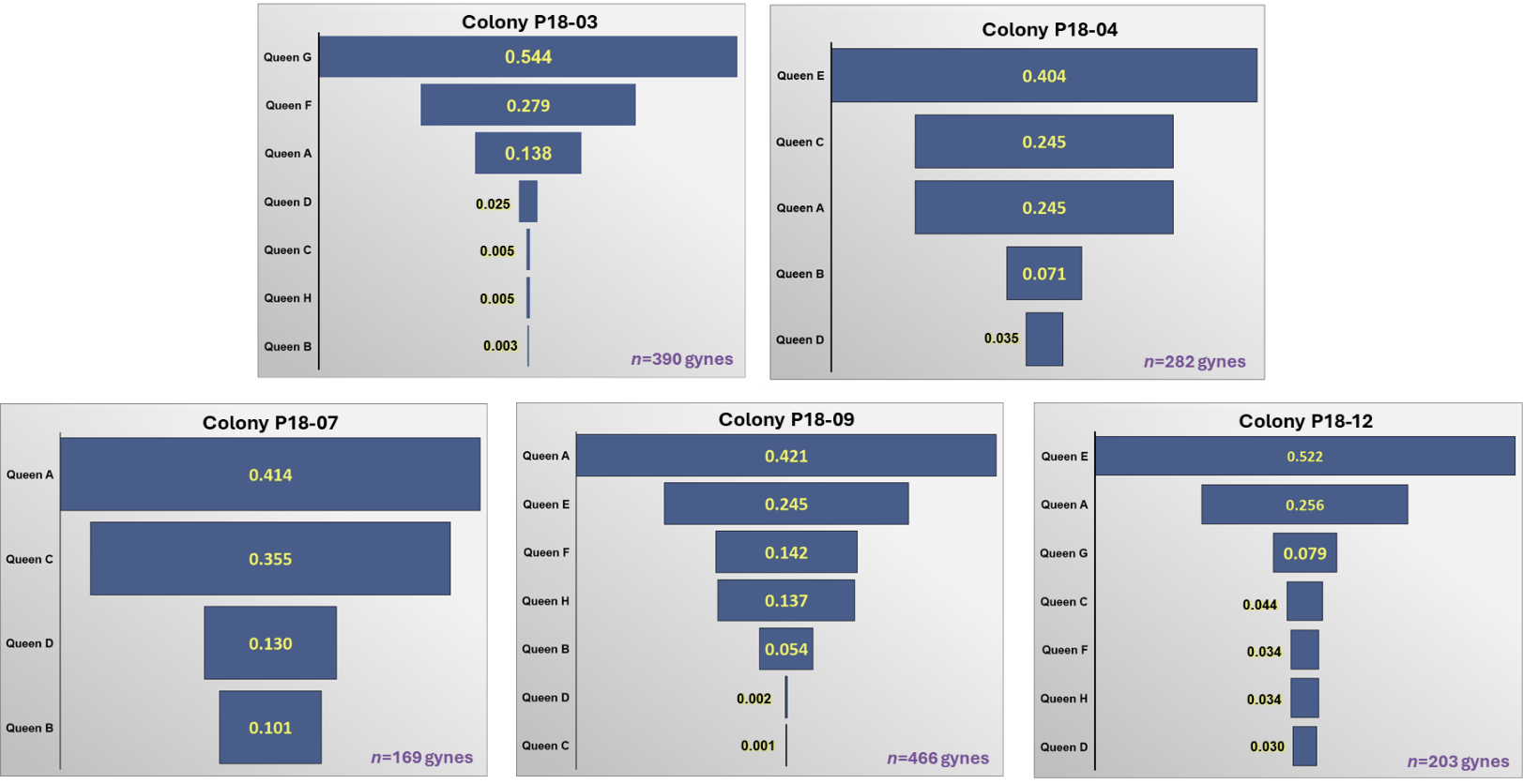


**Figure S10. Proportions of individual queens' daughters that were gynes relative to colony total gyne output.** Patterns were assessed for five colonies from the *GA-2023* dataset in which all queens survived the entire 6-month period spanning the four primary samples or, in the case of P18-04, these primary samples plus a fifth sample taken nine months into the experiment. Because of the short lifespan of polygyne queens and length of the experiment, these results may approximate “lifetime reproductive success”.


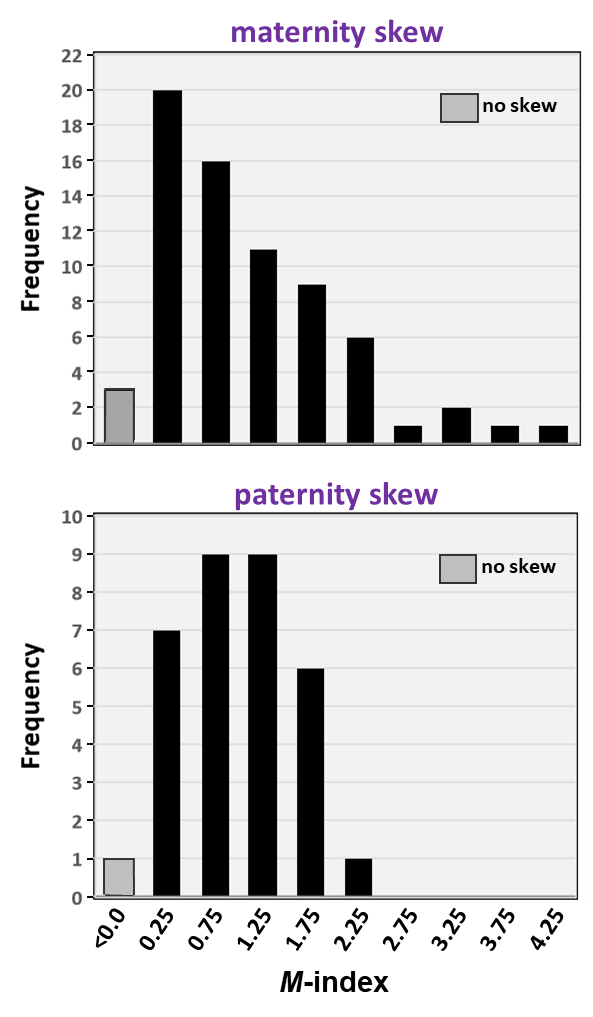
­­

**Figure S11. Distributions of *M*-index scores for skew for queens (mothers) and the male mates of polyandrous queens (fathers).** Data are from the combined *GA-2018* and *GA-2023* datasets (*N*=70 sampling instances for maternity skew and *N*=33 for paternity skew). All daughters (both castes) are included from the *GA-2023* dataset. Midpoint values for each bin are shown on the x-axis. The two distributions do not differ significantly (Mann-Whitney U-test on values, *P* = 0.445, 1-tail), although the highest values are confined to maternity skew. Skew distributions for maternity and paternity in each caste considered separately (*GA-2023* dataset) also do not differ significantly (Mann-Whitney U-tests, both *P* > 0.075, 1-tail).


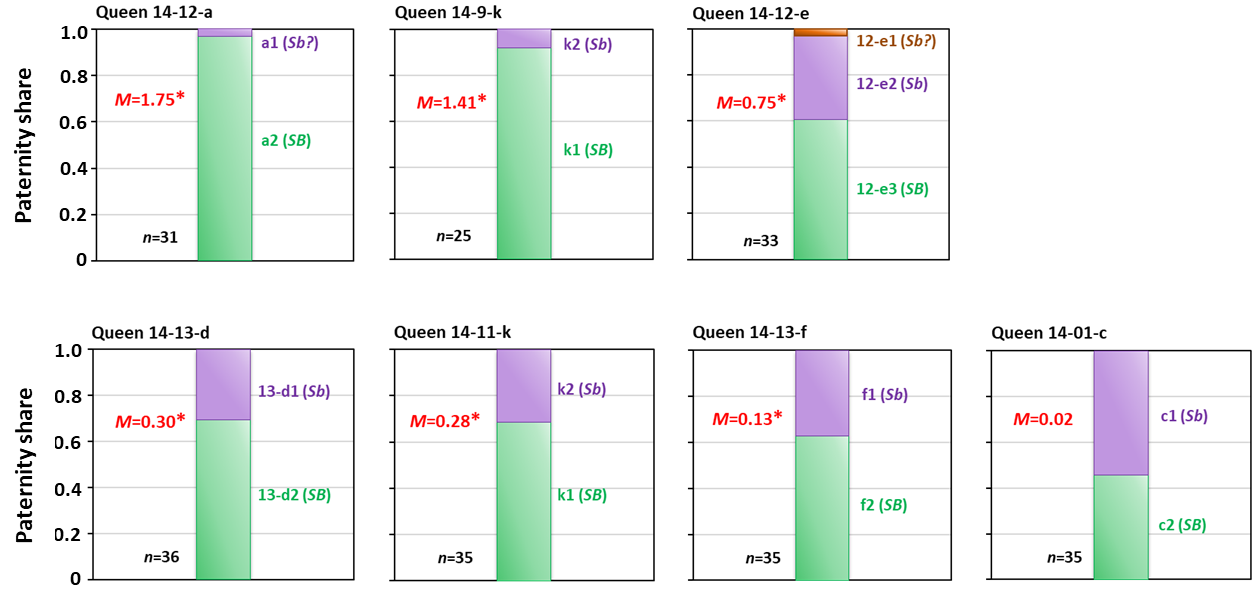


**Figure S12. Paternity apportionment for the male mates of seven polyandrous queens from laboratory polygyne colonies from northern Georgia.** The graphs show data for diploid embryo progenies (*GA-2018* dataset); identities of the mates and their supergene haplotypes are listed next to each column and the different patrilines are distinguished by color on the column. Values of the *M*-index skew statistic estimated for each queen are shown in red type; *indicates probabilities <0.05 that no skew exists (*M*=0) based on bootstrap calculations (1000 replicates) of the 95% confidence intervals when a 50:50 mix of patrilines and the actual sample size are assumed.


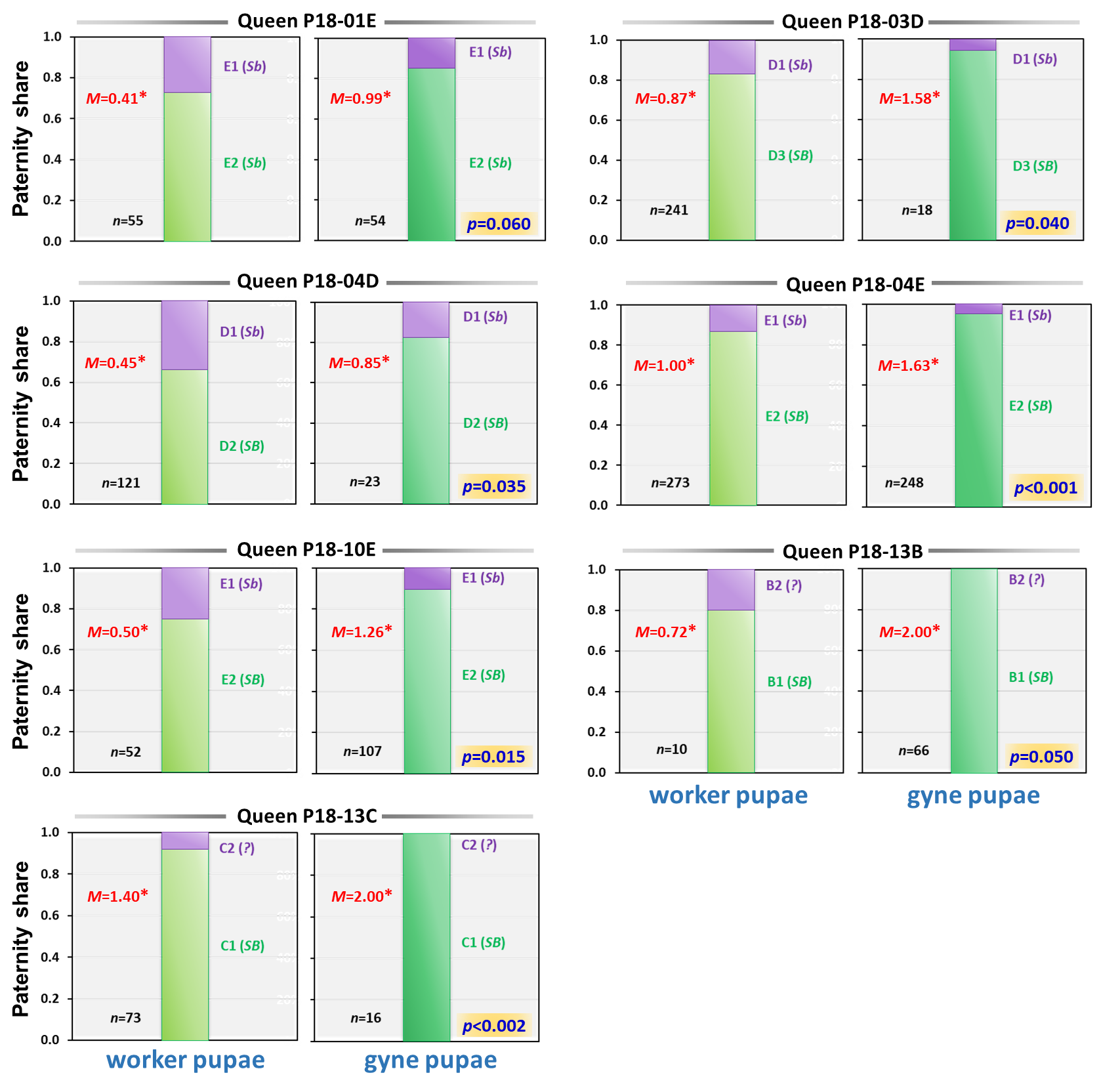


**Figure S13. Paternity apportionment for daughters of each caste for the male mates of seven polyandrous queens from laboratory polygyne colonies.** Samples were obtained at 2-7 time points spanning up to 65 weeks (the entire course of the *GA-2023* experiment), with the data for each queen pooled across samples (*n*=total sample size). Identities of the mates and their supergene haplotypes are listed next to each column and the different patrilines are distinguished by color on the column. Values of the *M*-index skew statistic estimated for each queen are shown in red type; *indicates probabilities <0.05 that no skew exists (*M*=0) based on bootstrap calculations (1000 replicates) of the 95% confidence intervals when a 50:50 mix of patrilines and the actual sample size are assumed. Probabilities of paternity apportionment not differing between the offspring castes are shown in blue type in each gyne panel (bootstrap differences test, 1-tail, 2000 iterations); note that Queens P18-04E and P18-10E have the lowest such probabilities among the five queens for which both male mate haplotypes were known, consistent with their complete lack of homozygous (*Sb/Sb*) gyne daughters (see Fig. 7b). Queen P18-01E is the only queen for which the probability is not significant (>0.05), an unsurprising outcome given that she mated with two *Sb* males.


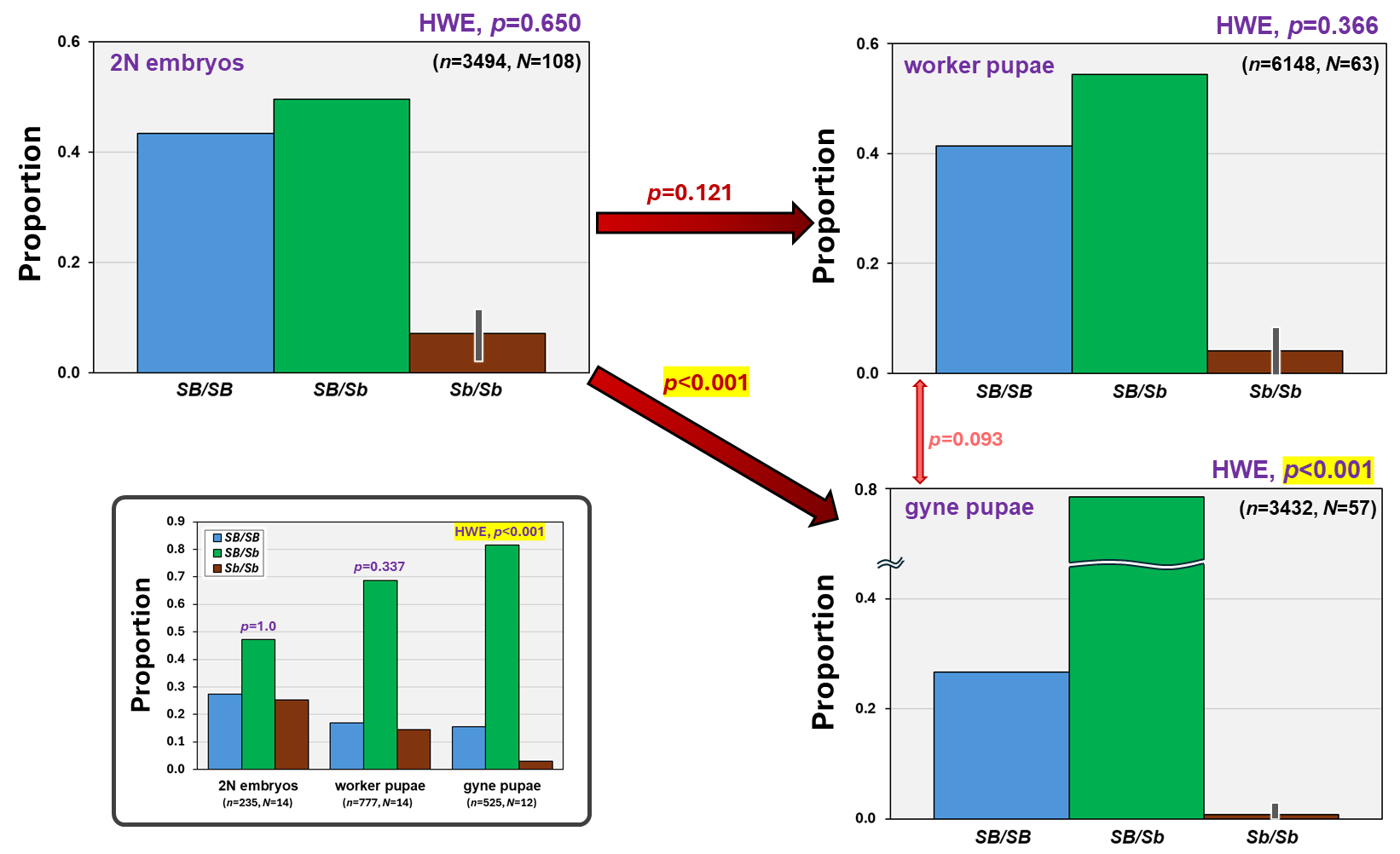


**Figure S14. Supergene genotype proportions in diploid embryos and female pupae of each caste produced by queens in laboratory polygyne colonies.**  *n*=number of individuals and *N*=number of matrilines/patrilines analyzed. The point estimates (and 95% confidence interval for the *bb* genotype [gray bars]), as well as the probabilities that the genotype distributions differ from each other or match Hardy-Weinberg expectations (HWE), were derived using a resampling approach (one individual sampled per patriline for 1000 iterations). Values by dark red arrows represent probabilities that the *bb* genotype was not less common in each class of pupae than in embryos. The value by the light red arrow is the probability that the *bb* genotype was not less common in gyne than worker pupae. Values in purple are probabilities that each distribution matches HWE. Embryo genotypes are from the *GA-2018* data set and pupal genotypes from the *GA-2023* data set. **Inset:** Data for polyandrous queens only. Loss of virtually all *Sb/Sb* gynes by the pupal stage causes pronounced departure of the genotypes in this group from HWE (probabilities of matching HWE in purple) and presumably explains some of the differential caste-specific paternity skew.


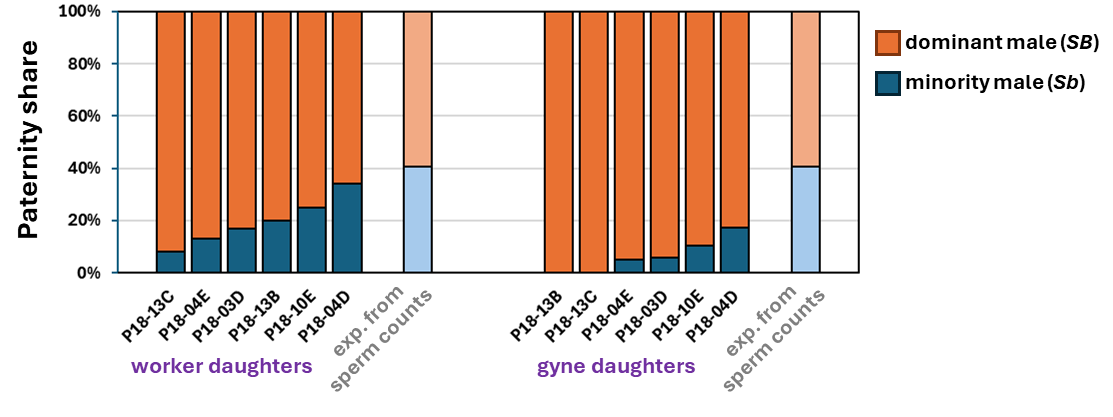


**Figure S15. Paternity apportionment and average sperm counts for *SB* and *Sb* male mates of twice-mated polygyne queens.** The dominant male in each case possessed the *SB* haplotype and had the greatest (or sole) share of paternity of offspring females; the minority male was assumed (P18-13,B and C) or confirmed (remaining four males) to have the *Sb* haplotype. Apportionment data are from the *GA-2023* dataset. Expected sperm count shares (averages) are from Lawson et al. (2012); light blue – shares for *Sb* males, light orange – shares for *SB* males.


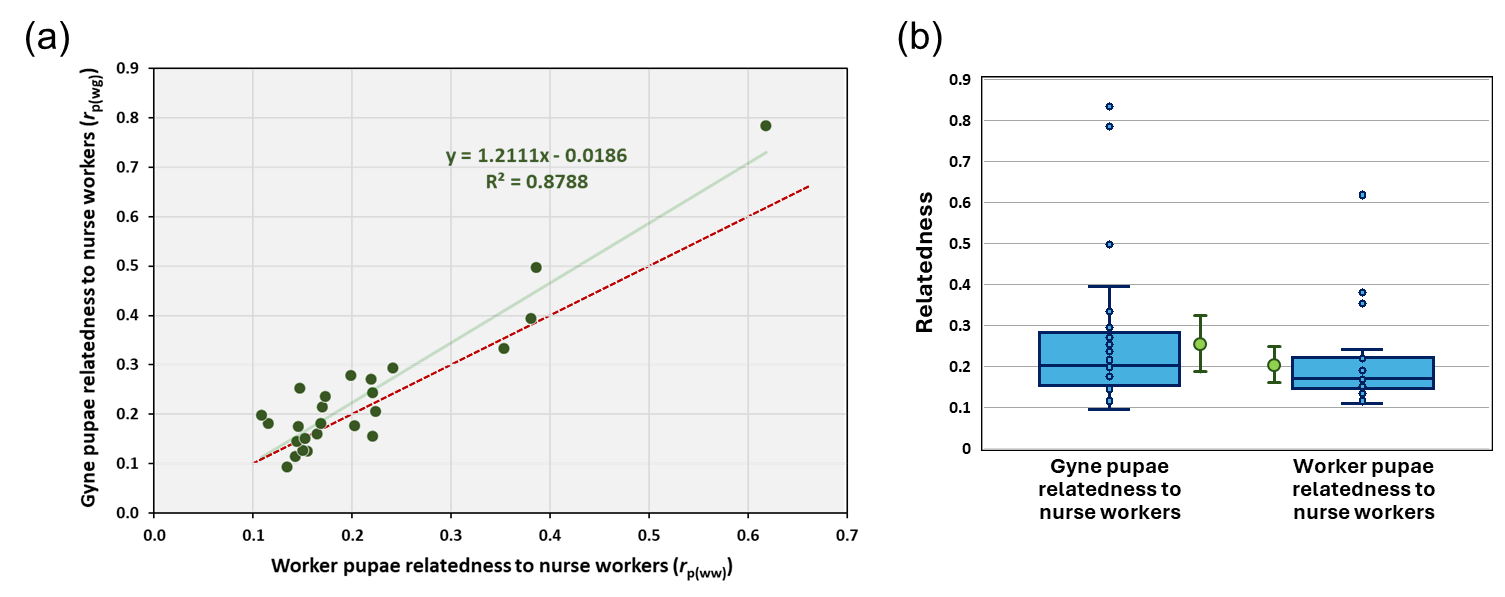


**Figure S17. Relatedness of pupae of each caste to the adult nurse workers that reared them.** (a) Relatedness values for 25 samples from ten colonies (*GA-2023* dataset) using data from sampling points for which >12 pupae of each caste were genotyped (*n*=7812 worker and 3034 gyne pupae). Relatedness of pupae of each caste to the workers that nursed them is similar between the castes across the range of relatedness values, as indicated by the slope of the green fitted least squares regression line close to one and the *y*-intercept close to zero. Nurse/worker relatedness explains almost 90% of the variance in nurse/gyne relatedness (*R*^2^=0.88). The red line depicts perfect congruence between the paired values of relatedness for the two castes. (b) Boxplots showing distributions of relatedness values between offspring pupae of each caste and nurse workers [data as in (a)]. Horizontal lines in boxes are medians. The two distributions do not differ significantly from one another (1-tailed bootstrap test for difference in relatedness values, 1000 iterations, *P*=0.10). Green dots and bars next to boxplots indicate mean values and 95% CIs based on 1000 bootstraps of data that include additional sampling points for which data from only one caste were available (*n*=8185 worker and 3078 gyne pupae); these two distributions do not differ significantly (one-tailed Mann-Whitney *U* test, *P*=0.11).


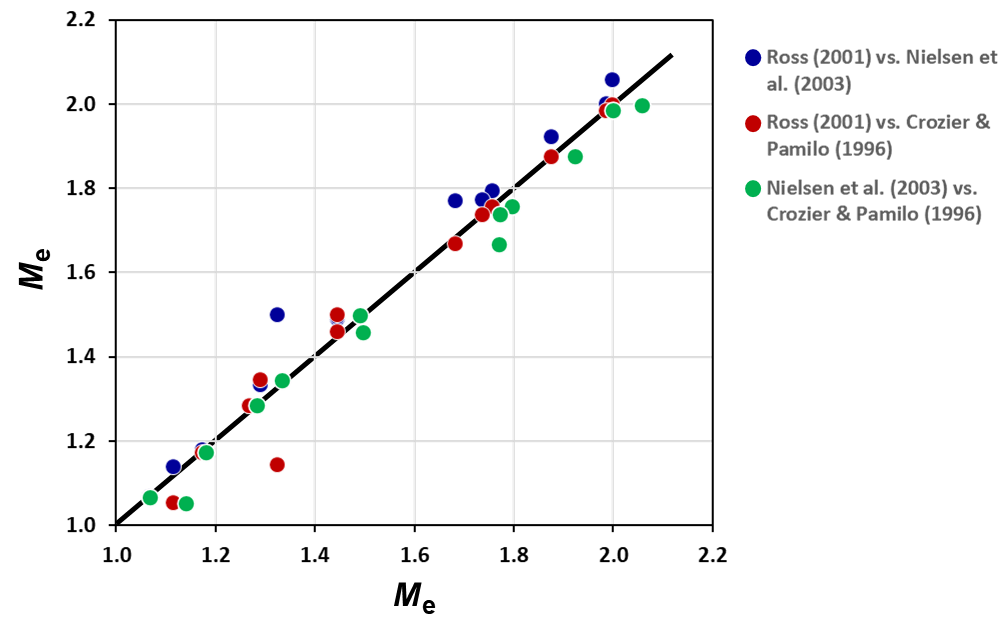


**Figure S18. Comparison of three estimators of *M*_e_ using the direct estimation approach (Method S6).** Estimates for 14 polyandrous queens are based on microsatellite genotypes at 11 loci for 1139 diploid embryos/pupae (*GA-2018* and *GA-2023* datasets). Data for progeny of both castes pooled across all four primary sampling points were extracted from the latter dataset. For each queen, values are shown for each pair of the three estimators. The line depicts complete concordance in values calculated from each pair of estimators.


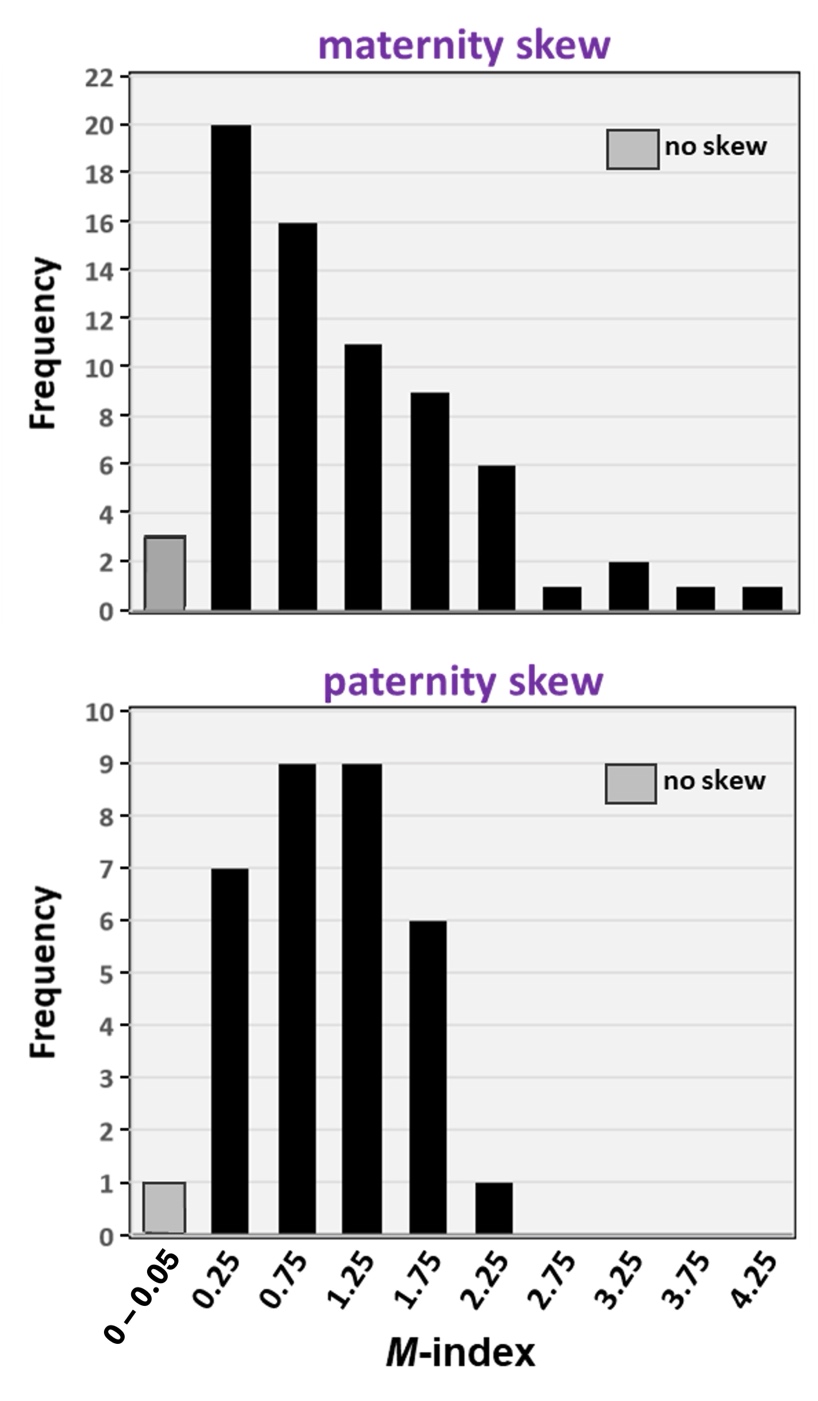


**Figure S19.** **Distributions of *M*-index scores for skew for queens (mothers) and the male mates of polyandrous queens (fathers) in all samples.** All diploid offspring (both castes) are included. The average values for the two distributions do not differ significantly (Mann-Whitney U-test, *P* = 0.445, 1-tail), although the highest values are limited to maternity skew. Averages for maternity and paternity skew distributions for each caste considered separately also do not differ significantly (Mann-Whitney U-tests, both *P* > 0.075, 1-tail). Data are from *GA-2018* and *GA-2023* datasets. *N*=70 sampling instances for maternity skew and *N*=33 for paternity skew. Midpoint values for each bin are shown on the x-axis, except for the first column.


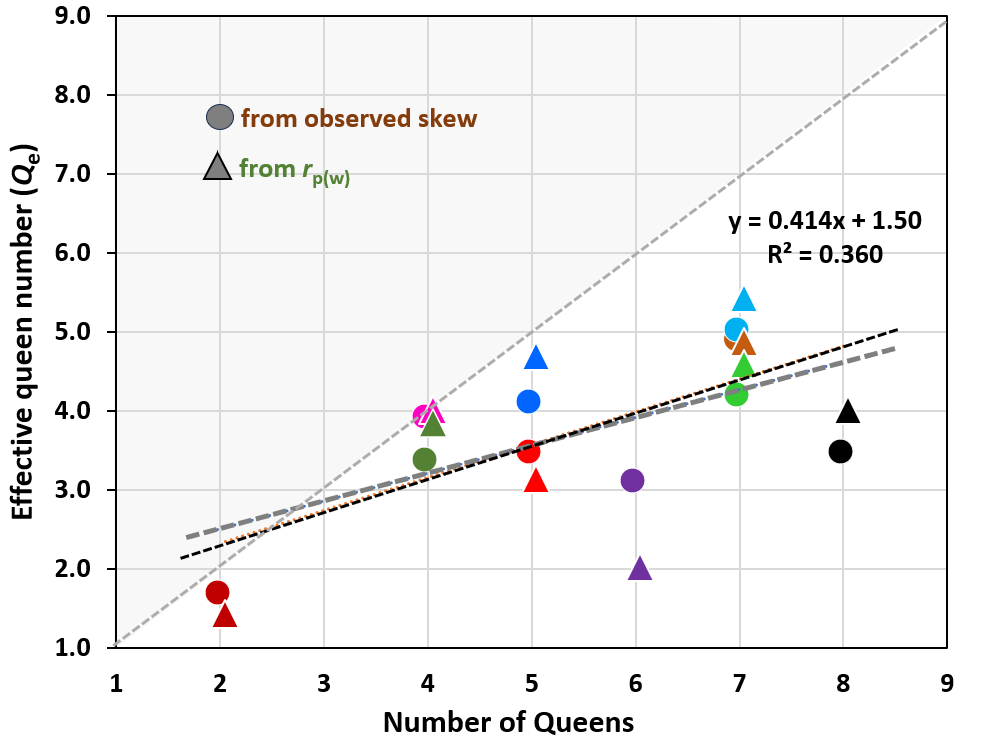


**Figure S20.** **Comparison of effective numbers of mother queens (*Q***_e_**) and actual colony queen numbers.** *Q*_e_ was estimated in two ways, directly from the observed skew and indirectly from *r*_p(w)_, using the genotypes of worker pupae in ten laboratory colonies (*GA-2023* dataset, sampling point 2 [representative of other samples], *n*=1347). The number of mated queens was counted 20-25 days before the pupae were collected, at the approximate time when the eggs giving rise to these pupae would have been laid (Method S1, Movie S1). Values for each colony have differently colored symbols, with the symbol shapes indicating which of the two methods was used to estimate *Q*_e_. Fitted least squares regression lines are shown for the results from the different methods, with the regression equation shown for the “from observed skew” (direct estimate) line. All of the points fall on the white half of the plot, as expected when maternity skew depresses *Q*_e_ relative to the actual queen number. For the value *r*_p(w)_=0.20, the approximate maximum value measured in diverse invasive polygyne populations, we obtain *Q*_e_=3.75 using equation (1) of Ross (2001). From the levels of maternity skew estimated empirically and shown here, this corresponds to about six actual queens per colony.

# References

Ascunce, M. S., Bouwma, A. M., & Shoemaker, D. (2009). Characterization of 24 microsatellite markers in 11 species of fire ants in the genus *Solenopsis* (Hymenoptera: Formicidae). *Molecular Ecology Resources*, *9*(6), 1475–1479. https://doi.org/10.1111/j.1755-0998.2009.02688.x

Ascunce, M. S., Yang, C.-C., Oakey, J., Calcaterra, L., Wu, W.-J., Shih, C.-J., Goudet, J., Ross, K. G., & Shoemaker, D. (2011). Global Invasion History of the Fire Ant *Solenopsis* *invicta*. *Science*, *331*(6020), 1066–1068. https://doi.org/10.1126/science.1198734

Cassill, D. L., & Tschinkel, W. R. (1999). Task selection by workers of the fire ant Solenopsis invicta. *Behavioral Ecology and Sociobiology*, *45*(3), 301–310. https://doi.org/10.1007/s002650050565

Crozier, R. H., & Pamilo, P. (1996). *Evolution of Social Insect Colonies: Sex Allocation and Kin Selection*. Oxford University Press.

Csilléry, K., Johnson, T., Beraldi, D., Clutton-Brock, T., Coltman, D., Hansson, B., Spong, G., & Pemberton, J. M. (2006). Performance of marker-based relatedness estimators in natural populations of outbred vertebrates. *Genetics*, *173*(4), 2091–2101. https://doi.org/10.1534/genetics.106.057331

Fisher, M. A. (2013). *The roles of genetic diversity and SINV-2 viral infection in fitness of the invasive fire ant Solenopsis invicta*. University of Georgia. https://esploro.libs.uga.edu/esploro/outputs/doctoral/The-roles-of-genetic-diversity-and/9949334579502959

Fletcher, D. J. C., Blum, M. S., Whitt, T. V., & Temple, N. (1980). Monogyny and polygyny in the fire ant, *Solenopsis* *invicta*. *Annals of the Entomological Society of America*, *73*(6), 658–661. https://doi.org/10.1093/aesa/73.6.658

Glancey, B. M., & Lofgren, C. S. (1988). Adoption of Newly-Mated Queens: A Mechanism for Proliferation and Perpetuation of Polygynous Red Imported Fire Ants, Solenopsis invicta Buren. *The Florida Entomologist*, *71*(4), 581. https://doi.org/10.2307/3495016

Goodisman, M. A. D., & Ross, K. G. (1996). Relationship of queen number and worker size in polygyne colonies of the fire ant Solenopsis invicta. *Insectes Sociaux*, *43*(3), 303–307. https://doi.org/10.1007/BF01242931

Goodisman, M. A. D., & Ross, K. G. (1998). A test of queen recruitment models using nuclear and mitochondrial markers in the fire ant *Solenopsis* *invicta*. *Evolution*, *52*(5), 1416–1422. https://doi.org/10.1111/j.1558-5646.1998.tb02023.x

Goodisman, M. A. D., & Ross, K. G. (1999). Queen recruitment in a multiple-queen population of the fire ant *Solenopsis* *invicta*. *Behavioral Ecology*, *10*(4), 428–435. https://doi.org/10.1093/beheco/10.4.428

Goodisman, M., & Ross, K. (1997). Relationship of queen number and queen relatedness in multiple-queen colonies of the fire ant *Solenopsis* *invicta*. *Ecological Entomology*, *22*(2), 150–157. https://doi.org/10.1046/j.1365-2311.1997.t01-1-00052.x

Goudet, J., Kay, T., & Weir, B. S. (2018). How to estimate kinship. *Molecular Ecology*, *27*(20), 4121–4135. https://doi.org/10.1111/mec.14833

Guo, S. W., & Thompson, E. A. (1992). Performing the exact test of hardy-weinberg proportion for multiple alleles. *Biometrics*, *48*(2), 361–372. https://doi.org/10.2307/2532296

Hannonen, M., Helanterä, H., & Sundström, L. (2004). Habitat age, breeding system and kinship in the ant *Formica* *fusca*. *Molecular Ecology*, *13*(6), 1579–1588. https://doi.org/10.1111/j.1365-294X.2004.02136.x

Hedrick, P. W. (2005). *Genetics of Populations*. Jones & Bartlett Learning.

Hettesheimer, D. R., Zeng, H., Hunt, B. G., & Ross, K. G. (2024). Biased social chromosome transmission in males of the fire ant Solenopsis invicta. *G3 Genes|Genomes|Genetics*, jkae289. https://doi.org/10.1093/g3journal/jkae289

Huang, K., Guo, S. T., Shattuck, M. R., Chen, S. T., Qi, X. G., Zhang, P., & Li, B. G. (2015). A maximum-likelihood estimation of pairwise relatedness for autopolyploids. *Heredity*, *114*(2), 133–142. https://doi.org/10.1038/hdy.2014.88

Huang, K., Ritland, K., Guo, S., Shattuck, M., & Li, B. (2014). A pairwise relatedness estimator for polyploids. *Molecular Ecology Resources*, *14*(4), 734–744. https://doi.org/10.1111/1755-0998.12217

Jouvenaz, D. P., Allen, G. E., Banks, W. A., & Wojcik, D. P. (1977). A survey for pathogens of fire ants, *Solenopsis* spp., in the Southeastern United States. *The Florida Entomologist*, *60*(4), 275–279. https://doi.org/10.2307/3493922

Kalinowski, S. T., & Taper, M. L. (2006). Maximum likelihood estimation of the frequency of null alleles at microsatellite loci. *Conservation Genetics*, *7*(6), 991–995. https://doi.org/10.1007/s10592-006-9134-9

Kalinowski, S. T., Wagner, A. P., & Taper, M. L. (2006). ml-relate: A computer program for maximum likelihood estimation of relatedness and relationship. *Molecular Ecology Notes*, *6*(2), 576–579. https://doi.org/10.1111/j.1471-8286.2006.01256.x

Keller, L., & Ross, K. G. (1998). Selfish genes: A green beard in the red fire ant. *Nature*, *394*(6693), 573–575. https://doi.org/10.1038/29064

Krieger, M. J. B., Ross, K. G., Chang, C. W. Y., & Keller, L. (1999). Frequency and origin of triploidy in the fire ant *Solenopsis* *invicta*. *Heredity*, *82*(2), 142–150. https://doi.org/10.1038/sj.hdy.6884600

Kronauer, D. J. C., Berghoff, S. M., Powell, S., Denny, A. J., Edwards, K. J., Franks, N. R., & Boomsma, J. J. (2006). A reassessment of the mating system characteristics of the army ant Eciton burchellii. *Naturwissenschaften*, *93*(8), 402–406. https://doi.org/10.1007/s00114-006-0121-2

Kümmerli, R., & Keller, L. (2007a). Contrasting population genetic structure for workers and queens in the putatively unicolonial ant *Formica* *exsecta*. *Molecular Ecology*, *16*(21), 4493–4503. https://doi.org/10.1111/j.1365-294X.2007.03514.x

Kümmerli, R., & Keller, L. (2007b). Extreme reproductive specialization within ant colonies: Some queens produce males whereas others produce workers. *Animal Behaviour*, *74*(5), 1535–1543. https://doi.org/10.1016/j.anbehav.2007.03.014

Lawson, L. P., Vander Meer, R. K., & Shoemaker, D. (2012). Male reproductive fitness and queen polyandry are linked to variation in the supergene Gp-9 in the fire ant *Solenopsis* *invicta*. *Proceedings of the Royal Society B: Biological Sciences*, *279*(1741), 3217–3222. https://doi.org/10.1098/rspb.2012.0315

Levene, H. (1949). On a matching problem arising in genetics. *The Annals of Mathematical Statistics*, *20*(1), 91–94.

Lofgren, C. S., Banks, W. A., & Glancey, B. M. (1975). Biology and control of imported fire ants. *Annual Review of Entomology*, *20*(1), 1–30. https://doi.org/10.1146/annurev.en.20.010175.000245

Loope, K. J., Chien, C., & Juhl, M. (2014). Colony size is linked to paternity frequency and paternity skew in yellowjacket wasps and hornets. *BMC Evolutionary Biology*, *14*(1), 277. https://doi.org/10.1186/s12862-014-0277-x

McGuire, D., Sankovitz, M., & Purcell, J. (2022). A novel distribution of supergene genotypes is present in the socially polymorphic ant Formica neoclara. *BMC Ecology and Evolution*, *22*(1), 47. https://doi.org/10.1186/s12862-022-02001-0

Milligan, B. G. (2003). Maximum-likelihood estimation of relatedness. *Genetics*, *163*(3), 1153–1167. https://doi.org/10.1093/genetics/163.3.1153

Nielsen, R., Tarpy, D. R., & Reeve, H. K. (2003). Estimating effective paternity number in social insects and the effective number of alleles in a population. *Molecular Ecology*, *12*(11), 3157–3164. https://doi.org/10.1046/j.1365-294X.2003.01994.x

O’Neal, J., & Markin, G. P. (1975). Brood development of the various castes of the imported fire ant, *Solenopsis* *invicta* Buren (Hymenoptera: Formicidae). *Journal of the Kansas Entomological Society*, *48*(2), 152–159.

Orr, S. E., Hedrick, N. A., Murray, K. A., Pasupuleti, A. K., & Goodisman, M. A. D. (2024). Novel insights into paternity skew in a polyandrous social wasp. *Insect Science*. https://doi.org/10.1111/1744-7917.13343

Pamilo, P., & Crozier, R. H. (1996). Reproductive Skew Simplified. *Oikos*, *75*(3), 533–535. https://doi.org/10.2307/3545895

Porter, S. D. (1988). Impact of temperature on colony growth and developmental rates of the ant, *Solenopsis invicta*. *Journal of Insect Physiology*, *34*(12), 1127–1133. https://doi.org/10.1016/0022-1910(88)90215-6

Porter, S. D., & Tschinkel, W. R. (1985). Fire ant polymorphism: The ergonomics of brood production. *Behavioral Ecology and Sociobiology*, *16*(4), 323–336. https://doi.org/10.1007/BF00295545

Powell, J. E., Visscher, P. M., & Goddard, M. E. (2010). Reconciling the analysis of IBD and IBS in complex trait studies. *Nature Reviews Genetics*, *11*(11), 800–805. https://doi.org/10.1038/nrg2865

Queller, D. C. (1993). Genetic relatedness and its components in polygynous colonies of social insects. *Queen Number and Sociality in Insects. Oxford University Press, Oxford*, 132–152.

Ross, C. T., Jaeggi, A. V., Borgerhoff Mulder, M., Smith, J. E., Smith, E. A., Gavrilets, S., & Hooper, P. L. (2020). The multinomial index: A robust measure of reproductive skew. *Proceedings of the Royal Society B: Biological Sciences*, *287*(1936), 20202025. https://doi.org/10.1098/rspb.2020.2025

Ross, K. G. (1988). Differential reproduction in multiple-queen colonies of the fire ant *Solenopsis* *invicta* (Hymenoptera: Formicidae). *Behavioral Ecology and Sociobiology*, *23*(6), 341–355.

Ross, K. G. (1993). The breeding system of the fire ant *Solenopsis* *invicta*: Effects on colony genetic structure. *The American Naturalist*, *141*(4), 554–576.

Ross, K. G. (2001). Molecular ecology of social behaviour: Analyses of breeding systems and genetic structure. *Molecular Ecology*, *10*(2), 265–284. https://doi.org/10.1046/j.1365-294x.2001.01191.x

Ross, K. G., & Fletcher, D. J. C. (1985). Comparative study of genetic and social structure in two forms of the fire ant *Solenopsis* *invicta* (Hymenoptera: Formicidae). *Behavioral Ecology and Sociobiology*, *17*(4), 349–356.

Ross, K. G., & Shoemaker, D. (2018). Unexpected patterns of segregation distortion at a selfish supergene in the fire ant *Solenopsis* *invicta*. *BMC Genetics*, *19*(1), 101. https://doi.org/10.1186/s12863-018-0685-9

Ross, K. G., & Shoemaker, D. D. (1997). Nuclear and mitochondrial genetic structure in two social forms of the fire ant *Solenopsis* *invicta*: Insights into transitions to an alternate social organization. *Heredity*, *78*(6), Article 6. https://doi.org/10.1038/hdy.1997.98

Ross, K. G., Vargo, E. L., & Keller, L. (1996). Social evolution in a new environment: The case of introduced fire ants. *Proceedings of the National Academy of Sciences*, *93*(7), 3021–3025. https://doi.org/10.1073/pnas.93.7.3021

Rousset, F., & Raymond, M. (1995). Testing heterozygote excess and deficiency. *Genetics*, *140*(4), 1413–1419. https://doi.org/10.1093/genetics/140.4.1413

Shoemaker, D. D., Deheer, C. J., Krieger, M. J. B., & Ross, K. G. (2006). Population Genetics of the Invasive Fire Ant *Solenopsis* *invicta* (Hymenoptera: Formicidae) in the United States. *Annals of the Entomological Society of America*, *99*(6), 1213–1233. https://doi.org/10.1603/0013-8746(2006)99[1213:PGOTIF]2.0.CO;2

Trible, W., & Ross, K. G. (2016). Chemical communication of queen supergene status in an ant. *Journal of Evolutionary Biology*, *29*(3), 502–513. https://doi.org/10.1111/jeb.12799

Trontti, K., Aron, S., & Sundström, L. (2005). Inbreeding and kinship in the ant Plagiolepis pygmaea. *Molecular Ecology*, *14*(7), 2007–2015. https://doi.org/10.1111/j.1365-294X.2005.02529.x

Tschinkel, W. R. (2006). *The Fire Ants*. Harvard University Press.

Vargo, E. L., & Fletcher, D. J. C. (1987). Effect of queen number on the production of sexuals in natural populations of the fire ant, Solenopsis invicta. *Physiological Entomology*, *12*(1), 109–116. https://doi.org/10.1111/j.1365-3032.1987.tb00729.x

Vargo, E. L., & Fletcher, D. J. C. (1989). On the relationship between queen number and fecundity in polygyne colonies of the fire ant *Solenopsis* *invicta*. *Physiological Entomology*, *14*(2), 223–232. https://doi.org/10.1111/j.1365-3032.1989.tb00955.x

Wade, M. J. (1985). The influence of multiple inseminations and multiple foundresses on social evolution. *Journal of Theoretical Biology*, *112*(1), 109–121. https://doi.org/10.1016/S0022-5193(85)80119-3

Wang, J. (2014). Marker‐based estimates of relatedness and inbreeding coefficients: An assessment of current methods. *Journal of Evolutionary Biology*, *27*(3), 518–530. https://doi.org/10.1111/jeb.12315

Waples, R. K., Albrechtsen, A., & Moltke, I. (2019). Allele frequency-free inference of close familial relationships from genotypes or low-depth sequencing data. *Molecular Ecology*, *28*(1), 35–48. https://doi.org/10.1111/mec.14954

Weir, B. S., Anderson, A. D., & Hepler, A. B. (2006). Genetic relatedness analysis: Modern data and new challenges. *Nature Reviews Genetics*, *7*(10), 771–780. https://doi.org/10.1038/nrg1960

Weir, B. S., & Cockerham, C. C. (1984). Estimating F-statistics for the analysis of population structure. *Evolution*, *38*(6), 1358–1370. https://doi.org/10.2307/2408641

Weir, B. S., & Goudet, J. (2017). A unified characterization of population structure and relatedness. *Genetics*, *206*(4), 2085–2103. https://doi.org/10.1534/genetics.116.198424

Wojcik, D. P., Burges, R. J., Blanton, C. M., & Focks, D. A. (2000). An improved and quantified technique for marking individual fire ants (Hymenoptera: Formicidae). *The Florida Entomologist*, *83*(1), 74–78. https://doi.org/10.2307/3496231

1. We use the term gyne to refer to a morphological queen that has not yet shed her wings, mated, or begun laying eggs (any life stage). [↑](#footnote-ref-2)
